# Autism candidate gene *DIP2A* regulates spine morphogenesis via acetylation of cortactin

**DOI:** 10.1371/journal.pbio.3000461

**Published:** 2019-10-10

**Authors:** Jun Ma, Lu-Qing Zhang, Zi-Xuan He, Xiao-Xiao He, Ya-Jun Wang, You-Li Jian, Xin Wang, Bin-Bin Zhang, Ce Su, Jun Lu, Bai-Qu Huang, Yu Zhang, Gui-Yun Wang, Wei-Xiang Guo, De-Lai Qiu, Lin Mei, Wen-Cheng Xiong, Yao-Wu Zheng, Xiao-Juan Zhu

**Affiliations:** 1 Key Laboratory of Molecular Epigenetics, Institute of Genetics and Cytology, Northeast Normal University, Changchun, China; 2 State Key Laboratory for Molecular and Developmental Biology, Institute of Genetics and Developmental Biology, Chinese Academy of Sciences, Beijing, China; 3 School of Life Sciences, Yunnan University, Kunming, China; 4 Key Laboratory of Cellular Function and Pharmacology of Jilin Province, Yanbian University, Yanji, China; 5 Department of Neurosciences, Case Western Reserve University, Cleveland, Ohio, United States of America; Stanford University School of Medicine, UNITED STATES

## Abstract

Dendritic spine development is crucial for the establishment of excitatory synaptic connectivity and functional neural circuits. Alterations in spine morphology and density have been associated with multiple neurological disorders. Autism candidate gene disconnected-interacting protein homolog 2 A (*DIP2A*) is known to be involved in acetylated coenzyme A (Ac-CoA) synthesis and is primarily expressed in the brain regions with abundant pyramidal neurons. However, the role of DIP2A in the brain remains largely unknown. In this study, we found that deletion of *Dip2a* in mice induced defects in spine morphogenesis along with thin postsynaptic density (PSD), and reduced synaptic transmission of pyramidal neurons. We further identified that DIP2A interacted with cortactin, an activity-dependent spine remodeling protein. The binding activity of DIP2A-PXXP motifs (P, proline; X, any residue) with the cortactin-Src homology 3 (SH3) domain was critical for maintaining the level of acetylated cortactin. Furthermore, *Dip2a* knockout (KO) mice exhibited autism-like behaviors, including excessive repetitive behaviors and defects in social novelty. Importantly, acetylation mimetic cortactin restored the impaired synaptic transmission and ameliorated repetitive behaviors in these mice. Altogether, our findings establish an initial link between *DIP2A* gene variations in autism spectrum disorder (ASD) and highlight the contribution of synaptic protein acetylation to synaptic processing.

## Abstract 2

The autism candidate gene DIP2A is known to be involved in the synthesis of acetylated coenzyme A, but its precise role in the brain remains largely unknown. This study shows that loss of DIP2A in mice results in an imbalance in the acetylation of the synaptic protein cortactin, causing defects in spine morphogenesis and synaptic transmission that may establish a link to autism spectrum disorders.

## Introduction

Dendritic spines are small protrusions that represent the postsynaptic component of most excitatory synapses. Changes in spine shape and density have been considered as a hallmark of synaptic plasticity, which is associated with learning, aging, and neurodevelopmental disorders [1–3]. Synaptic plasticity is dependent on the actin dynamics in dendritic spines [4–6]. Cortactin is an important regulator of actin nucleation that has been shown to impact spine morphology maintenance and activity-dependent synaptic plasticity [7,8]. Recent evidence implicates that acetylation-regulated cortactin activity plays a critical role in spine development and synaptogenesis [9]; however, the precise mechanism is not fully elucidated.

The members of the disconnected (disco)-interacting protein (DIP2) family are evolutionarily conserved in multiple organisms from *Caenorhabditis elegans* to humans, which is reported to maintain the neurite morphology of mature neurons in *C*. *elegans* [10] and regulate the axonal guidance and bifurcation of mushroom body neurons in *Drosophila* [11]. The DIP2 family includes three members in mammalians, disconnected-interacting protein homolog 2 A (DIP2A), DIP2B, and DIP2C. The murine homolog DIP2A is rich in brain lobes and the ventral cord during early embryonic stages [12]. In adult mice, abundant DIP2A exists in brain regions including the neocortex, hippocampus, amygdala, and cerebellum [13]. Human *DIP2A* gene is located in chromosome 21q22.3. Terminal micro-deletions in this chromosomal region or de novo mutation of *DIP2A* is associated with developmental delay [14], substantial susceptibility to autism spectrum disorder (ASD) [15–18], and dyslexia [19,20]. However, further studies exploring the neuronal consequences and animal behaviors of *Dip2a* mutation are still necessary. In addition, members of the DIP2 family shared the structural domains of one DNA methylation 1–associated protein 1 (DMAP1) binding, along with two adenylate-forming domains (AFDs), which are involved in acetylated coenzyme A (Ac-CoA) synthesis [21], but their downstream effector or function in the brain is poorly understood.

In this study, we found that DIP2A interacted with the postsynaptic actin-binding protein, cortactin. Deletion of *Dip2a* in mice led to altered postsynaptic density (PSD) construction and decreased miniature excitatory postsynaptic current (mEPSC) amplitude with reduced acetylation of cortactin. Furthermore, the mice displayed autism-like behaviors, including excessive repetitive self-grooming, reduced vocalization time, and defective social novelty. Importantly, acetylation mimetic cortactin was found to alleviate synaptic defects as well as stereotypical repetitive behaviors. Our results provide a possible cellular mechanism for the synaptic protein acetylation and ASD-like behaviors associated with *DIP2A* deletion.

## Results

### DIP2A is localized to dendritic spines in excitatory neurons

**Fig 1:**
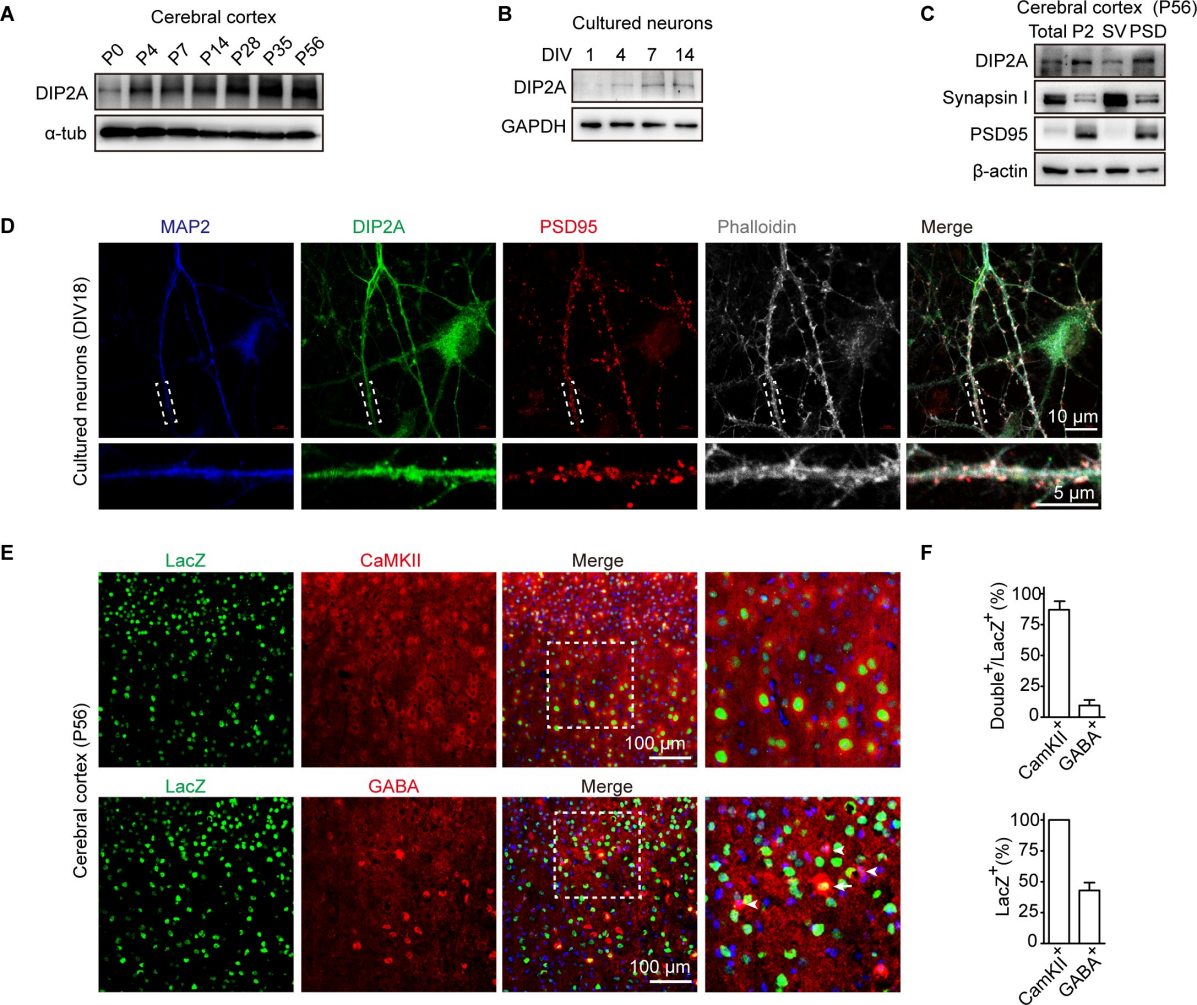
DIP2A is localized to dendritic spines in excitatory neurons. (A) Western blotting showing the level of DIP2A in cerebral cortex constantly increased during postnatal development. (B) Level of DIP2A in cultured neurons. (C) Western blot showing DIP2A in the cortical PSD composition (lane 4). Total, total homogenate; P2, crude synaptosomes; SV, crude synaptic vesicle fraction. (D) Representative image showing the distribution of MAP2 (blue), DIP2A (green), and PSD95 (red) in cultured neurons. Dashed white boxes represent the enlarged view. (E) *Dip2a* β-galactosidase (LacZ) reporter mice (*Dip2a*^*lacZ/+*^) were used to display endogenous DIP2A-expressed cell types. CaMKII and GABA were stained in cortical sections of *Dip2a*^*lacZ/+*^ mice to label excitatory and inhibitory neurons with antibodies, respectively. Arrowhead, GABA immunoreactive neurons; arrow, GABA and LacZ double staining neurons. (F) Relative quantification of LacZ-, CaMKII-, and GABA-positive cells. The underlying data for this figure can be found in S1 Data. CaMKII, Ca^2+^/calmodulin-dependent protein kinase II; DIP2A, disconnected-interacting protein homolog 2 A; DIV, day in vitro; GAPDH, glyceraldehyde-3-phosphate dehydrogenase; LacZ, *Dip2a* β-galactosidase; MAP2, microtubule-associated protein 2; P, postnatal day; PSD, postsynaptic density; P2, crude synaptosomes; SV, crude synaptic vesicle fraction.

Previous studies showed murine *Dip2a* is expressed in the nervous system [12,13]. In the present study, we generated an antibody against DIP2A (anti-DIP2A, S1A Fig) and confirmed its expression in the brain from the embryonic to the adult stage (S1B and S1C Fig). Interestingly, the expression patterns of DIP2A gradually increased after birth in the cerebral cortex (Fig 1A, S1B and S1C Fig), as well as in cultured neurons (Fig 1B and S1D Fig). Furthermore, we isolated synaptosome fractions from murine brain (S1E and S1F Fig) and detected the expression of DIP2A in PSD fraction (Fig 1C). The subcellular localization of DIP2A exhibited that it clustered at dendritic shafts and spines at day 18 in vitro (DIV18) of cultured neurons and partially colocalized with the postsynaptic marker, PSD95 (Fig 1D). These results indicated that expression of DIP2A has correlation with the developmental process.

We then investigated the DIP2A expression pattern in the adult cortex with *Dip2a*-*β-galactosidase* (*LacZ*) mice, in which LacZ was designed with nucleus expression [22]. LacZ was expressed in cortex in either internal or external layer pyramidal neurons and colocalized with layer markers, cut-like homeobox 1 (CUX1) and B cell leukemia/lymphoma 11B (CTIP2) (S2A Fig). Actually, LacZ was expressed in almost all Ca^2+^/calmodulin-dependent protein kinase II (CaMKII)-positive neurons (approximately 100%) throughout the cortex and partially expressed in GABA-positive neurons (41.06 ± 8.19%) (Fig 1E and 1F, S2B Fig). Taken together, these results suggest DIP2A is preferentially expressed in excitatory neurons and prompt us to investigate its function in the synaptic compartment.

### *Dip2a* knockout mice display abnormal dendritic spine morphology and impaired synaptic transmission

*DIP2A* has been reported to be a candidate gene for ASD [15–18] and dyslexia [19,20]; we explored whether *Dip2a* deletion in mice leads to defects in neurodevelopmental disorders. Knocking-out efficacy of *Dip2a* in mice was confirmed by quantifying both mRNA (S3A Fig) and protein expression (S3B Fig). The mice were fertile and appeared grossly normal (S3C–S3E Fig). No abnormalities were detected in brain anatomy and cortical lamination (S3F–S3K Fig).

**Fig 2:**
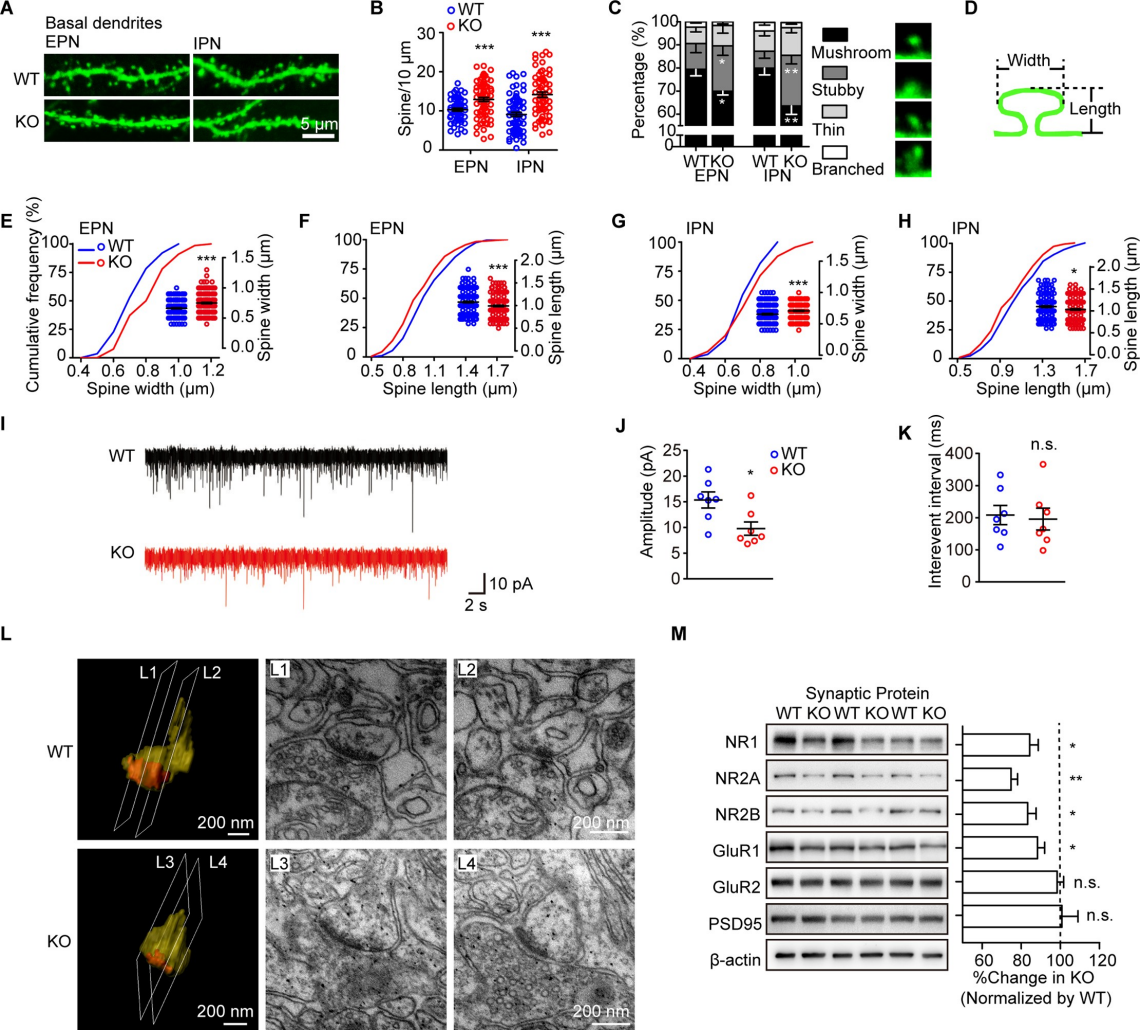
*Dip2a* KO mice display impairment in synaptic morphology and function. (A) Representative images of THY1-GFP–labeled dendrites from EPN and IPN. (B) Increased dendritic spines density of basal dendrite in *Dip2a* KO mice (P56, males; 4 mice per genotype; EPN, WT = 59 neurons, KO = 75 neurons, *t*_132_ = 3.831, ***P = 0.0002; IPN, WT = 71 neurons, KO = 64 neurons, *t*_133_ = 5.819, ***P < 0.0001). (C) Reduced radio of mushroom-like spines (EPN, stubby, *t*_132_ = 2.004, *P = 0.0471; mushroom-like, *t*_132_ = 2.134, *P = 0.0347; IPN, stubby, *t*_133_ = 3.1443, **P = 0.0020; mushroom-like, *t*_133_ = 2.815, **P = 0.0056). (D) Illustration of the measurement of dendritic spine size. (E-H) Quantitative assessment of spine width or length (EPN, WT = 203 spines, KO = 183 spines; E, *t*_383_ **=** 5.991, ***P < 0.0001; F, *t*_381_ **=** 3.632, ***P = 0.0003; IPN, WT = 158 spines, KO = 201 spines; G, *t*_357_ **=** 3.864, ***P = 0.0001; H, *t*_353_ **=** 2.549, *P = 0.0112). (I) Representative whole-cell voltage clamp recordings of spontaneous excitatory postsynaptic currents (sEPSCs) (*n* = 7 mice per genotype). (J) Reduced amplitude of sEPSCs in KO mice (*t*_12_ **=** 2.751, *P = 0.0176). (K) No significant difference was observed in the interventional interval (*t*_12_ **=** 0.2820, n.s. *P =* 0.7828). (L) Examples of 3D reconstruction depicting postsynaptic structure and electron micrographs. (M) Western blot showing the PSD fraction (4 mice per genotype in each experiment; data from 4 independent experiments). Protein levels were normalized to β-actin. The ratio in WT mice was set to 100% (NR1, *t*_18_ = 2.274, *P = 0.0355; NR2A, *t*_10_ = 4.156, **P = 0.0020; NR2B, *t*_10_ = 2.248, *P = 0.0484; GluR1, *t*_18_ = 1.082, *P = 0.0447; GluR2, *t*_18_ = 0.8829, n.s. *P =* 0.3889; PSD95, *t*_10_ = 0.0759, n.s. *P =* 0.9410). Data are represented as mean ± SEM and assessed with two-tailed unpaired *t* test. The underlying data for this figure can be found in S1 Data. *Dip2a*, disconnected-interacting protein homolog 2 A; EPN, external layer of pyramidal neurons; GFP, green fluorescence protein; GluR1, glutamate ionotropic receptor AMPA type subunit 1; IPN, internal layer of pyramidal neurons; KO, knockout; NMDAR, N-methyl-D-aspartate receptor; NR1, NMDAR1; NR2A, NMDAR2; NR2B, NMDAR2B; n.s., no significance; PSD, postsynaptic density; THY1, thymocyte antigen 1; WT, wild-type; 3D, three-dimensional.

To visualize spines, *Dip2a* knockout (KO) mice were crossed with *thymocyte antigen 1* (*Thy1*)*–*green fluorescence protein (*GFP*) mice [23,24] to label neurons randomly with GFP in the cortex, hippocampus, and amygdala (S4A Fig). In the cerebral cortex, neuronal morphology was distinguishable from the external layer of pyramidal neurons (EPN) and the internal layer of pyramidal neurons (IPN) (S4A Fig). The density and morphology of spines were analyzed in each neuron, from secondary or tertiary dendrites. Compared with wild-type (WT) littermates, *Dip2a* KO neurons exhibited increased spine density in basal dendrites by approximately 20% in EPN and 65% in IPN (Fig 2A and 2B). However, no significant difference was found in spine density in apical dendrites (S4B and S4C Fig). Furthermore, spines were categorized based on spine length and the ratio of neck to head width [25] (S1 Table). In the *Dip2a* KO neurons, the proportion of mushroom-like spines in basal dendrites was reduced by approximately 10% in EPN and 17% in IPN (Fig 2C). Meanwhile, stubby spines in basal dendrites were increased, with a significant increase in spine head width and a decrease in spine length (Fig 2D–2H). The morphological abnormalities were not observed in apical dendrites (S4D Fig).

To examine the physiological roles of DIP2A on synaptic function, we performed electrophysiological recordings of IPN in acutely prepared brain slices. The amplitude of spontaneous excitatory postsynaptic current (sEPSC) was significantly decreased in *Dip2a* KO mice (15.35 ± 1.56 pA and 9.77 ± 1.29 pA for WT and KO, respectively; Fig 2I and 2J). There was no significant difference in the frequency of sEPSC between the mice (Fig 2K), indicating that deletion of *Dip2a* led to postsynaptic defects.

Mature spines have a large head area that is proportional to PSD, as well as receptor complement [26]. We next examined the postsynaptic structure by transmission electron microscopy (TEM) and performed three-dimensional (3D) reconstruction using 30 serial TEM sections. It exhibited the enlarged postsynaptic terminal with a distinct neck (yellow) and abundant PSD (orange) in WT mice (Fig 2L1 and 2L2; S1 Movie). However, the postsynaptic structure in KO mice appeared stubby, with flattened PSD (orange, orange, Fig 2L3 and 2L4; S2 Movie). The cross sections for TEM images are indicated. Indeed, numbers of α-amino-3-hydroxy-5-methyl-4-isoxazolepropionic acid receptors (AMPARs) and N-methyl-D-aspartate receptors (NMDARs) directly correlate with PSD size, which is implicated in the synaptic transmission [27,28]. In the PSD fractions of *Dip2a* KO mice (Fig 2M), we found an obvious reduction in the subunit protein levels of NMDAR1 (NR1), NR2A, NR2B, and glutamate ionotropic receptor AMPA type subunit 1 (GluR1). No significant difference was detected in total cerebral cortical lysates (S4E Fig). In ensemble, our results reveal an essential defect in spine morphology and synaptic transmission in the *Dip2a* KO neurons, which may be mediated by, but is not limited to, PSD size and glutamate receptors dysfunction.

### DIP2A binds to cortactin and modulates cortactin acetylation

To explore the regulatory mechanism of DIP2A in dendritic spines, we carried out liquid chromatography–tandem mass spectrometry (LC-MS/MS) to find the potential downstream molecules interacting with DIP2A. One hundred seventeen proteins with anti-FLAG-tag (DYKDDDDK) antibody immunoprecipitated were identified by LC-MS/MS from FLAG-DIP2A–transfected HEK293 cells (S5A Fig and S2 Table). Postsynaptic proteins, cortactin (polypeptide amino acid sequence, YGLFPANYVELR, accession number, Q60598; S3 Table), were among the statistically significant proteins identified as an interaction partner of DIP2A. We focused on it for further research, because as a synaptic F-actin regulator, cortactin interacts with the postsynaptic scaffold protein Src homology 3 (SH3) and multiple ankyrin repeat domains 3 (SHANK3) and is essential in promoting synapse genesis [29–31].

**Fig 3:**
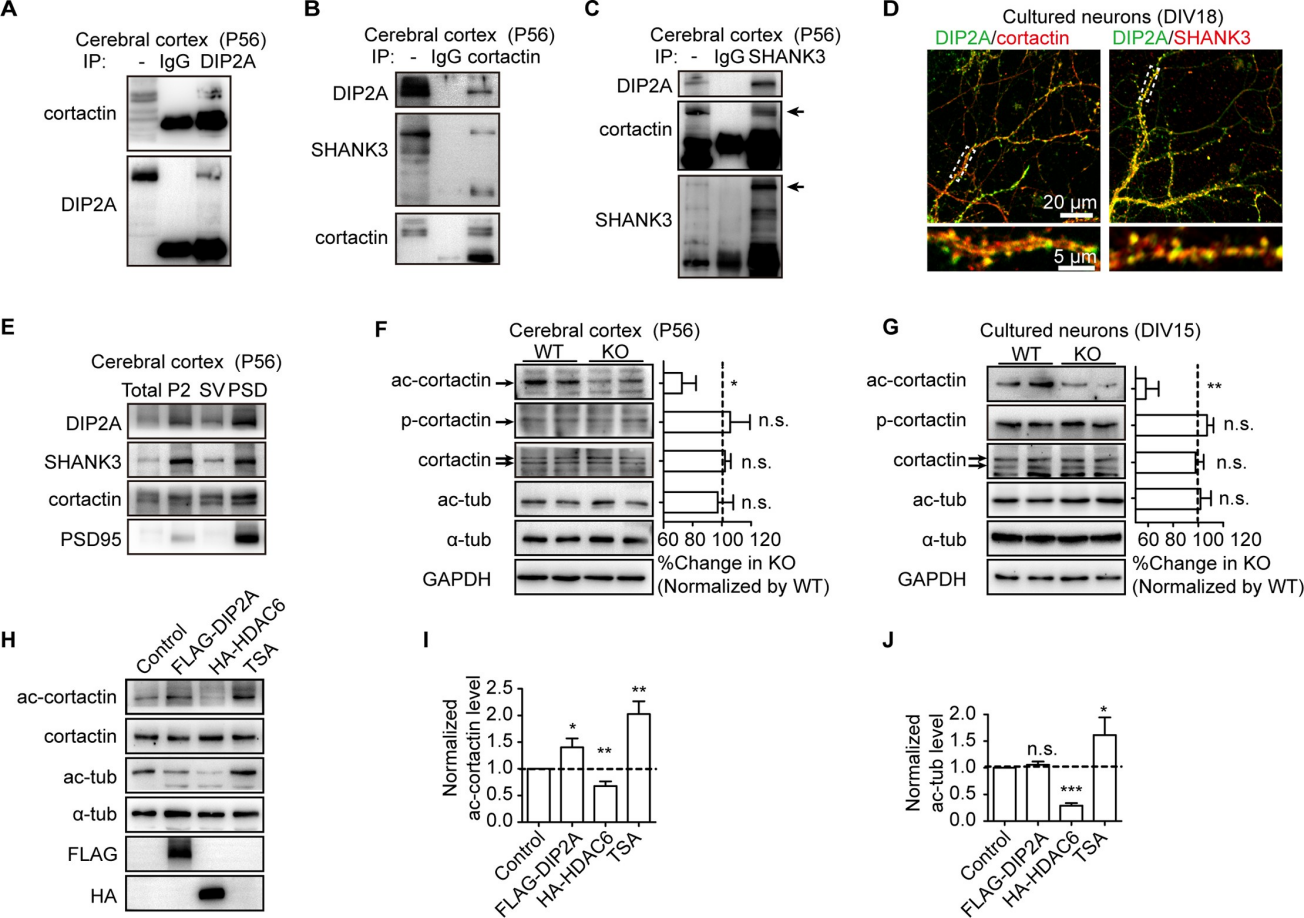
DIP2A interacts with cortactin and modulates cortactin acetylation (ac-cortactin). (A) Interaction between endogenous DIP2A and cortactin from murine brain lysates. (B and C) Interaction between endogenous DIP2A, cortactin, and SHANK3 from murine brain lysates. (D) Representative image showing colocalization of DIP2A (green) and cortactin or SHANK3 (red) in cultured neurons. (E) Western blot showing DIP2A and cortactin, and SHANK3 in the cortical PSD composition (lane 4). Total, total homogenate; P2, crude synaptosomes; SV, crude synaptic vesicle fraction. (F) Western blot and bar graph showing *Dip2a* deletion reduced ac-cortactin without altering levels of total cortactin and phosphorylated cortactin (p-cortactin) (P56, males; *n* = 4 mice per genotype; data from 3 independent experiments). Levels of ac-cortactin and tubulin (ac-tub) were normalized to total cortactin and α-tubulin, respectively. The ratio in WT mice was set to 100% (ac-cortactin, *t*_10_ = 2.320, *P = 0.0428; p-cortactin, *t*_10_ = 0.8637, n.s. *P =* 0.4080; cortactin, *t*_10_ = 1.712, n.s. *P =* 0.1177; ac-tub, *t*_10_ = 0.1943, n.s. *P =* 0.8498). (G) *Dip2a* deletion reduced ac-cortactin without affecting total or p-cortactin levels in cultured cortical neurons (DIV15) (data from 3 independent experiments, approximately 5 × 10^6^ cells per experiment; ac-cortactin, *t*_10_ = 3.258, **P = 0.0086; p-cortactin, *t*_10_ = 0.1551, n.s. *P =* 0.8798; cortactin, *t*_10_ = 0.4124, n.s. *P =* 0.6888; ac-tub, *t*_10_ = 0.1507, n.s. *P =* 0.8832). (H) Western blot showing overexpressed DIP2A in cultured cells with elevated ac-cortactin level. (I and J) Normalized grayscale value in (H) (data from 3 independent experiments and presented as mean ± SEM; one-way ANOVA; I, *F*_3,20_ = 19.13, post hoc LSD, *P = 0.0199, **P = 0.0020 and 0.0016, respectively; J, *F*_3,20_ = 17.76, post hoc LSD, n.s. *P =* 0.3227, ***P < 0.0001, *P = 0.0379). The underlying data for this figure can be found in S1 Data. ac-cortactin, cortactin acetylation; DIP2A, disconnected-interacting protein homolog 2 A; DIV, day in vitro; FLAG, FLAG tag with the sequence DYKDDDDK; GAPDH, glyceraldehyde 3-phosphate dehydrogenase; HA, HA-tag (YPYDVPDYA); HDAC6, histone deacetylase 6; IgG, immunoglobulin G; IP, immunoprecipitation; KO, knockout; LSD, least significant difference; n.s., no significance; p-cortactin, phosphorylated cortactin; PSD, postsynaptic density; P2, crude synaptosomes; SHANK3, SH3 and multiple ankyrin repeat domains 3; SV, crude synaptic vesicle fraction; TSA, trichostatin A; tub, tubulin; WT, wild-type.

We then performed an immunoprecipitation assay with murine brain lysate. As expected, endogenous DIP2A, cortactin, and SHANK3 were detected in one immunoprecipitation complex (Fig 3A–3C). We also observed colocalization of DIP2A with cortactin and SHANK3 in the dendritic spines of cultured neurons (Fig 3D) and found all of them present in the PSD fraction of cortical lysates (Fig 3E). *SHANK3* is a well-characterized risk gene for ASD [32]; however, in *Dip2a* KO mice, we did not detect significant alteration of the SHANK3 protein level (S5B Fig).

Cortactin regulates synapse composition and plasticity with phosphorylated or acetylated modification [33,34]. As a binding partner, DIP2A has been presumed to mediate cortactin. Thus, we examined cortactin expression and modification levels. As shown in (Fig 3F), cortactin acetylation (ac-cortactin) was significantly reduced in *Dip2a* KO murine cortex lysates. However, the total protein expression of cortactin or phosphorylated cortactin was not changed (Fig 3F). Similar results were found in cultured *Dip2a* KO neurons (Fig 3G). Importantly, the effect on acetylation was specific for cortactin, because the acetylation levels of tubulin (ac-tub) and histones H3 and H4 (S5C and S5D Fig) were not altered in *Dip2a* KO mice.

To further illustrate the specific effect of DIP2A on ac-cortactin, we used histone deacetylase 6 (HDAC6), which has broad deacetylation activity, along with trichostatin A (TSA), a deacetylase inhibitor, as positive controls. As expected, overexpression of HDAC6 reduced acetylated levels of both cortactin (Fig 3H and 3I) and tubulin (Fig 3H and 3J) in cultured cells. Contrarily, incubation with TSA greatly increased acetylated cortactin and tubulin. Unlike the broad effect of TSA and HDAC6, overexpression of FLAG-DIP2A selectively increased ac-cortactin. Together, these data strongly suggest DIP2A interacted with cortactin and specifically modulated ac-cortactin.

### Interaction of DIP2A-cortactin is required for ac-cortactin

It was intriguing to find that DIP2A selectively regulated acetylation of cortactin without affecting that of tubulin or histones, implying the DIP2A–cortactin interaction is essential for ac-cortactin. To examine the specific binding region in this context, we co-transfected domain-based DIP2A and cortactin expression constructs (S5E Fig) into cultured HEK293 cells. As shown in S5F and S5G Fig, the N terminus of DIP2A (DIP2A_1–320_) was responsible for its interaction with the cortactin SH3 domain. We identified that binding proteins form HEK293 cells lacking neuron-specific protein profiles; thus, we performed a pull-down assay among cerebral cortex lysate using hexahistidine tag (His)-tagged-DIP2A_1–320_. As expected, cortactin and SHANK3 were detected in the DIP2A_1–320_ complex (S5H Fig). Furthermore, the corresponding lane on SDS-PAGE was subjected to LC-MS/MS, and 16 peptides were identified to confirm the binding of DIP2A with cortactin in vivo (S3 Table).

**Fig 4:**
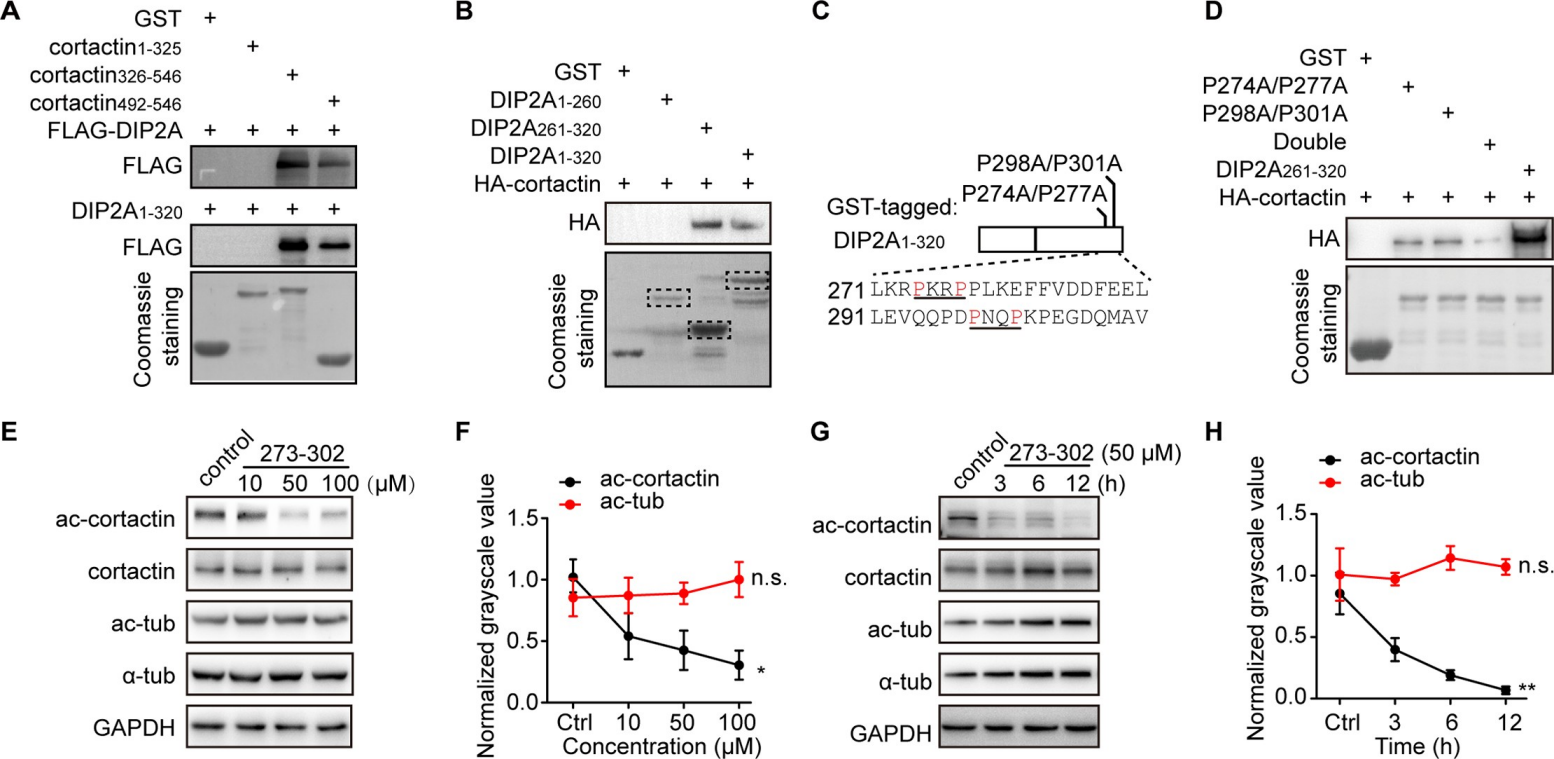
The DIP2A-cortactin interaction is critical for mediating ac-cortactin. (A) The SH3 domain of cortactin (aa 492–546) was required for binding with DIP2A in the GST pull-down assay. (B) The binding assay demonstrated that the DIP2A_261–320_ region with PXXP motifs was large enough to bind cortactin. (C) Diagram of GST-tagged DIP2A_1–320_ with site mutation strategies. Residues 271–310 with PXXP motifs are aligned below. (D) DIP2A-cortactin binding was minimally detectable when both PXXP motifs were simultaneously mutated (double). (E and F) Ac-cortactin levels were decreased in a concentration-dependent pattern (ac-cortactin, Pearson correlation = −0.656, *P = 0.0205; ac-tub, Pearson correlation = 0.279, n.s. *P =* 0.3798). (G and H) Ac-cortactin levels were decreased in time-dependent patterns (ac-cortactin, Pearson correlation = −0.814, **P = 0.0013; ac-tub, Pearson correlation = 0.181, n.s. *P =* 0.5729). The underlying data for this figure can be found in S1 Data. aa, amino acid; ac-cortactin, cortactin acetylation; DIP2A, disconnected-interacting protein homolog 2 A; FLAG, FLAG tag with the sequence DYKDDDDK; GAPDH, glyceraldehyde 3-phosphate dehydrogenase; GST, glutathione S-transferase tag; HA, human influenza hemagglutinin tag (YPYDVPDYA-tag); n.s., no significance; PXXP, proline-rich motif, where X is any residue; SH3, src homology 3; tub, tubulin.

The SH3 domain is a well-known protein–protein interaction module that preferentially recognizes proline (P)-rich sequences with the PXXP motif [35,36]. We found the DIP2A_1–320_ region is rich in proline (approximately 10%) and contains two PXXP motifs between amino acid residues 261 and 320. The binding assay further demonstrated the DIP2A_261–320_ region was sufficient for the cortactin-SH3 binding (Fig 4A and 4B). Obviously, this binding significantly decreased when the proline residues of one of the two PXXP motifs were mutated to alanine (either P274A/P277A or P298A/P301A). Notoriously, this binding was minimally detectable when both PXXP motifs were simultaneously mutated (double) (Fig 4C and 4D). Therefore, the PXXP motifs in DIP2A appear to be critical for its interaction with the SH3 domain of cortactin.

To further evaluate this aspect, we designed a membrane-permeable peptide with a trans-activator of transcription (TAT) assistant sequence [37] (YGRKKRRQRRRRPKRPPLKEFFVDDFEELLEVQQPDPNQPK, in which the underlined section is for TAT). The peptide sequence was derived from the key interaction region (amino acid [aa] 273–302) of DIP2A for cortactin, containing the two PXXP motifs (Fig 4C). When the peptide was incubated in cultured cells to competitively bind with endogenous cortactin, acetylation of the latter decreased in correlation with peptide concentration (Fig 4E and 4F) and incubation time (Fig 4G and 4H). The effects of this peptide were restricted to ac-cortactin and did not affect tubulin (Fig 4E–4H). These findings suggest that the DIP2A-cortactin interaction is required for DIP2A-mediated ac-cortactin.

### DIP2A mediates ac-cortactin with Ac-CoA in a concentration-dependent manner

**Fig 5:**
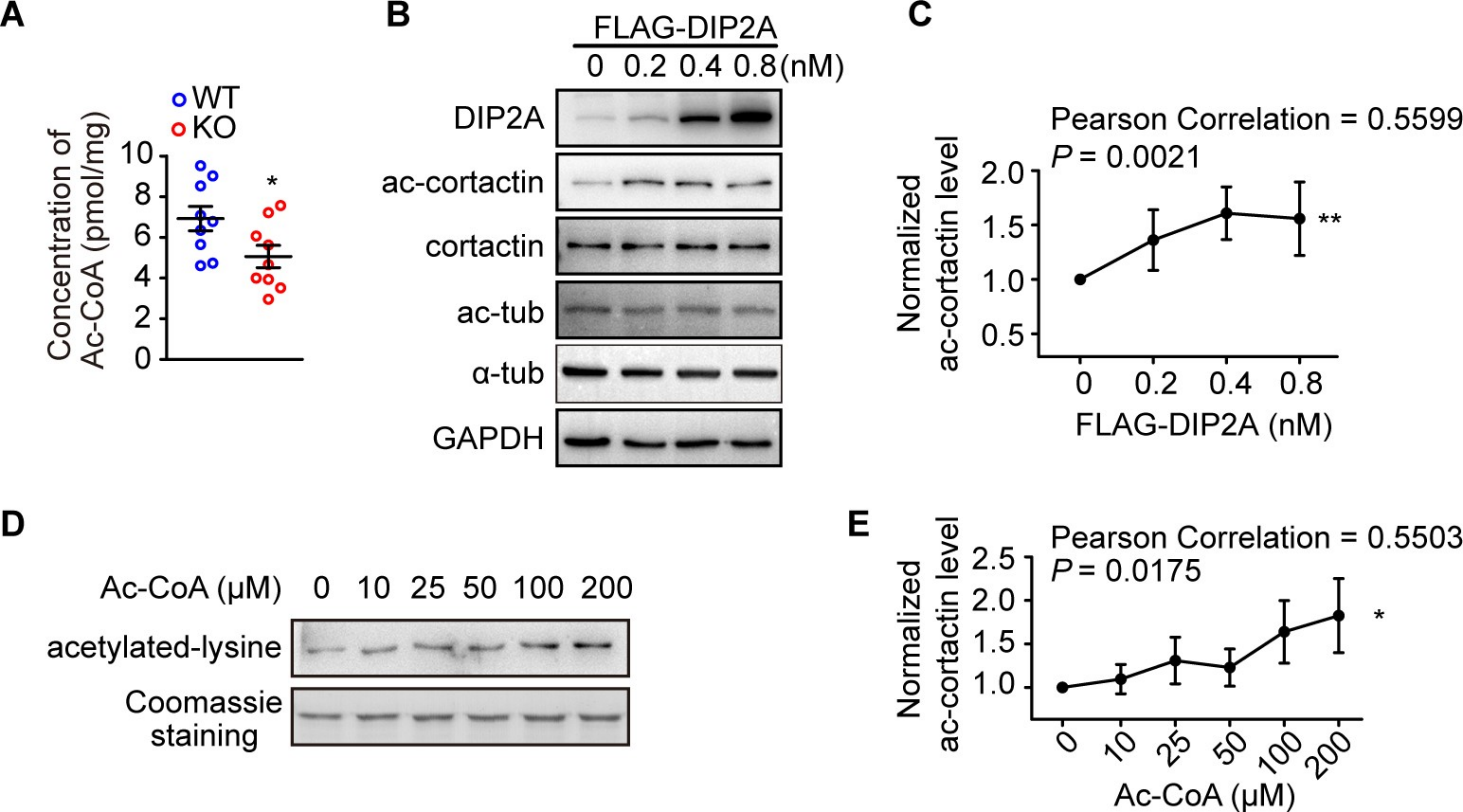
DIP2A-mediated ac-cortactin is dependent on Ac-CoA concentration. (A) Ac-CoA concentration in the cerebral cortex decreased in *Dip2a* KO mice in comparison with WT littermates (P56, males; *n* = 9 mice per genotype; *t*_16_ = 2.291, *P = 0.0359). (B and C) Overexpressed DIP2A in HEK293 cells enhanced ac-cortactin in a dose-dependent manner (data from 4 independent experiments and represented as mean ± SEM). (D and E) Western blot and line graph showing that levels of ac-cortactin increased as the concentration of Ac-CoA increased in in vitro acetylation assays. The underlying data for this figure can be found in S1 Data. Ac-CoA, acetylated coenzyme A; ac-cortactin, cortactin acetylation; DIP2A, disconnected-interacting protein homolog 2 A; KO, knockout; WT, wild-type.

Ac-CoA is an exclusive acetyl donor for lysine residues, and nonenzymatic acetylation has been observed in eukaryotic cells [38]. Our previous study indicates DIP2A is involved in Ac-CoA synthesis [21]. We thus measured Ac-CoA concentration in *Dip2a* KO brain lysate and found a significantly decreased Ac-CoA concentration in comparison with WT littermates (6.92 ± 0.60 pmol/mg and 5.06 ± 0.55 pmol/mg for WT and KO, respectively; Fig 5A). Furthermore, overexpression of DIP2A in cultured cells up-regulated ac-cortactin following a concentration-dependent pattern (Fig 5B and 5C). Importantly, the level of acetylated cortactin clearly increased in an Ac-CoA concentration-dependent manner during in vitro incubation with glutathione S-transferase tagged (GST)-cortactin and Ac-CoA (Fig 5D and 5E). Meanwhile, no detectable changes in relative mRNA levels of several acetyl transferases and deacetylases were found in *Dip2a* KO mice, which have been demonstrated to regulate ac-cortactin (S6A Fig). Unlike lysine acetyltransferase 2B (PCAF), DIP2A did not directly acetylate cortactin in vitro as an acetyltransferase (S6B Fig). Together, these results suggest DIP2A mediated ac-cortactin, relying on the local Ac-CoA concentration.

Next, we identified the acetylation sites of cortactin from the in vitro assays shown in Fig 5D by LC-MS/MS. Eight lysine residues (K107, K152, K171, K181, K193, K235, K309, and K314) were found to be acetylated in the repeat region of cortactin (S6C Fig and S2 Text), providing the basis for the next remediation experiment.

### Acetylation cortactin is critical for DIP2A-regulated synaptic transmission

**Fig 6:**
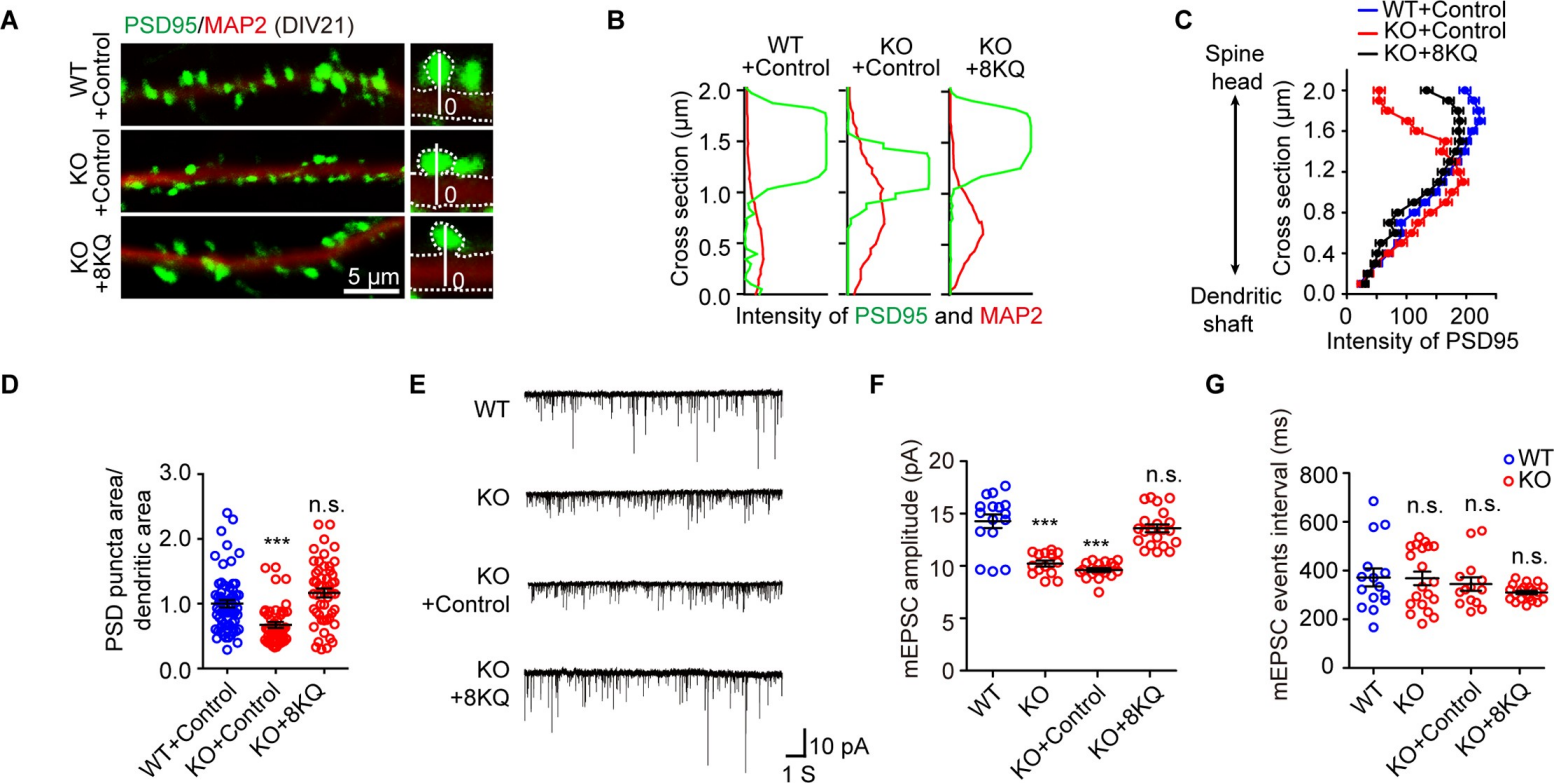
Acetylation mimetic cortactin rescues synaptic defects. (A) Representative image of dendritic segments of cultured neurons infected at DIV15 with Lv-eGFP (control) or Lv-eGFP-cortactin 8KQ, and imaged at DIV21. The right insets showing an example of the quantitative analysis of PSD95 distribution along the 2-μm vertical white line, which proceeds from the base of the dendritic shaft (set as 0) to the outline of PSD95. (B) Traces illustrating the fluorescence intensity of PSD95 (green) along the scanning path in (A). (C) Quantitative analysis of the distribution of PSD95. (D) Scatterplot graph showing the area ratio of PSD95-positive puncta to the total dendritic area (MAP2). Data from 4 independent experiments and represented as mean ± SEM; WT + control = 64 neurons, KO + control = 46 neurons, KO + 8KQ = 51 neurons; one-way ANOVA, *F*_2, 158_ = 16.56; post hoc LSD, ***P < 0.0001, n.s. *P =* 0.0513, related to WT + control. (E) Representative whole-cell voltage clamp traces of mEPSC from cultured neurons held at −70 mV. (F) Scatterplot graph showing mEPSC amplitude. Lv-eGFP-cortactin 8KQ infection of *Dip2a* KO neurons at DIV10 rescued the aberrant mEPSC amplitude (*n* = 16, 14, 18, and 21 neurons, respectively; data from 3 independent experiments; one-way ANOVA, *F*_3, 65_ = 34.16; post hoc LSD, ***P < 0.0001, n.s. *P =* 0.1521). (G) Scatterplot graph showing the equivalent mEPSC frequency (one-way ANOVA, *F*_3, 61_ = 1.01, *P =* 0.13946; post hoc LSD, n.s. *P* > 0.05). The underlying data for this figure can be found in S1 Data. DIV, day in vitro; KO, knockout; LSD, least significant difference; Lv-eGFP, lentivirus carrying enhanced green fluorescent protein tag; MAP2, microtubule-associated protein 2; mEPSC, miniature excitatory postsynaptic current; n.s., no significance; PSD, postsynaptic density; WT, wild-type; 8KQ, acetylation mimetic cortactin with eight lysine replaced with glutamine.

Acetylated cortactin has been previously shown to modulate and promote clustering of the excitatory postsynaptic scaffold protein PSD95, without affecting the total levels of this protein [34]. Consistent with previous research [29], PSD95 clusters were mostly concentrated on spine heads in WT cultured neurons, at an approximate distance of 1–2 μm from the dendrite shaft. However, in *Dip2a* KO neurons, PSD95 clusters were flattened, with an approximate distance of 0.5–1.5 μm from the dendrite shaft (Fig 6A–6C), and the PSD95 punctum area related to total dendritic area dramatically decreased (Fig 6D).

To validate the causal relationship between DIP2A and ac-cortactin, we constructed acetylation mimetic cortactin by mutating all 8 lysine (K) residues to glutamine (Q) residues (cortactin-8KQ), which neutralized positive charges [39]. This was confirmed with anti-human influenza hemagglutinin (HA) tag (YPYDVPDYA) antibodies (S6D Fig) and F-actin co-sedimentation assays (S6E Fig). *Dip2a* KO neurons were then infected with lentivirus (Lv)-cortactin-8KQ. As expected, cortactin-8KQ resolved the abnormal PSD95 clustering at the spine head (Fig 6A–6D). Obviously, Lv-cortactin-8KQ alleviated the defects in the mEPSC amplitude of *Dip2a* KO neurons (WT, 14.72 ± 0.65 pA; KO, 10.23 ± 0.28 pA; KO + control, 9.23 ± 0.18 pA; KO + 8KQ, 13.23 ± 0.38 pA; Fig 6E–6G), which did not affect the amplitude or frequency of mEPSC when it infected WT neurons (S6F–S6H Fig). However, Lv-cortactin that was not acetylated artificially did not totally rescue the defects in mEPSC amplitude of KO neurons (WT + control, 21.99 ± 1.07 pA; KO + control, 11.82 ± 0.31 pA; KO + cortactin, 13.85 ± 0.34 pA; S6I–S6K Fig). This result further demonstrated that DIP2A-mediated acetylation of cortactin is required for proper synaptic transmission.

To further confirm the functional association of DIP2A with PSD95 clustering, the adenovirus-DIP2A inserted into cultured neurons also rescued the synaptic morphology deficits by recovering flattened PSD95 clusters (0.5–1 μm) toward the spine head (0.8–1.8 μm) (S7A–S7D Fig). The amplitude of evoked EPSC was halved in cultured *Dip2a* KO neurons compared with WT neurons (S7E and S7F Fig), suggesting DIP2A played a direct role in the regulation of synaptic activity. Adenovirus-DIP2A also relieved the functional deficits in the mEPSC of KO neurons (S7G–S7I Fig). Together, these results strongly support the hypothesis that DIP2A regulates spine morphology and synaptic transmission through acetylation of cortactin.

### Acetylation mimetic cortactin rescues the repetitive behaviors in *Dip2a* KO mice

Currently identified gene mutations in individuals with ASD appear to have synaptic dysfunction in animal models, as well as social abnormalities and stereotyped repetitive behaviors [40–42]. To explore the relevance of *DIP2A* mutations in ASD, we conducted a battery of behavioral assays in *Dip2a* KO mice and littermate WT mice. These tests are highly relevant to the core diagnostic symptoms of ASD [43] (S4 Table).

**Fig 7:**
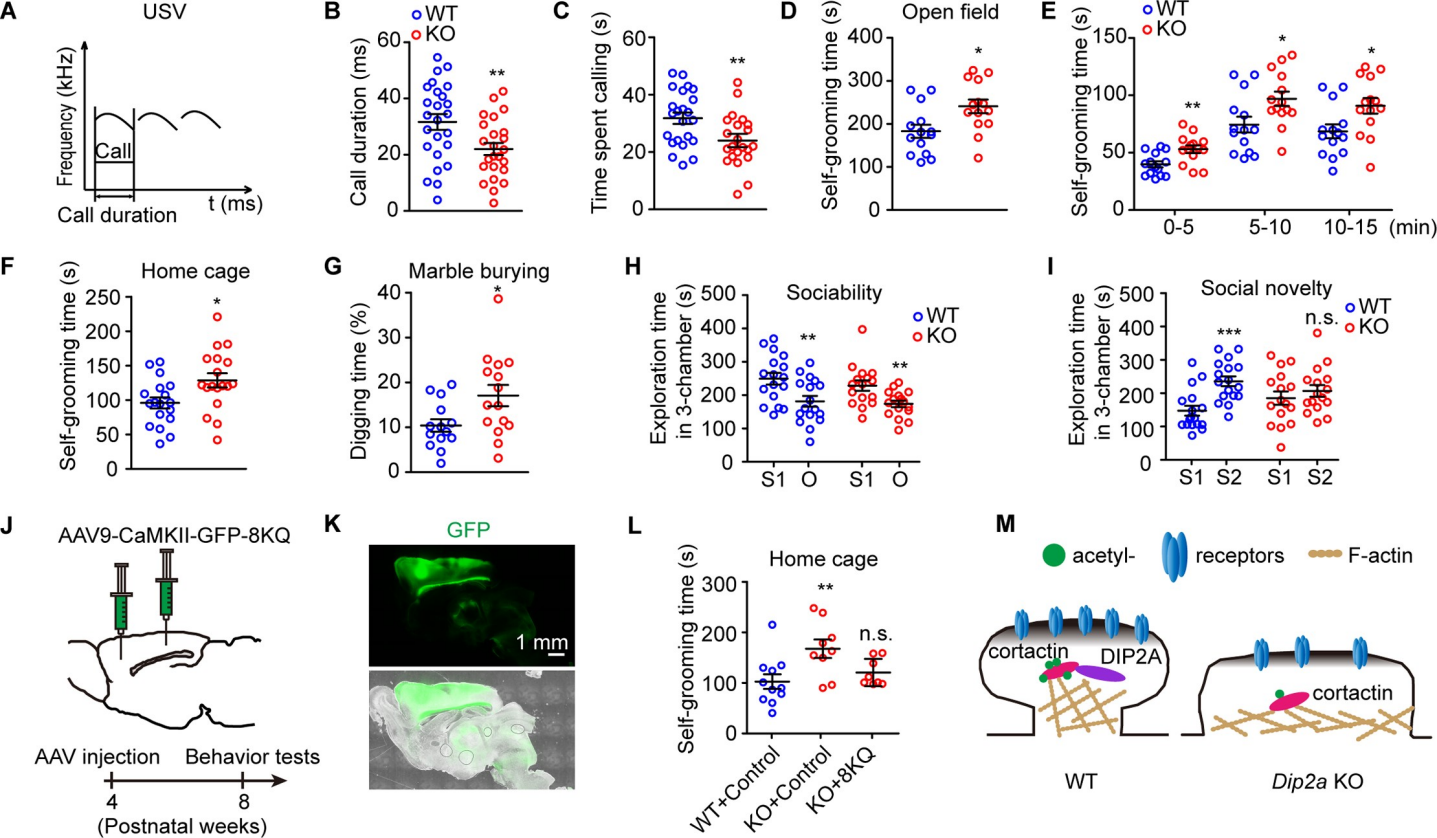
Deletion of *Dip2a* in mice leads to an autism-like phenotype. (A) Diagram of the evaluation of isolation-induced USVs (WT = 25 mice, KO = 24 mice). (B and C) Reduced call duration of USV (B, *t*_47_ **=** 2.711, **P = 0.0093) and calling time (C, *t*_47_ **=** 2.716, **P = 0.0092) in *Dip2a* KO pups. (D and E) *Dip2a* KO mice had increased self-grooming time in the open field (14 mice per genotype; D, *t*_26_ **=** 2.651, *P = 0.0135; E, *t*_26_ **=** 3.104, 2.425, and 2.456, **P = 0.0046, *P = 0.0226 and 0.0210, respectively). (F) Increased self-grooming time in home cage in *Dip2a* KO mice (18 mice per genotype, *t*_34_ **=** 2.508, *P = 0.0171). (G) In the marble burying test, *Dip2a* KO mice had increased digging time (WT = 14 mice, KO = 15 mice, *t*_27_ **=** 2.386, *P = 0.0243). (H) Exploration time of mice in chambers of the sociability trial (S1, the chamber for stranger 1; O, chamber with empty cage) (WT = 17 mice, *t*_32_ **=** 2.847, **P = 0.0076 for S1 versus O; KO = 16 mice, *t*_30_ **=** 2.982, **P = 0.0056). (I) Exploration time in the social novelty trial (S2, the chamber for stranger 2) (WT, *t*_32_ **=** 4.234, ***P = 0.0002 for S1 versus S2; KO, *t*_30_ **=** 0.8245, n.s. *P =* 0.4161). (J) One microliter AAV9 (titer > 10^12^ GC/mL) carrying CaMKII-GFP control or CaMKII-GFP-8KQ was injected into the cerebral cortex of 4-week-old mice and observed after 4 more weeks. Sites of injection: (1) anteroposterior (AP), 2.0 mm; mediolateral (ML), 0.5 mm; dorsoventral (DV), 1.3 mm; (2) AP, −1.0 mm; ML, 2.0 mm; DV, 0.8 mm. (K) The expression of cortactin-8KQ in cerebral cortex at 9 weeks old. (L) Cortactin 8KQ rescued the excessive self-grooming in *Dip2a* KO mice (WT + control = 11, KO + control = 9, KO + 8KQ = 8 mice; one-way ANOVA, *F*_2, 25_ = 5.283, *P* = 0.013; post hoc LSD, **P = 0.0038, n.s. *P =* 0.4001, related to WT + control). (M) A model illustrating the function of DIP2A in spine morphology. DIP2A interacted with cortactin and maintained ac-cortactin, which is essential for the mushroom-like morphology and synaptic transmission of dendritic spines. The underlying data for this figure can be found in S1 Data. AAV9, adeno-associated virus type 9; ac-cortactin, cortactin acetylation; AP, anteroposterior; CaMKII, Ca^2+^/calmodulin-dependent protein kinase II; *Dip2a*, disconnected-interacting protein homolog 2 A; DV, dorsoventral; GFP, green fluorescence protein; KO, knockout; LSD, least significant difference; ML, mediolateral; n.s., no significance; O, chamber with empty cage; S1, the chamber for stranger 1; USV, ultrasonic vocalization; WT, wild-type; 8KQ, acetylation mimetic cortactin with eight lysine replaced with glutamine.

Disrupted social communication is observed in patients with ASD. Thus, we subjected postnatal day 4 (P4) mice to an isolation-induced ultrasonic vocalization (USV) assay [44] (Fig 7A). Reduced USV call duration (31.63 ± 2.79 milliseconds and 22.02 ± 2.15 milliseconds for WT and *Dip2a* KO mice, respectively; Fig 7B) and calling time (31.50 ± 2.05 seconds and 24.07 ± 1.80 seconds for WT and KO, respectively; Fig 7C) were observed in *Dip2a* KO pups compared with their WT littermates during the 10-minute test. These results suggest the deletion of *Dip2a* impaired social communication in mice.

In the open field test, locomotor movements and activity were recorded and analyzed. We found *Dip2a* KO mice had increased self-grooming frequency (S8A Fig, *P* = 0.0535), which was defined as stroking or scratching of the face, head, or body with both forelimbs [45]. No significant difference was detected in rearing frequency (S8A Fig), moving distance (S8B Fig), or average speed (S8C Fig) in comparison with WT littermates. The total self-grooming time in *Dip2a* KO mice increased by approximately 30% in the open field assay (182.9 ± 15.10 seconds and 240.8 ± 15.79 seconds in total time for WT and KO, respectively; Fig 7D and 7E) and by approximately 38% in the home-cage assay (95.81 ± 7.98 seconds and 128.6 ± 10.36 seconds for WT and KO, respectively; Fig 7F) when compared with their WT littermates. In the marble burying test [46], the time spent digging was also significantly increased in *Dip2a* KO mice compared with their WT littermates (10.41 ± 1.39% and 17.07 ± 2.36% for WT and KO, respectively; Fig 7G). These results indicate that *Dip2a* KO mice displayed excessive stereotypical repetitive behaviors, which is characteristic of ASD.

Because the performance of social behaviors in rodents requires normal olfaction [47], we firstly excluded olfactory impairment with the buried food test (S8D Fig) in *Dip2a* KO mice. We then employed the three-chamber test to assess social interaction and social novelty with voluntary initiation in these mice [48]. Sociability was defined as the subject mouse preferring interaction with stranger 1 (S1) over the empty cage (object, O) (S8E Fig). We found both *Dip2a* KO mice and WT littermates spent more time in the chamber with S1 (Fig 7H and S8F Fig), indicating an equal predilection for S1. In the social novelty test, another unfamiliar mouse was introduced as a novel social partner (stranger 2, S2) into the aforementioned empty cage (S8G Fig). In this context, WT mice exhibited a preference for exploring the chamber with S2 (147.7 ± 14.81 seconds and 235.8 ± 14.61 seconds in the S1 chamber and the S2 chamber, respectively; Fig 7I and S8H Fig), while *Dip2a* KO mice displayed no significant preference (185.2 ± 19.8 seconds and 206.9 ± 17.34 seconds in the S1 chamber and the S2 chamber, respectively; Fig 7I and S8H Fig). These social responses indicate *Dip2a* KO mice had defects in social novelty but not in sociability. Overall, *Dip2a* KO mice exhibited typical autistic-like behaviors, including increased repetitive behavior and anxiety, and impaired social novelty and communication.

Our data have shown that DIP2A was localized in dendritic spines and regulated spine function via acetylation cortactin. To further explore the acetylation mimetic cortactin restorative effect on murine behaviors, an adeno-associated virus (AAV) cortactin-8KQ initiating under the CaMKII promoter was injected into murine cerebral cortex at 4 weeks of age (Fig 7J). At 9 weeks of age, cortactin 8KQ was well expressed in cerebral cortex (Fig 7K). And the behavioral tests showed that repetitive self-grooming in *Dip2a* KO mice was greatly rescued. The self-grooming time increased from 102.6 ± 14.41 seconds (WT + control) to 167.7 ± 18.16 seconds (KO + control) and was then restored to 120.7 ± 9.56 seconds (KO + 8KQ) in the home cage for 10 minutes (Fig 7L). However, we did not detect significant restorative effects on the impairment of preference in social novelty with this AAV-mediated protocol (S8I–S8L Fig).

Altogether, our data demonstrate DIP2A interacts with cortactin and maintains its acetylation. The acetylation status of cortactin influences the F-actin affinity of cortactin, which in turn impacts on spine morphology and synaptic transmission. These findings provide evidence that lacking DIP2A is associated with synaptic dysfunction and the core aspects of autism-like behaviors in mice (Fig 7M).

## Discussion

Cortactin is an important regulator of actin cytoskeleton in dendritic spine morphogenesis [30,49]. In the present study, we show that DIP2A, coded by the autism candidate gene *DIP2A*, interacts with cortactin, which is essential for DIP2A-mediated acetylation of cortactin. Our findings reveal a novel function of DIP2A in dendritic spine and highlight the contribution of synaptic protein acetylation in neurodevelopment.

### DIP2A-mediated acetylation of cortactin is necessary for spine morphology and function

Acetylated cortactin is present in dendritic spine heads and shafts. This distribution resembles a lock effect for maintaining the neck morphology in dendritic spines [34]. In turn, acetylated cortactin appears to facilitate PSD95 clustering in synapses [34,50]. Here, we have discovered DIP2A could specifically mediate ac-cortactin, accounting for the defective spine morphogenesis in *Dip2a* KO mice.

*Dip2a* deletion reduced acetylated cortactin in dendritic spine shafts and removed the holding effect for spine neck morphology, causing a morphological shift from mushroom-like to stubby spines and flattened PSD. The scaffolding of PSD is responsible for the stabilization, recruitment, and trafficking of NMDAR and AMPAR to the postsynaptic membrane. Deletion of *Dip2a* in mice is associated with behavioral abnormalities and decreased synaptic glutamate receptor levels, which have been implicated in autism. More importantly, acetylation mimetic cortactin rescued the defective synaptic transmission and excessive self-grooming behaviors seen in *Dip2a* KO mice.

The dynamics of posttranslational modification in synaptic protein composition is critical for synaptic plasticity [51]. Our results establish a specific relationship between hypo-acetylated cortactin and synaptic dysfunction. To support our study, prenatal exposure to valproic acid, a HDAC inhibitor, appears to increase the risk of ASD in offspring, emphasizing the association of epigenetics with ASD [52,53]. Recently, another HDAC inhibitor, romidepsin, has been shown to alleviate ASD-like behaviors in *Shank3*-mutant mice [54]. These results suggest imbalanced histone, as well as synaptic protein acetylation, is an important neurobiological mechanism in ASD.

### DIP2A may provide acetyl donor for acetylation of cortactin

We found the levels of Ac-CoA were down-regulated in *Dip2a* KO mice, confirming that DIP2A is involved in Ac-CoA synthesis [21]. Optimal protein acetylation requires sufficient levels of Ac-CoA as the sole acetyl group donor [38]. The lower levels of Ac-CoA we observed in *Dip2a* KO mice may account for the decreased ac-cortactin.

It is unclear why DIP2A regulates the acetylation of cortactin but not that of tubulin or histones. We speculate DIP2A synthesizes Ac-CoA in close proximity to cortactin, which in turn provides a local pool of the Ac-CoA required for ac-cortactin. Therefore, the DIP2A–cortactin interaction is important for ac-cortactin mediated by DIP2A. A similar mechanism has been reported in the mouse hippocampus, where Ac-CoA synthetase 2 mediates Ac-CoA synthesis in the nuclei, supporting the fast turnover of histone acetylation [55].

### DIP2A selectively targets the basal dendritic spine of cortical neurons

Variations in spine density and the morphology of cortical pyramidal neurons are commonly found in ASD mouse models [56,57]. In this study, we have revealed that the deletion of *Dip2a* specifically increased the basal dendritic spine density of pyramidal neurons in the cerebral cortex, without affecting apical parts. This region-specific alteration may result from the asymmetric dendrites of pyramidal neurons. Accordingly, dendritic subtypes (basal versus apical dendrites) are functionally specialized, and their morphogenesis relies on distinct cellular pathways [58–60]. For example, during brain development, CUX1 has a stronger effect on the development of basal processes, while CUX2, with a similar expression pattern, preferentially affects apical dendrite differentiation [61]. The thousand-and-one-amino acid 2 kinase (TAOK2), encoded by the ASD susceptibility gene *TAOK2*, selectively regulates the formation of basal dendrites through interacting with Neuropilin 1, the receptor of the secreted guidance cue Semaphorin 3A that controls basal dendrite arborization [62,63]. This indicates that a single gene can promote the integration of intracortical networks in highly specific downstream pathways.

In addition, the distinct morphologies of basal and apical dendrites suggest that inputs to these domains might be integrated differently [59]. NMDA spikes have been observed in basal dendrites but not apical dendrites, which suggests that the excitability of basal dendrites might be different from that of apical dendrites [64]. In *Dip2a* KO mice, the postsynaptic NMDAR level was more significantly decreased than the AMPAR level (Fig 2M). Furthermore, environmental enrichment is a paradigm to investigate the in vivo effects of neural activity on spine density and morphology, while exploratory behavior in a novel environment especially increased the basal dendritic spine density [65], and cortactin is an activity-dependent synaptic regulator [8]. Thus, we speculate that DIP2A-regulated spinogenesis may be a selective consequence of the input computation and presynaptic signals.

### DIP2A-deficient mice exhibited immature dendritic spines and autism-like behaviors

Genetic studies in humans have implicated an association of mutations in the *DIP2A* gene with neurodevelopmental disorders [15–20]. However, the neuronal consequence of mutations in this gene remains unclear. In this study, we have demonstrated *Dip2a* KO mice presented synaptic dysfunction and ASD-like behavioral impairments, which resemble the core symptoms of patients with ASD. Therefore, our findings strengthen the evidence suggesting that deletion of *Dip2a* leads to ASD-like phenotypes in mice.

Stubby spines are shorter and squatter than mushroom-like spines and are thus viewed as immature structures [66]. In our study, *Dip2a* KO mice exhibited a significantly higher ratio of stubby spines and flattened synapses. Based on previous studies [67,68], we assume that reduced amplitude of synaptic transmission derives from spine morphology shifting from mushroom-like to stubby shapes, where the latter does not have the large spine head area characteristic of mushroom-like spines. These findings establish a strong link between stubby spines and reduced EPSC amplitude with ASD-like behaviors in *Dip2a* KO mice. Considering the expression of DIP2A in embryonic and early postnatal days, we speculate that DIP2A also affects other processes of neuronal development, such as the function of the autism-associated gene *phosphatidylinositol 3*,*4*,*5-trisphosphate-dependent Rac exchanger 1* (*P-Rex1*) in synaptic transmission and neuronal migration [69,70].

ASD-related *DIP2A* mutations appear to be heterozygous in the clinical context. However, given the unknown physiological role of DIP2A in the mammalian brain and the underlying functional consequences of its disruption, we subjected *Dip2a* KO homozygous mice to various behavioral, electrophysiological, and biochemical analyses in order to elucidate the major functional significance of the DIP2A protein. Further studies will be needed to investigate the potential functional deficits resulting from only one of the alleles being disrupted in *Dip2a* heterozygous mice. Interestingly, the stop gained or frameshift mutations in ASD-associated *DIP2A* were detected in the AFD [15–17], which is essential for the supposed Ac-CoA synthesis function of DIP2A. Thus, it will be valuable to test the ASD-associated DIP2A de novo mutations reported.

In summary, our data provide a molecular mechanism linking genetic mutations of *DIP2A* to ASD, in which DIP2A modulates spine morphology and synaptic transmission by regulating acetylation of cortactin. These findings have uncovered regulating acetylation of histone and nonhistone proteins as novel targets for neurological therapeutics. It will help understand the intensive balance of protein acetylation in the process of neurodevelopment.

## Methods

### Ethics statement

All procedures were approved by Institutional Animal Care and Use Committee of Northeast Normal University (NENU/IACUC, AP20151009). National Standards of the People's Republic of China (GB/T 35892–2018), Laboratory Animal—Guideline for Ethical Review of Animal Welfare, was the guidance for our animal care and protocols.

### Mice

*Dip2a* KO mice were generated and maintained according to our previous report [13,22] and backcrossed with C57BL/6 over 9 generations to guarantee a stable genetic background. Heterozygous *Dip2a* KO mice were crossed for producing offspring. Littermate mice of WT and KO genotypes were allocated randomly for experiments.

### Protein extraction

Whole-cell or brain extracts were prepared with RIPA lysis buffer (50 mM Tris [pH 7.5], 150 mM NaCl, 1 mM EDTA [pH 8.0], 1 mM EGTA [pH 8.0], 1% NP40, 2.5 mM sodium pyrophosphate, 1 mM β-phosphoglycerol, 2 mM NaF, 1 mM Na_3_VO_4_, protease inhibitor cocktail [Roche, Indianapolis, IN]) with 1 mM PMSF added before use. For acetylated protein blotting, 2 μM TSA (Selleck Chemicals, Houston, TX) and 10 mM sodium butyrate (Selleck Chemicals, Houston, TX) were added into the lysis buffer before use. Synaptosome membrane and PSD fractions were prepared from the cortex of 8-week-old mice as described [69], detailed in S1 Text. Histone extracts from mouse cerebral cortex were prepared according to the protocol (Abcam, Cambridge, MA).

### Analysis of the morphology of dendritic spines

The coronal slices of the cerebral cortex (40-μm thick) were prepared and neurons were chosen randomly for imaging. The secondary or tertiary dendritic spines in cortical neurons were analyzed. Each image is a *z* series projection of approximately 10 to 15 images, averaged two times and taken at 0.2-μm depth intervals. Examples of spines were from neurons labeled with GFP as indicated in Fig 2A. The outline of the spine was manually traced (green line) from *z* series projection images such as these [71]. Spine length and head width were computed using Neurolucida 11 (MBF Bioscience, Williston, VT). The spine morphology was used to discriminate the spine types [25] (S1 Table).

### TEM

Mice were deeply anesthetized and transcardially perfused with heparinized normal saline, followed by 2.5% glutaraldehyde and 1% paraformaldehyde (PFA) in 0.1 M phosphate buffer (PB; pH 7.4). The cortex was removed and TEM analysis was performed as previously described [72]. Briefly, the cortex was removed from the whole brain, postfixed in the same fixative buffer for 2 h, and stored in PB overnight at 4 °C. Somatosensory barrel field cortex was cut transversely on a Vibratome at 70 μm. The pieces were osmicated with 0.5% OsO4 (in 0.1 M PB) for 1 h, dehydrated in graded alcohols, flat embedded in EMbed 812 (Electron Microscopy Sciences, Hatfield, PA), and cured for 48 h at 60 °C. Ultrathin 60-nm sections were cut and mounted on Formvar-coated single-slot grids. Sections were stained with uranyl acetate and lead citrate, then examined with an electron microscope (Hitachi H-7500; Hitachi, Tokyo, Japan). The width and thickness of the PSD cleft were analyzed with ImageJ 1.48 (https://imagej.nih.gov/). Three-dimensional TEM images were constructed from 30 serial sections using the Imaris 9.2 software (BitPlane, Zurich, Switzerland).

### Whole-cell recording from brain slices

The coronal slices of cerebral cortex (300-μm thick) preparation and whole-cell recording were performed as previously described [73]. Briefly, mice were deeply anesthetized with halothane and decapitated quickly. The whole brain was removed and placed in ice-cold artificial cerebrospinal fluid (ACSF) (140 mM NaCl, 3 mM KCl, 1.3 mM MgSO_4_, 1.4 NaH_2_PO_4_, 5 mM HEPES, 11 mM D-glucose, 2.4 mM CaCl_2_, 3.25 mM NaOH [pH 7.3, osmolarity adjusted to between 290 and 300 mOsm]) bubbled with 95% O_2_/5% CO_2_. The slices were incubated for 1 h in a chamber filled with 95%O_2_/5% CO_2_ equilibrated ACSF at room temperature (approximately 25°C) prior to recording. Patch pipettes were made with a puller (PB-10, Narishige, Tokyo, Japan) from thick-wall borosilicate glass (GD-1.5, Narishige, Tokyo, Japan) and were filled with a solution (120 mM potassium gluconate, 10 mM HEPES, 1 mM EGTA, 5 mM KCl, 3.5 mM MgCl_2_, 4 mM NaCl, 8 mM biocytin, 4 mM Na_2_ATP, and 0.2 mM Na_2_GTP [pH 7.3 with KOH, osmolarity adjusted to 300 mOsm]). Patch pipette resistances were 5–7 MΩ in the bath, with series resistances in the range of 10–20 MΩ. Membrane potentials and/or currents were monitored with an Axopatch 700B amplifier (Molecular Devices, Foster City, CA), filtered at 5 kHz and acquired through a Digidata 1440 series analog-to-digital interface on a personal computer using Clampex 10.4 software (Molecular Devices, Foster City, CA). EPSCs were analyzed with Mini Analysis 6.0.3 (Synaptosoft, Decatur, GA).

### Immunoprecipitation and pull-down

HEK293 cells transfected with assigned constructs or adult mice cortex were lysed in lysis buffer A (20 mM Tris [pH 8.0], 10 mM NaCl, 1 mM EDTA, 0.5% NP-40, 1 mM NaF, 1 mM Na_3_VO_4_, protease inhibitor cocktail [Roche, Indianapolis, IN], 1 mM PMSF). For immunoprecipitation, clarified lysates (1 mg) were incubated with appropriate antibodies overnight at 4 °C. Immune complexes were collected by ProteinA/G-Agarose (Roche, Indianapolis, IN) incubation. For pull-down assays, GST-cortactin variants and His-DIP2A_1–320_ were expressed in *Escherichia coli*. Cells were induced to protein overexpression under 0.1 mM isopropyl-β-D-thiogalactoside (IPTG) at 16 °C overnight. After sonication in lysis buffer (20 mM HEPES [pH 7.5], 120 mM NaCl, 10% glycerol, 2 mM EDTA, protease inhibitor cocktail [Roche, Indianapolis, IN], 1 mM PMSF), solubilized proteins were recovered by centrifugation and incubated with glutathione-sepharose (GE Healthcare, Freiburg, Germany) or Ni^2+^-NTA agarose (Qiagen, Hilden, Germany) in the presence of 3% Triton X-100 for 2 h at 4 °C. The proteins were washed several times with PBS. Equivalent amounts of each fusion protein (4 μg) were incubated with 1 mg cell or brain lysates overnight at 4 °C. For specific binding, glutathione-sepharose or Ni^2+^-NTA agarose was preincubated with the indicated lysate for 2 h at 4 °C prior to incubation with fusion protein.

### Quantification of acetyl-CoA

The concentration of acetyl-CoA in tissues or cells was measured using the PicoProbe acetyl-CoA assay kit (ab87546, Abcam, Cambridge, MA).

### In vitro acetylation assay

Bacterially expressed GST-tagged cortactin N terminus was purified by glutathione-Sepharose (GE Healthcare, Freiburg, Germany) and eluted by reduced glutathione (Sigma-Aldrich, St. Louis, MO). The acetylation reaction was performed with GST-cortactin and Ac-CoA in buffer containing 50 mM Tris (pH 8), 0.1 mM DTT, 10% glycerol, 10 mM sodium butyrate, and protease inhibitors for 1 h at 30 °C [39].

### Primary neuron culture and viral infection

Primary cortical neurons were isolated from P0 pups as previously described [74], in accordance with the guidelines of the National Institutes of Health, as approved by the Animal Care and Use Committee at the Northeast Normal University. Lentiviral particles containing expression vectors of cortactin 8KQ in an Ubi-MCS-3FLAG-SV40-EGFP-IRES-puromycin backbone, an adenovirus containing pAdEasy-DIP2A-His8-IRES-GFP, and corresponding mock vectors were purchased from GeneChem (Shanghai, China).

### Whole-cell recording from cultured neurons

Whole-cell patch-clamp recordings were performed in the voltage-clamp mode using an EPC-10/2 amplifier (HEKA, Lambrecht/Pfalz, Germany). The recording pipettes were pulled from borosilicate glass capillary tubes (Warner Instruments, Hamden, CT) and had a resistance of 3–5 MΩ; only whole-cell patches with series resistances <15 MΩ were used for recording, and the membrane potential was held at −70 mV. The pipette solution consisted of 130 mM K-gluconate, 1 mM EGTA, 5 mM Na-phosphocreatine, 2 mM Mg-ATP, 0.3 mM Na-GTP, 5 mM QX-314, and 10 mM HEPES, pH 7.3. The bath solution consisted of 25 mM HEPES, pH 7.3, 128 mM NaCl, 30 mM glucose, 5 mM KCl, 5 mM CaCl_2_, 1 mM MgCl_2_, plus 50 μM D-AP5 and 20 μM bicuculline. The D-AP5, bicuculline, and QX-314 were from TOCRIS Bioscience, and the other chemicals were from Sigma-Aldrich (St. Louis, MO). For mEPSC recordings, 1 μM tetrodotoxin (TOCRIS Bioscience, Bristol, United Kingdom) was added to the bath solution. For evoke-EPSC trains, presynaptic inputs were stimulated with a CBAEC75 concentric bipolar electrode (FHC, Bowdoinham, ME) that was placed near (between 100 and 120 μm from) the recording neuron.

### Behavioral assays

Littermates were housed in mixed-genotype cages and subjected to experiments. The animals were acclimatized to the testing room for at least 1 h before the test. Before each individual mouse’s trial, the testing apparatus was cleaned with 75% ethanol. A curtain surrounded the equipment to prevent interference and to create a uniform environment. After each test, the mice were temporarily put into a new, clean cage to avoid affecting the naive mice. The sequence of behavioral tests was shown in S4 Table. Two independent cohorts of littermates with appropriate age were carried out for behavioral assays. We performed these tests based on published methods as referred to in S1 Text. All data were recorded and analyzed using Ethovision XT 10 (Noldus, Wageningen, the Netherlands) with a blind manner to the genotype.

### Stereotactic injection of AAV vector

The neuron-specific AAV serotypes 9 vectors (GeneChem, Shanghai, China) expressing EGFP under the CaMKIIa promoter (CaMKIIa-MCS-hGH poly(A) signal) were used as a vector backbone. Viruses were injected into the cerebral cortex according to the mouse brain atlas [75] at the following coordinates: (1) anteroposterior (AP), 2.0 mm; mediolateral (ML), 0.5 mm; dorsoventral (DV), 1.3 mm; (2) AP, −1.0 mm; ML, 2.0 mm; DV, 0.8 mm. One microliter AAV (titer > 10^12^ GC/mL) was delivered by a 5-μL micropipette (Hamilton, Bonaduz, Switzerland) connected to automatic pressure for 5 minutes. After each injection, the pipette was left in the tissue for 2 minutes before slowly being withdrawn.

### Statistics

All data are presented as the mean ± SEM. For groups with a normal distribution, statistical significance was determined by a two-tailed *t* test or ANOVA test (n.s. *P* > 0.05, *P < 0.05, **P < 0.01, and ***P < 0.001). Statistical tests were performed using SPSS 19.0 (IBM, Armonk, NY). Exact values of *n*, statistical results, and significance are shown in S7 Table. Original images supporting all blots were shown in S1 raw images.

## Supporting information

S1 Text Supplementary materials and methods. (DOCX)

S2 Text Unambiguous acetylated lysine residues identified by LC-MS/MS. LC-MS/MS, liquid chromatography–tandem mass spectrometry.
(DOCX)

S1 Data Underlying numerical data and statistical analysis for figure panels 1F; 2B, C, E, F, G, H, J, K, M; 3F, G, I, J; 4F, H; 5A, C, E; 6C, D, F, G; 7B, C, D, E, F, G, H, I, L; S1C, D; S3A, B, D, E, H, I, K; S4C, D, E; S5D; S6G, H, J, K; S7C, D, F, H, I; and S8A, B, C, D, J, L. (XLSX)

S1 raw images Original images supporting all blot and gel results reported in Figs 1A, C; 2M; 3A, B, C, E, F, G, H; 4A, B, D; 5B, D; S1A, B, F; S3B; S4E; S5A, B, C, G, H; S6B, D, E. The loading order, experimental samples, and molecular weight markers were indicated. The lanes used in the final figure were marked with a red dotted box and the lanes not used marked with an “X” above.
(PDF)

S1 Table Spine classification. (DOCX)

S2 Table Proteins found from LC-MS/MS. LC-MS/MS, liquid chromatography–tandem mass spectrometry.
(DOCX)

S3 Table Peptides identified from cortactin by LC-MS/MS. LC-MS/MS, liquid chromatography–tandem mass spectrometry.
(DOCX)

S4 Table Sequence of behavioral tests and observed phenotypes of *Dip2a* KO mice for each test. *Dip2a*, disconnected-interacting protein homolog 2 A; KO, knockout.
(DOCX)

S5 Table Primers used in this study. (DOCX)

S6 Table Antibodies used in this study. (DOCX)

S7 Table Statistical analysis results. (DOCX)

S1 Movie **Three-dimensional reconstructions of postsynaptic structure (yellow) with PSD (orange) in WT mice using serial TEM sections.** PSD, postsynaptic density; TEM, transmission electron microscopy; WT, wild-type.
(MP4)

S2 Movie **Three-dimensional reconstructions of postsynaptic structure (yellow) with PSD (orange) in KO mice.** KO, knockout; PSD, postsynaptic density.
(MP4)

S1 Fig DIP2A is expressed highly in adult mice brain. (A) Purified anti-DIP2A antibody strongly recognized a single protein band at the predicted size (approximately 180 kDa) in western blotting of C57/BL6 brain homogenates, while pre-immune serum or antibody after antigen binding could not. (B) Western blotting showing the level of DIP2A in cerebral cortex from the embryonic stage to adult, with constant increase during postnatal development. (C and D) Real-time PCR analysis showing the increasing relative *Dip2a* mRNA levels in cerebral cortex (C) and cultured neurons (D). *Gapdh* was used as normalized control. (E) Flowchart for PSD protein extraction (detailed information descripted in S1 Text). (F) Subcellular fractions from PSD protein extraction were detected by immunoblotting. Total, total homogenate; S, supernatant; P, pellet; SV, crude synaptic vesicle fraction. The underlying data for this figure can be found in S1 Data. DIP2A, disconnected-interacting protein homolog 2 A; DIV, day in vitro; E, embryo day; *Gapdh*, *glyceraldehyde-3-phosphate dehydrogenase*; P, postnatal day; PSD, postsynaptic density.
(TIF)

S2 Fig DIP2A is mainly expressed in excitatory neurons of cerebral cortex. (A) LacZ reporter mice (*Dip2a*^*lacZ/+*^) were used to display the endogenous DIP2A-expressed cell type. CUX1 and CTIP2 were stained in cortical sections to label pyramidal neurons in external and internal layers, respectively. Staining of CTIP2 has been applied with pseudocolor for better display of colocalization. (B) CaMKII was stained in cortical sections of *Dip2a*^*lacZ/+*^ mice to label excitatory neurons. CaMKII, Ca^2+^/calmodulin-dependent protein kinase II; CTIP2, B cell leukemia/lymphoma 11B; CUX1, cut-like homeobox 1; DIP2A, disconnected-interacting protein homolog 2 A; LacZ, *Dip2a* β-galactosidase.
(TIF)

S3 Fig *Dip2a* KO mice exhibit no detectable abnormal brain lamination. (A) Relative *Dip2a* mRNA levels in WT and KO brains (P56, males, 3 mice per genotype; *t* test, ***P < 0.0001). (B) Western blot demonstrating the specificity of the antibody against DIP2A in the *Dip2a* KO mice (4 mice per genotype for each experiment, data from 3 independent experiments). No significant changes were detected in DIP2B or DIP2C expression in *Dip2a* KO mice, meaning no distinct compensation effect from *Dip2a* KO. (C) The gross body and brain morphology of WT and *Dip2a* KO littermates (P56, males). (D) Quantification of body (P56, males, WT = 10, KO = 7; *t*_15_ = 0.4353, n.s. *P =* 0.6699) and brain weight (P56, males, *n* = 6 mice per genotype; *t*_10_ = 0.0491, n.s. *P =* 0.9617). (E) Line graph showing no difference between the body weight gain of WT and *Dip2a* KO littermates over a 70-day period (M, male; F, female). (F and G) Nissl staining of adult brain slices (P56, males, 3 mice per genotype). The three dashed white boxes represent different regions of cortex; from top to bottom are the external, the medium, and the internal parts. (H and I) Quantitative data of the cell density of Nissl-stained sections of S3F and G Fig. Cell density was measured using a fixed rectangular matrix of 180 × 280 μm^2^. E, external; M, medium; I, internal (*n* = 3 mice per genotype, 2 slices were captured each mouse; I, *t*_11_ = 0.4603, *P* = 0.6543; *t*_11_ = 0.7115, *P* = 0.4916; *t*_10_ = 0.0491, *P* = 0.9620; J, *t*_10_ = 0.5516, *P* = 0.5933; *t*_11_ = 0.4905, *P* = 0.6334; *t*_10_ = 1.223, *P* = 0.2495). (J) Immunostaining images from cortical sections of WT and *Dip2a* KO animals (P56, males; *n* = 3 mice per genotype). Different layer-specific markers were used to label the lamellar cortex: CUX1, layer II-IV; CTIP2, layer V; FoxP2, layer VI. (K) Quantitative data obtained from immunostaining of cortical layers. The percentage of positive markers in DAPI staining for (J) was assessed (II/III, *t*_4_ = 1.6867, *P* = 0.1669; IV, *t*_4_ = 1.7234, *P* = 0.1599; V, *t*_4_ = 1.4007, *P* = 0.2339; VI, *t*_4_ = 2.0915, *P* = 0.1047). The underlying data for this figure can be found in S1 Data. CTIP2, B cell leukemia/lymphoma 11B; CUX1, cut-like homeobox 1; *Dip2a*, disconnected-interacting protein homolog 2 A; FoxP2, forkhead box p2; KO, knockout; n.s., no significance; WT, wild-type.
(TIF)

S4 Fig *Dip2a* KO mice display increased spine density. (A) Representative figure of GFP-labeled cerebral cortex and pyramidal neurons from *Dip2a* KO × *Thy1-GFP* offspring. EPN and IPN are distinguished by white dotted line. In the right schematic, apical (blue line) and basal dendrites (red line) of pyramidal neurons are represented by different colors. (B) Representative images of GFP-labeled apical dendrites spines. Only the secondary and third dendrites were captured. (C) Quantification of confocal pictures showing spine densities of apical dendrites (EPN, WT = 53 neurons, KO = 50 neurons; *t*_101_ = 0.6437, *P =* 0.5212; IPN, WT = 48 neurons, KO = 57 neurons; *t*_103_ = 1.2237, *P =* 0.2188). (D) Dendritic spine classification in apical dendrites of external and internal layer neurons. (E) Western blot showing protein levels of glutamate receptor subunits in total cortical lysates. The histograms showing quantified protein expression (normalized to β-actin) in *Dip2a* KO mice. The ratio in WT mice was set to 100% (P56, males; 4 mice per genotype in each experiment; data from 3 independent experiments; n.s. *P >* 0.05). The underlying data for this figure can be found in S1 Data. *Dip2a*, disconnected-interacting protein homolog 2 A; EPN, external layer of pyramidal neurons; GFP, green fluorescence protein; IPN, internal layer of pyramidal neurons; KO, knockout; n.s., no significance; *Thy1*, *thymocyte antigen 1*; WT, wild-type.
(TIF)

S5 Fig DIP2A interacts with cortactin via the PXXP motifs. (A) FLAG-DIP2A was overexpressed into cultured HEK293 cells and purified with anti-FLAG M2 Affinity Gel. All the positive lanes that isolated with SDS-PAGE were subjected to LC-MS/MS. (B) Western blot and histogram showing level of SHANK3 protein. (C and D) Western blot and histogram showing no significant difference in acetylated histones levels between WT and *Dip2a* KO cerebral cortex (P56, males; 4 mice per genotype for each experiment; data from 3 independent experiments). Quantified acetylated protein level is normalized to total histone expression. The ratio in WT mice was set to 100%. The *P* values determined by two-tailed *t* test are 0.3747, 0.9998, 0.7325, 0.1254, 0.5839, 0.5959, 0.8905, and 0.1166, respectively. (E) Diagram of DIP2A and cortactin constructs. (F) Input of HEK293 cell lysates transfected with HA-tagged cortactin and FLAG-tagged full-length or truncated DIP2A. (G) IP and IB showing the N terminus of DIP2A (aa 1–320) interacted with cortactin. HA-cortactin was co-IPed with anti-FLAG antibody from cell lysates that were co-transfected with HA-cortactin and FLAG-tagged full-length DIP2A or N terminus–truncated DIP2A_1–320_. No HA-cortactin band was detected in the IP complex from cell lysates co-transfected with mock IgG, or the DIP2A_321–914_, DIP2A_915–1,562_ constructs. All input loading consisted of 1% of the lysates for IP. (H) Truncated His-DIP2A_1–320_ were expressed in *E*. *coli* and purified using Ni-NTA gel. Pull-down assay was performed in murine cerebral cortex lysate to detect the endogenous interaction of DIP2A, cortactin, and SHANK3. Coomassie staining showed the loading of purified DIP2A_1–320_. The underlying data for this figure can be found in S1 Data. aa, amino acid; co-IP, co-immunoprecipitation; DIP2A, disconnected-interacting protein homolog 2 A; FLAG, FLAG tag with the sequence DYKDDDDK; HA, human influenza hemagglutinin tag (YPYDVPDYA-tag); His, hexahistidine tag; IB, immunoblot; IgG, immunoglobulin G; IP, immunoprecipitation; KO, knockout; LC-MS/MS, liquid chromatography–tandem mass spectrometry; Ni-NTA, nickel-nitrilotriacetic acid; PXXP, proline-rich motif, where X is any residue; SHANK3, SH3 and multiple ankyrin repeat domains 3; WT, wild-type.
(TIF)

S6 Fig DIP2A-mediated ac-cortactin. (A) Relative mRNA levels of the indicated lysine acetyltransferase and deacetylase in WT and KO brains (males, P56; 3 mice per genotype; two-tailed unpaired *t* test, *P >* 0.05). (B) Reaction products in acetylation assays in vitro were resolved by SDS-PAGE, and the acetylated cortactin (lower arrow) and acetylated PCAF (upper thicker arrow) were visualized by radioautography (lane 1). DIP2A did not directly acetylate cortactin (lane 3). Total amounts of protein were monitored by Coomassie blue staining. No sample was loaded in lane 2 to avoid mixing of samples in lanes 1 and 3. (C) Unambiguous acetylated lysine residues identified by LC-MS/MS. (D) Western blot showing successful expression each of HA-tagged WT and mutant cortactin constructs (8KQ and 8KR) in HEK293 cells by transient transfection. (E) F-actin binding ability of cortactin was modulated by its acetylation level. Ratios of gray value of cortactin strips in pellets and supernatants are 1.028, 0.781, and 1.594 for HA-cortactin (WT), acetylation-mimetic cortactin (8KQ), and non-acetylation-mimetic cortactin (8KR), respectively. (F) Representative whole-cell voltage clamp traces of mEPSC from cultured neurons held at −70 mV. WT neurons were infected with Lv-eGFP-control or Lv-eGFP cortactin 8KQ at DIV10 and traced at DIV15. Scatterplot graph showing the equivalent mEPSC amplitude (G) and frequency (H) (*n* = 12 and 15 neurons, respectively; data from 3 independent experiments; *t* test; n.s. *P* > 0.05). (I) Representative whole-cell voltage clamp traces of mEPSC from cultured neurons. Neurons were infected with Lv-eGFP-control or Lv-eGFP cortactin at DIV10 and traced at DIV15. Scatterplot graph showing the equivalent mEPSC amplitude (J) and frequency (K). (J, one-way ANOVA, ***P < 0.0001 compared with WT + control, *P = 0.039 compared with KO + control; K, one-way ANOVA, *P =* 0.313). The underlying data for this figure can be found in S1 Data. ac-cortactin, cortactin acetylation; *Atat1*, alpha tubulin acetyltransferase 1; *Crebbp*, CREB binding protein; DIP2A, disconnected-interacting protein homolog 2 A; DIV, day in vitro; eGFP, enhanced green fluorescent protein tag; *Elp3*, elongator acetyltransferase complex subunit 3; *Ep300*, E1A binding protein p300; HA, human influenza hemagglutinin tag (YPYDVPDYA-tag); *Hdac*, histone deacetylase; KO, knockout; LC-MS/MS, liquid chromatography–tandem mass spectrometry; Lv, lentivirus; mEPSC, miniature excitatory postsynaptic current; *Pcaf*, p300/CBP-associated factor; WT, wild-type; 8KQ, acetylation mimetic cortactin with eight lysine replaced with glutamine; 8KR, non-acetylation mimetic cortactin with eight lysine replaced with arginine.
(TIF)

S7 Fig DIP2A modulates the postsynaptic localization of PSD95. (A) Representative images of dendritic segments of cultured neurons infected with adenovirus vectors expressing eGFP-DIP2A or eGFP control. The insets to the right show an example of the quantitative analysis of PSD95 distribution along the 2-μm vertical white line, which proceeds from the base of the dendritic shaft (set as 0). The dotted line depicts the outline of PSD95. (B and C) Quantitative traces illustrating the calculated fluorescence intensity of PSD95 along the scanning path as shown in (A). (D) The ratio of PSD95-positive puncta to total dendritic (MAP2) area was lower in *Dip2a* KO neurons than in WT. DIP2A overexpression in KO neurons rescued the ratio to WT levels (*n* = 19, 22, 18 neurons, respectively; one-way ANOVA, *F*_2, 56_ = 4.327, *P =* 0.0179; post hoc LSD, *P = 0.0102, n.s. *P =* 0.7983, compared with WT + control). (E) Representative whole-cell voltage clamp traces of evoked EPSCs in response to stimulation (arrows) in cultured neurons (DIV15). (F) The amplitude of evoked EPSCs in *Dip2a* KO neurons (*n* = 32) was reduced as compared with WT neurons (*n* = 22) (*t*_52_ = 4.189, *P* = 0.0001). (G) Representative whole-cell voltage clamp traces of mEPSC from cultured neurons held at −70 mV (*n* = 20 neurons per genotype; data from 3 independent experiments). (H) Ad-eGFP-DIP2A infection of *Dip2a* KO neurons rescued the reduction in amplitude (*t*_38_ = 19.82, *P <* 0.0001). (I) The frequency of mEPSCs was not significantly different in *Dip2a* KO neurons expressing eGFP versus eGFP-DIP2A (*t*_33_ = 0.08226, n.s. *P =* 0.9349). The underlying data for this figure can be found in S1 Data. Ad, adenovirus; DIP2A, disconnected-interacting protein homolog 2 A; DIV, day in vitro; eGFP, enhanced green fluorescent protein tag; EPSC, excitatory postsynaptic current; KO, knockout; LSD, least significant difference; MAP2, microtubule-associated protein 2; mEPSC, miniature excitatory postsynaptic current; n.s., no significance; PSD95, postsynaptic density protein 95; WT, wild-type.
(TIF)

S8 Fig Acetylation mimetic cortactin rescues synaptic defects and repetitive behaviors. (A) Frequency of rearing and self-grooming behaviors in the open field test (14 mice per genotype; *t*_26_ = 0.1138, *P =* 0.9103; *t*_26_ = 2.023, *P =* 0.0535). Line graph showing similar locomotor distance (B) and average speed (C), indications of locomotor activity. (D) Quantification of the behavioral olfaction as assessed in the buried food test (14 mice per genotype; *t*_26_ = 0.0318, *P =* 0.9748). Illustration of the apparatus used for the sociability test (E) and social novelty test (G). Representative exploring heat map of WT and KO mice in sociability trial (F) and social novelty trial (H). (I) Heat maps of mice in three-chambered sociability. (J) Exploration time of mice in three-chambered sociability (WT + control = 11, KO + control = 9, KO + 8KQ = 8 mice; ***P = 0.0009, *P = 0.0192 and 0.0463, respectively). (K) Heat maps of mice in three-chambered social novelty test. (L) Exploration time of mice in three-chambered social novelty test (WT + control = 11 mice, KO + control = 9 mice, KO + 8KQ = 8 mice; ***P = 0.0001, n.s. *P* = 0.6224 and 0.2950, respectively). The underlying data for this figure can be found in S1 Data. KO, knockout; n.s., no significance; WT, wild-type; 8KQ, acetylation mimetic cortactin with eight lysine replaced with glutamine.
(TIF)

## Abbreviations

aa: amino acid
AAV: adeno-associated virus
Ac-CoA: acetylated coenzyme A
ac-cortactin: cortactin acetylation
ac-tub: tubulin acetylation
ACSF: artificial cerebrospinal fluid
AFD: adenylate-forming domain
AMPAR: α-amino-3-hydroxy-5-methyl-4-isoxazolepropionic acid receptor
AP: anteroposterior
ASD: autism spectrum disorder
CaMKII: Ca^2+^/calmodulin-dependent protein kinase II
CTIP2: B cell leukemia/lymphoma 11B
CUX1: cut-like homeobox 1
DIP2A: disconnected-interacting protein homolog 2 A
DIV: day in vitro
DMAP1: DNA methylation 1–associated protein 1
DV: dorsoventral
EPN: external layer of pyramidal neurons
GAPDH: glyceraldehyde-3-phosphate dehydrogenase
GFP: green fluorescence protein
GluR1: glutamate ionotropic receptor AMPA type subunit 1
GST: glutathione S-transferase tag
HDAC6: histone deacetylase 6
IPN: internal layer of pyramidal neurons
IPTG: isopropyl-β-D-thiogalactoside
KO: knockout
LacZ: *Dip2a*-*β-galactosidase*
LC-MS/MS: liquid chromatography–tandem mass spectrometry
LSD: least significant difference
Lv: lentivirus
MAP2: microtubule-associated protein 2
mEPSC: miniature excitatory postsynaptic current
ML: mediolateral
NMDAR: N-methyl-D-aspartate receptor
NR1: NMDAR1
NR2A: NMDAR2
NR2B: NMDAR2B
P: postnatal day
PB: phosphate buffer
PCAF: lysine acetyltransferase 2B
PFA: paraformaldehyde
P-Rex1: phosphatidylinositol 3,4,5-trisphosphate-dependent Rac exchanger 1
PSD: postsynaptic density
sEPSC: spontaneous excitatory postsynaptic current
SH3: Src homology 3
SHANK3: SH3 and multiple ankyrin repeat domains 3
S1: stranger 1
S2: stranger 2
TAOK2: thousand-and-one-amino acid 2 kinase
TAT: trans-activator of transcription
TEM: transmission electron microscopy
Thy1: thymocyte antigen 1
TSA: trichostatin A
USV: ultrasonic vocalization
WT: wild-type
3D: three-dimensional
