# Supplementary material for: Autism candidate gene DIP2A regulates spine morphogenesis via acetylation of cortactin
Source: PLoS Biol. 2019 Oct 10;17(10):e3000461. doi: 10.1371/journal.pbio.3000461 (PMC6786517; doi:10.1371/journal.pbio.3000461)
Supplement: S1 Text — (DOCX) [file pbio.3000461.s001.docx]

**S1 Text. Supplementary** **materials and methods**

**cDNA constructs**

PCR-amplified mouse *Dip2a* (NM_001081419.2) was cloned into the pShuttle-IRES-hrGFP-1 vector (Agilent Technologies). FLAG-tagged DIP2A was constructed on the p3*FLAG-CMV-10 vector by subcloning from pShuttle-IRES-hrGFP1-m*Dip2a*-His8. FLAG-DIP2A truncated plasmids were constructed by subcloning the template of FLAG-DIP2A. HA-tagged cortactin was constructed on the pKH3 vector by subcloning from pcDNA-FLAG-cortactin, gift from Jun Zhou [1]. GST-fusion constructs of DIP2A and cortactin were generated on the pGEX-2T-1 and pGEX-4X-1 vector, respectively. GST-DIP2A_261-320_ with PXXP motif mutation, HA-cortactin 8KQ plasmids were mutated by using the Quick Change Lightning Site-Directed Mutagenesis Kit (Agilent Technologies). All primers used to construct plasmids are shown in (S5 Table).

**Antibodies**

Anti-DIP2A rabbit polyclonal antibody was generated by immunizing rabbits with a fusion protein of DIP2A_14-320_. The primary antibodies used are shown in (S6 Table). For western blots, HRP conjugated secondary antibodies (Sigma-Aldrich) were used. For immunofluorescence, secondary antibodies labeled with Alexa Fluor 488, Alexa Fluor 546, and Alexa Fluor 633 (Invitrogen) were used.

**Reverse transcription quantitative real-time PCR (qRT-PCR)**

Total RNA samples were isolated from mouse brains (P56, males) using Tripure reagent (QIAGEN) according to the manufacturer’s protocol. First-strand cDNA synthesis was carried out with oligo-dTs and reverse transcriptase (TransGen Biotech). Three independent cDNA samples were amplified in triplicate by qRT-PCR using SYBR-Green (TaKaRa) on the PIKOREAL96 Real-Time PCR System (Thermo Fisher). Gene expression fold changes were calculated with the comparative CT method and normalized to *gapdh*. Primer pairs used are shown in (S5 Table).

**Subcellular fractionation**

Synaptosomal membrane and PSD fractions were prepared from the cortex of 8-week-old mice as described [2]. Briefly, the cortex from 4 mice were homogenized in 10 mL of homogenization buffer (4 mM HEPES [pH7.4], 1 mM EGTA, 0.32 M sucrose, protease inhibitor cocktail [Roche], and 1 mM PMSF) in a glass Teflon homogenizer. The homogenate was centrifuged at 1 000 *g* for 10 min. The supernatant (S1) was collected and centrifuged at 9 200 *g* for 15 min, and the resulting crude synaptosomal pellet (P2) was washed twice by resuspension in 24 mL of homogenization buffer and was centrifuged at 10 200 *g* for 15 min. The washed P2 pellet was lysed by osmotic shock with ice-cold distilled water, homogenized in a glass Teflon homogenizer, and centrifuged at 25 000 *g* for 20 min to yield a supernatant (a crude synaptic vesicle [SV] fraction; S3) and a pellet (the lysed synaptosomal membrane fraction, P3). The P3 fraction was resuspended in an appropriate volume of homogenization buffer, loaded onto a discontinuous sucrose density gradient (1.2 M sucrose, 0.8 M sucrose), and centrifuged at 150 000 *g* for 1 h. Thereafter the fraction was removed from the interface between 1.2 M sucrose and 0.8 M sucrose and was centrifuged at 150 000 *g* for 30 min. The resulting pellet (synaptosomal membrane) was incubated for 15 min in ice-cold 0.5% Triton X-100 and then was centrifuged at 32 000 *g* for 20 min to obtain the PSD pellet. The S3 fraction was centrifuged at 165 000 *g* for 2 h; the resulting pellet was the SV fraction containing SV proteins.

**Nissl staining**

Mice were anesthetized with an intraperitoneal injection of tribromoethanol and then perfused with ice-cold PBS followed by cold 4% paraformaldehyde (PFA). Brains were cryoprotected in 30% (w/v) sucrose for 72 h and embedded in optimal cutting temperature (OCT) compound for sectioning into 40-μm-thick coronal slices. The sections were stained in Nissl staining solution (Beyotime) at 37°C for 90 min, then quickly rinsed in distilled water to remove excess stain. Cell density was measured in a fixed rectangular matrix, 180*280 μm^2^ representing different layers or sides as described before [3].

**Mass spectrum analysis**

HEK293 cells transfected with FLAG-DIP2A expression plasmids were lysed 48h later with lysis buffer (20 mM Tris [pH 8.0], 10 mM NaCl, 1 mM EDTA, 0.5% NP-40, 1 mM NaF, 1 mM Na_3_VO_4_, protease inhibitor cocktail [Roche, Basel, Switzerland], 1 mM PMSF). FLAG-DIP2A fusion protein and its binding partners were immunoprecipitated using anti-FLAG M2 Affinity Gel (Sigma-Aldrich). The FLAG peptide-eluted material was resolved by 8% SDS-PAGE. The gel was stained with Coomassie Brilliant Blue R-250 Dye. Compared with the control group (HEK293 cells transfected with FLAG-tag plasmids), bands in the FLAG-DIP2A channel were excised and subjected to mass spectrometry (Applied Protein Technology, Shanghai, China). Briefly, gel pieces were destained, rehydrated and tryptic digested with 10 ng/μl trypsin resuspended in 50 mM NH_4_HCO_3_. The tryptic peptides were dissolved in 0.1% formic acid (solvent A), directly loaded onto a reversed-phase analytical column. The gradient was comprised of an increase from 6% to 23% solvent B (0.1% formic acid in 98% acetonitrile). The peptides were subjected to NSI source followed by tandem mass spectrometry (MS/MS) in Q Exactive^TM^ Plus (Thermo Finnigan, California, USA) coupled online to the UPLC. The resulting MS/MS data were processed using Proteome Discoverer 1.3. Tandem mass spectra were searched against UniProt database. Trypsin was specified as cleavage enzyme allowing up to 2 missing cleavages. Mass error was set to 10 ppm for precursor ions and 0.02 Da for fragment ions. Carbamidomethyl on Cys were specified as fixed modification and oxidation on Met and acetylation modification were specified as variable modifications. Peptide confidence was set at high, and peptide ion score was set > 20.

**Histone extraction**

Histone extracts from mouse cerebral cortex were prepared according to the protocol (Abcam) with modifications. Briefly, cerebral cortex punches (diameter: ~2 mm) from fresh brain per mouse were collected, and then washed twice with 500 μl ice-cold PBS (containing 5 mM Sodium Butyrate). The punches were responded in homogenization buffer (PBS containing 0.5% Triton X 100, 2 mM PMSF, 0.02% NaN_3_) and lysed on ice for 30 min with gentle stirring. Centrifuge at 2 000 rpm for 10 minutes at 4°C. Remove and discard the supernatant. The pellet was washed in homogenization buffer and the resuspended in 0.2N HCl to extract the histones over night at 4°C. Centrifuge samples at 2000rpm for 10 minutes at 4°C and store aliquots.

**F-actin co-sedimentation assay**

F-actin co-sedimentation assays were performed as previously described [4]. Cells were lysed by incubation with ice-cold G-buffer (2 mM Tris-HCl [pH 8], 0.2 mM ATP, 0.5 mM DTT, and 0.2 mM CaCl_2_) for 15 min, followed by homogenization in a tight-fitting Dounce homogenizer. Cell lysates were clarified by centrifugation at 10 000 *g* for 10 min.

**Isolation induced ultrasonic vocalizations (USV) in mouse pups**

Four-day-old mouse pups were isolated from their home cage and placed in a clean beaker with bedding. USV was recorded for 5 min using the ultrasonic recording model 116H (Avisoft Bioacoustics) and analyzed using Avisoft-SASLab Pro software (Avisoft Bioacoustics) as describe [5].

**Open ﬁeld**

Mice were placed individually in a random corner of the box (50 × 50 × 35 cm) facing the wall. The movements, indicating locomotor activity, were recorded with a video camera for 15 min, and were analysed by Ethovision XT 10 (Noldus). Time and frequency of self-grooming and rearing, indicating repetitive behavior, were quantified blind manually. Self-grooming behavior was defined as stroking or scratching of the face, head, or body with the two forelimbs or licking body parts. Rearing was defined as rearing on the hind legs in the corner or along the sidewalls and jumping with both hind legs off the ground simultaneously [6].

**Repetitive behaviors in home cage**

After 10 min of habituation, individual mice in their home cages with fresh bedding were observed for 10 min to measure the time spent on repetitive behaviors, including self-grooming and rearing.

**Isolation induced ultrasonic vocalizations (USV) in mouse pups**

Four-day-old mouse pups were isolated from their home cage and placed in a clean beaker with bedding. USV was recorded for 5 min using the ultrasonic recording model 116H (Avisoft Bioacoustics) and analyzed using Avisoft-SASLab Pro software (Avisoft Bioacoustics) as describe [5].

**Marble burying test**

These tests were performed based on published method [7]. A clean home cage was filled ~5 cm deep with bedding material that was evenly distributed into a flat surface across the whole cage. During the habituation phase, the mice were introduced to the cage without any marbles for 20 min. During the testing phase, ten glass marbles (1.2 cm in diameter, plain dark glass) were then spaced evenly in a 2 × 5 grid on the surface of the bedding. Digging buried-marbles of mice were recorded with a video camera for 10 min, and quantified blind manually.

**Buried food test for olfaction**

We first confirmed that *Dip2a* deficiency had no effect on olfaction using a buried food test [8]. Before the test, mice were placed on a calorie-restricted diet (1.5-2 g of food per day) for 2 days. A pellet (10 mg) of sweetened breakfast cereal was buried underneath (~1 cm) bedding in a home cage. The mouse was placed in the center of the arena, and the time it took to find the food pellet was measured.

**Three-chamber sociability and social novelty preference assay**

The three-chamber test was performed as described previously [9]. Briefly, following habituation in chambers for 10 min, stranger 1 mouse (S1, C57BL/6 male, P25) was placed in a wire cage located in the corner of one side chamber, and the empty wire cage in the opposite side chamber is named object (O). Subject interaction was recorded for 10 min. Following the first test, object was replaced with stranger 2 (S2, C57BL/6 male, P25) in cage. Exploration time was defined as any occasion when a mouse’s nose touched the cage or came within 2 cm of it.

**References for S1 Text.**

1. Shi X, Yao Y, Wang Y, Zhang Y, Huang Q, Zhou J, et al. Cep70 regulates microtubule stability by interacting with HDAC6*.* FEBS Lett*.* 2015; 589: 1771-1777.

2. Li J, Chai A, Wang L, Ma Y, Wu Z, Yu H, et al. Synaptic P-Rex1 signaling regulates hippocampal long-term depression and autism-like social behavior*.* Proc Natl Acad Sci*.* 2015; 112: E6964-E6972.

3. Pilati N, Barker M, Panteleimonitis S, Donga R, Hamann M. A rapid method combining Golgi and Nissl staining to study neuronal morphology and cytoarchitecture*.* J Histochem Cytochem*.* 2008; 56: 539-550.

4. Heier JA, Dickinson DJ, Kwiatkowski AV. Measuring protein binding to F-actin by co-sedimentation*.* J Vis Exp*.* 2017; e55613.

5. Wöhr M. Effect of social odor context on the emission of isolation-induced ultrasonic vocalizations in the BTBR T+tf/J mouse model for autism*.* Frontiers in Neuroscience*.* 2015; 9: 73.

6. Lewis S. Autism: grooming mice to model autism*.* Nat Rev Neurosci*.* 2011; 12: 248-249.

7. Thomas A, Burant A, Bui N, Graham D, Yuva-Paylor LA, Paylor R. Marble burying reflects a repetitive and perseverative behavior more than novelty-induced anxiety*.* Psychopharmacology (Berl)*.* 2009; 204: 361-373.

8. Guo W, Allan AM, Zong R, Zhang L, Johnson EB, Schaller EG, et al. Ablation of Fmrp in adult neural stem cells disrupts hippocampus-dependent learning*.* Nat Med*.* 2011; 17: 559-565.

9. Yang M, Silverman JL, Crawley JN. Automated three-chambered social approach task for mice*.* Curr Protoc Neurosci*.* 2011; Chapter 8: 8-26.
