# Supplementary material for: Autism candidate gene DIP2A regulates spine morphogenesis via acetylation of cortactin
Source: PLoS Biol. 2019 Oct 10;17(10):e3000461. doi: 10.1371/journal.pbio.3000461 (PMC6786517; doi:10.1371/journal.pbio.3000461)
Supplement: S2 Text — LC-MS/MS, liquid chromatography–tandem mass spectrometry. (DOCX) [file pbio.3000461.s002.docx]

**S2 Text. Unambiguous acetylated lysine residues identified by LS-MS/MS.**

Sequence: SAVGHEYQS**K**LSK, K107-Acetyl (42.01057 Da)

Charge: +2, Monoisotopic m/z: 738.37744 Da (-0.66 mmu/-0.89 ppm), MH+: 1475.74761 Da, RT: 12.40 min,

Identified with: Mascot (v1.27); IonScore: 57, Exp Value: 1.9E-006, Ions matched by search engine: 10/116

Fragment match tolerance used for search: 0.02 Da

Sequence: HASQ**K**DYSSGFGGK, K152-Acetyl (42.01057 Da)

Charge: +2, Monoisotopic m/z: 755.85052 Da (+0.81 mmu/+1.08 ppm), MH+: 1510.69377 Da, RT: 12.13 min,

Identified with: Mascot (v1.27); IonScore: 90, Exp Value: 9.6E-010, Ions matched by search engine: 11/144

Fragment match tolerance used for search: 0.02 Da

Sequence: VD**K**SAVGFDYQGK, K171-Acetyl (42.01057 Da)

Charge: +2, Monoisotopic m/z: 728.36041 Da (+1.03 mmu/+1.41 ppm), MH+: 1455.71355 Da, RT: 16.68 min,

Identified with: Mascot (v1.27); IonScore: 28, Exp Value: 1.4E-003, Ions matched by search engine: 8/136

Fragment match tolerance used for search: 0.02 Da

Sequence: SAVGFDYQG**K**TEK, K181-Acetyl (42.01057 Da)

Charge: +2, Monoisotopic m/z: 736.35638 Da (-0.46 mmu/-0.62 ppm), MH+: 1471.70549 Da, RT: 15.97 min,

Identified with: Mascot (v1.27); IonScore: 49, Exp Value: 1.4E-005, Ions matched by search engine: 7/116

Fragment match tolerance used for search: 0.02 Da

Sequence: DYS**K**GFGGK, K193-Acetyl (42.01057 Da)

Charge: +2, Monoisotopic m/z: 500.74017 Da (-0.2 mmu/-0.41 ppm), MH+: 1000.47307 Da, RT: 14.07 min,

Identified with: Mascot (v1.27); IonScore: 40, Exp Value: 9.1E-005, Ions matched by search engine: 7/84

Fragment match tolerance used for search: 0.02 Da

Sequence: GFGG**K**FGVQTDR, K235-Acetyl (42.01057 Da)

Charge: +2, Monoisotopic m/z: 655.82739 Da (-0.67 mmu/-1.02 ppm), MH+: 1310.64751 Da, RT: 18.25 min,

Identified with: Mascot (v1.27); IonScore: 55, Exp Value: 3.1E-006, Ions matched by search engine: 9/116

Fragment match tolerance used for search: 0.02 Da

Sequence: GFGG**K**YGVQK, K309-Acetyl (42.01057 Da)

Charge: +2, Monoisotopic m/z: 541.78540 Da (+0.28 mmu/+0.52 ppm), MH+: 1082.56352 Da, RT: 14.28 min,

Identified with: Mascot (v1.27); IonScore: 35, Exp Value: 3.0E-004, Ions matched by search engine: 8/92

Fragment match tolerance used for search: 0.02 Da

Sequence: YGVQ**K**DR, K314-Acetyl (42.01057 Da)

Charge: +2, Monoisotopic m/z: 454.23541 Da (+0.16 mmu/+0.34 ppm), MH+: 907.46355 Da, RT: 10.53 min,

Identified with: Mascot (v1.27); IonScore: 55, Exp Value: 3.4E-006, Ions matched by search engine: 6/60

Fragment match tolerance used for search: 0.02 Da
