# Supplementary material for: Autism candidate gene DIP2A regulates spine morphogenesis via acetylation of cortactin
Source: PLoS Biol. 2019 Oct 10;17(10):e3000461. doi: 10.1371/journal.pbio.3000461 (PMC6786517; doi:10.1371/journal.pbio.3000461)

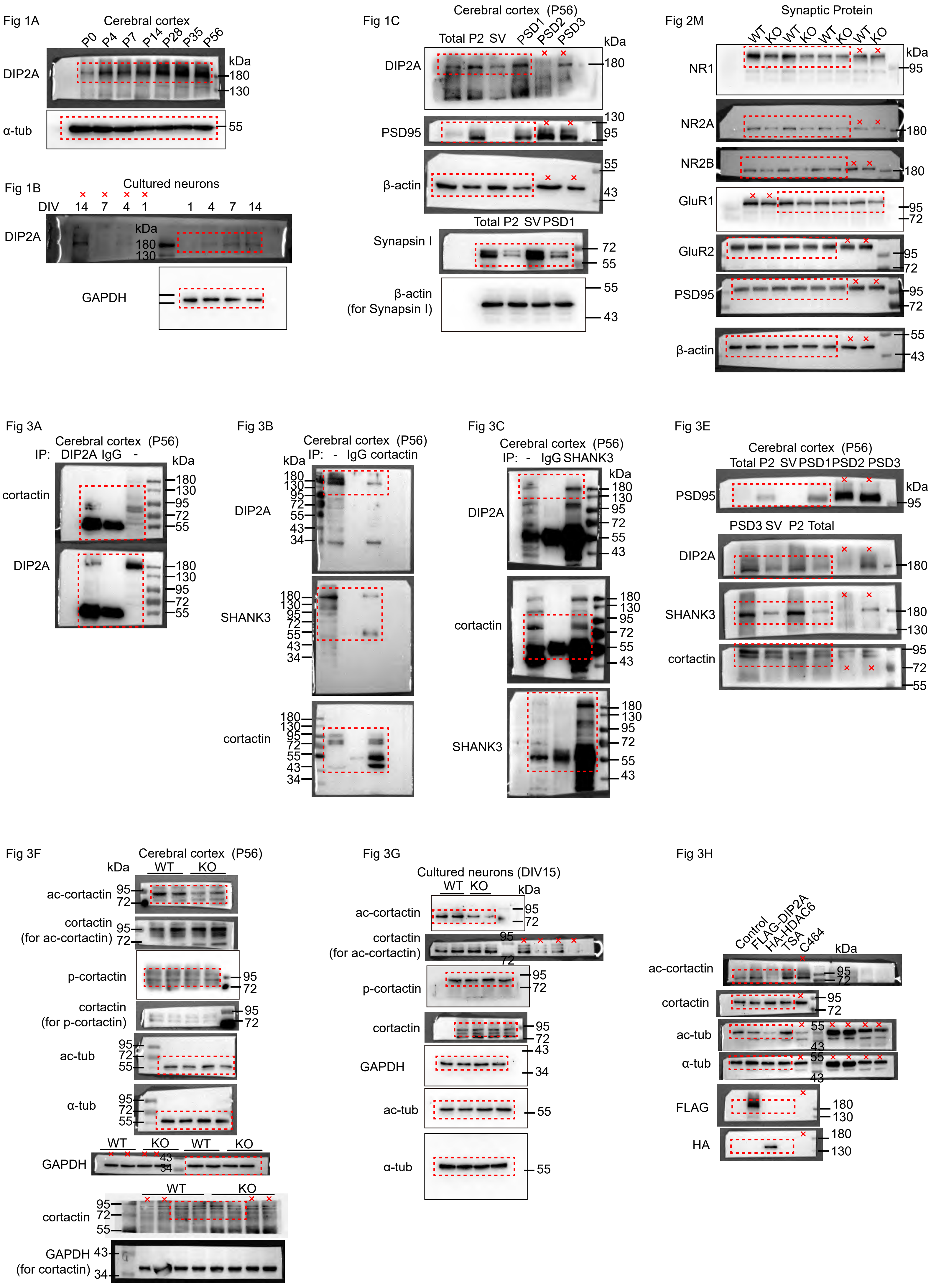
Fig 1A
Cerebral cortex
P0 P4 P7 P14 P28 P35 P56
DIP2A
α-tub
kDa
180
130
55
Fig 1B
Cultured neurons
DIV 14 7 4 1 1 4 7 14
DIP2A
kDa
180
130
GAPDH
Fig 1C
Cerebral cortex (P56)
Total P2 SV PSD1 PSD2 PSD3
kDa
DIP2A
180
PSD95
130
95
β-actin
55
43
Total P2 SV PSD1
Synapsin I
72
55
β-actin
(for Synapsin I)
55
43
Fig 2M
Synaptic Protein
WT KO WT KO WT KO WT KO
kDa
NR1
95
NR2A
180
NR2B
180
GluR1
95
72
GluR2
95
72
PSD95
95
72
β-actin
55
43
Fig 3A
Cerebral cortex (P56)
IP: DIP2A IgG -
kDa
cortactin
180
130
95
72
55
DIP2A
180
130
95
72
55
Fig 3B
Cerebral cortex (P56)
kDa
IP: - IgG cortactin
DIP2A
180
130
95
72
55
43
34
SHANK3
180
130
95
72
55
43
34
cortactin
180
130
95
72
55
43
34
Fig 3C
Cerebral cortex (P56)
IP: - IgG SHANK3
kDa
DIP2A
180
130
95
72
55
43
cortactin
180
130
95
72
55
43
SHANK3
180
130
95
72
55
43
Fig 3E
Cerebral cortex (P56)
Total P2 SV PSD1 PSD2 PSD3
PSD95
kDa
95
PSD3 SV P2 Total
DIP2A
180
SHANK3
180
130
cortactin
95
72
55
Fig 3F
Cerebral cortex (P56)
kDa
WT KO
ac-cortactin
95
72
cortactin
(for ac-cortactin)
95
72
p-cortactin
95
72
cortactin
(for p-cortactin)
95
72
ac-tub
95
72
55
α-tub
95
72
55
WT KO WT KO
GAPDH
43
34
WT KO
cortactin
95
72
55
GAPDH
(for cortactin)
43
34
Fig 3G
Cultured neurons (DIV15)
WT KO
kDa
ac-cortactin
95
72
cortactin
(for ac-cortactin)
95
72
p-cortactin
95
72
cortactin
95
72
GAPDH
43
34
ac-tub
55
α-tub
55
Fig 3H
Control
FLAG-DIP2A
HA-HDAC6
TSA
C464
kDa
ac-cortactin
95
72
cortactin
95
72
ac-tub
55
43
α-tub
55
43
FLAG
180
130
HA
180
130

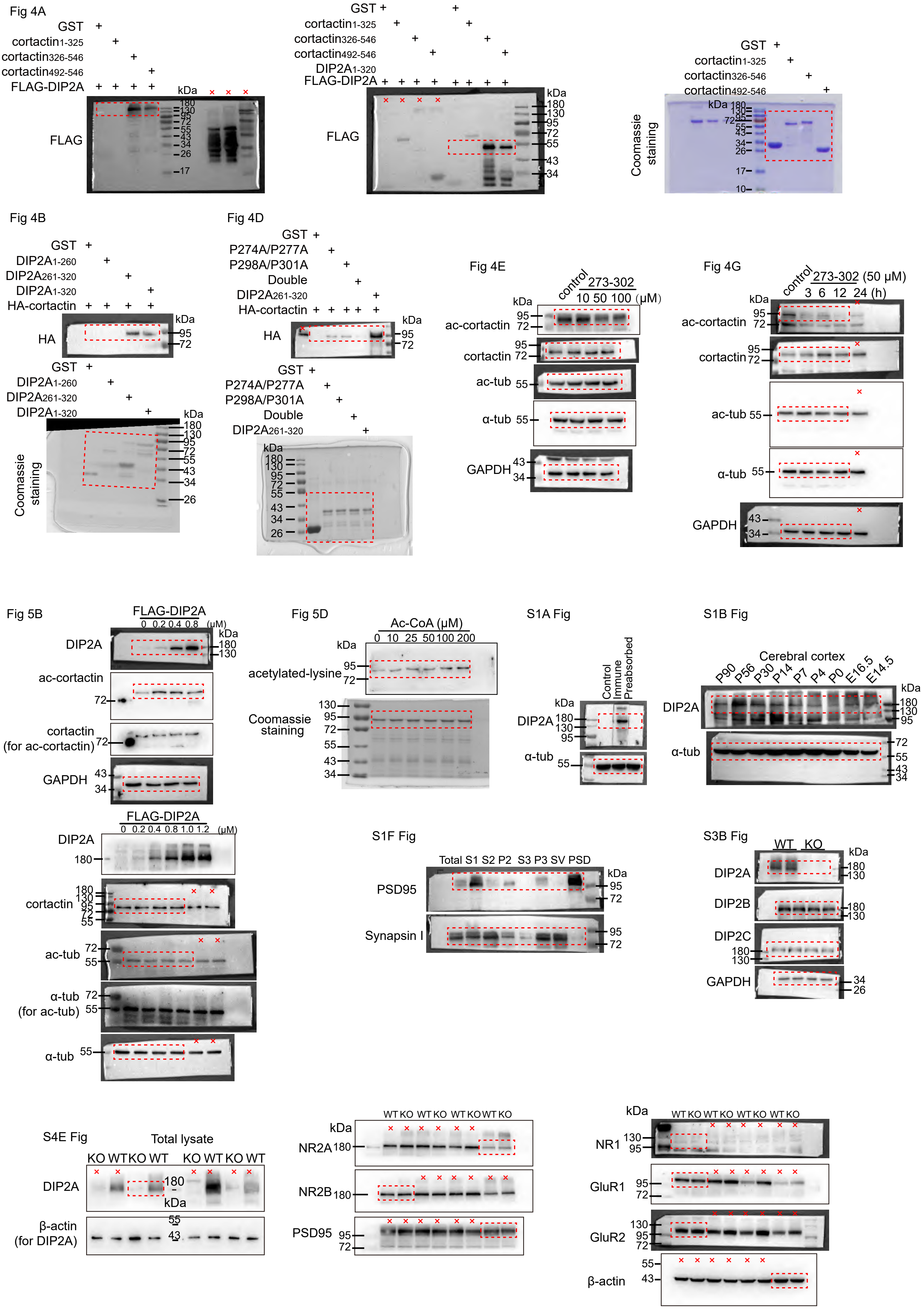

Fig 4A
GST
cortactin1-325
cortactin326-546
cortactin492-546
FLAG-DIP2A
kDa
FLAG
DIP2A1-320
Coomassie staining
Fig 4B
DIP2A1-260
DIP2A261-320
DIP2A1-320
HA-cortactin
HA
Fig 4D
P274A/P277A
P298A/P301A
Double
Fig 4E
control
273-302
10 50 100 (μM)
ac-cortactin
cortactin
ac-tub
α-tub
GAPDH
Fig 4G
273-302 (50 μM)
3 6 12 24 (h)
Fig 5B
FLAG-DIP2A
0 0.2 0.4 0.8 (μM)
DIP2A
cortactin (for ac-cortactin)
0 0.2 0.4 0.8 1.0 1.2 (μM)
α-tub (for ac-tub)
Fig 5D
Ac-CoA (μM)
0 10 25 50 100 200
acetylated-lysine
S1A Fig
Control
Immune
Preabsorbed
S1B Fig
Cerebral cortex
P90 P56 P30 P14 P7 P4 P0 E16.5 E14.5
S1F Fig
Total S1 S2 P2 S3 P3 SV PSD
PSD95
Synapsin I
S3B Fig
WT KO
DIP2B
DIP2C
S4E Fig
Total lysate
KO WT KO WT KO WT KO WT
β-actin (for DIP2A)
WT KO WT KO WT KO WT KO
NR2A
NR2B
NR1
GluR1
GluR2
β-actin

S5A Fig

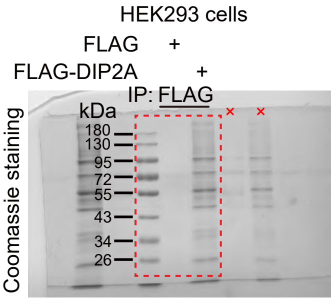

S5B Fig

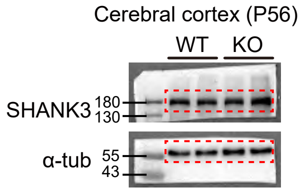

S5C Fig

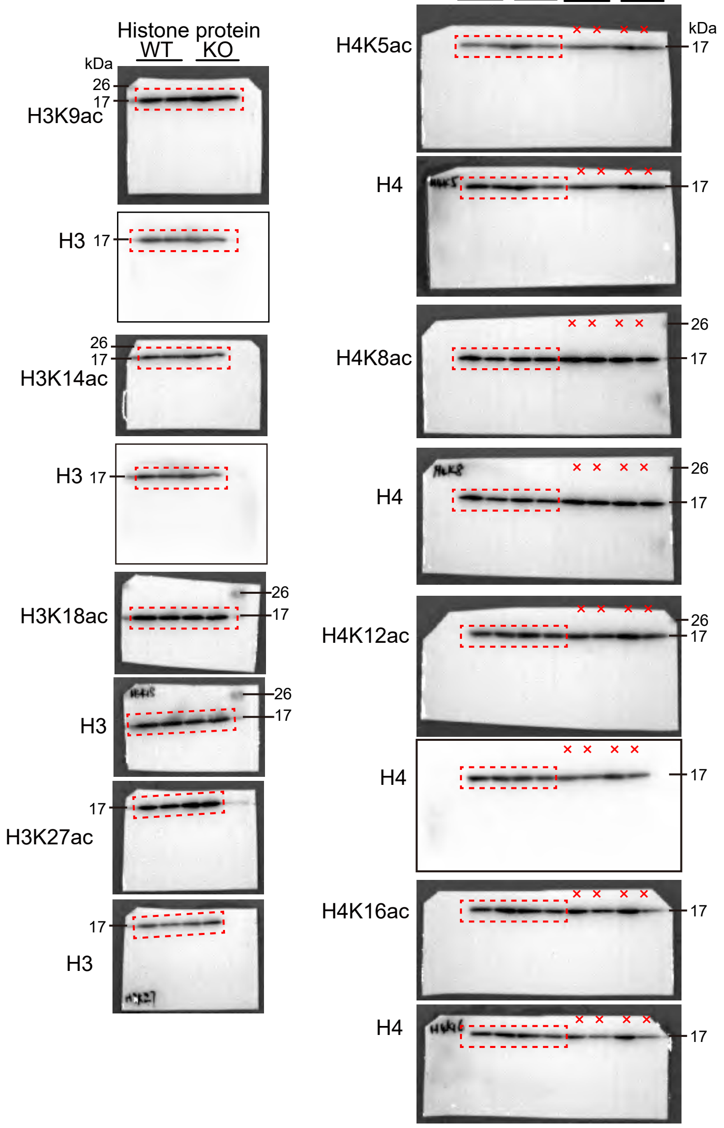

S5G Fig

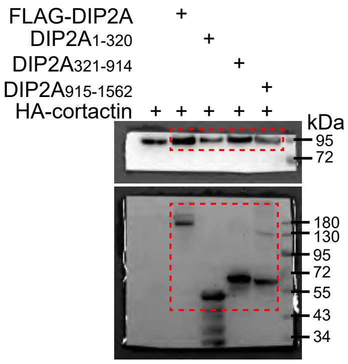

S5G Fig

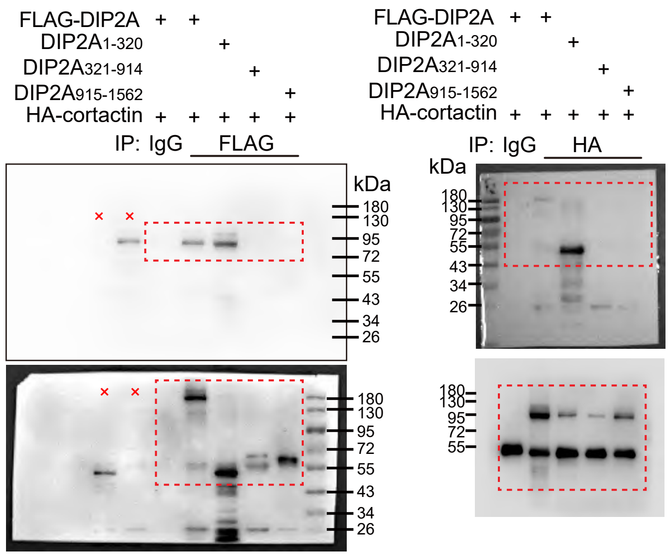

S5H Fig

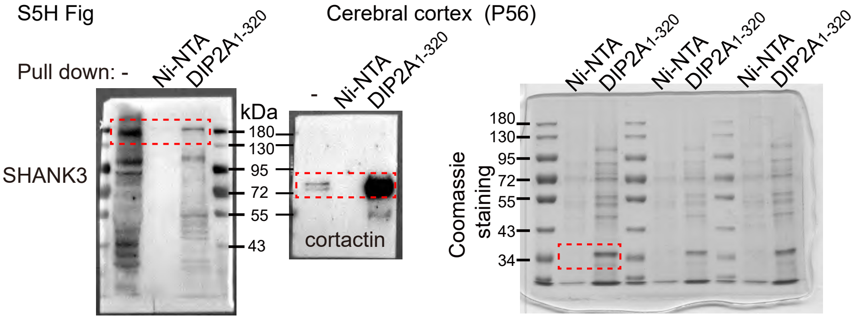

S6B Fig

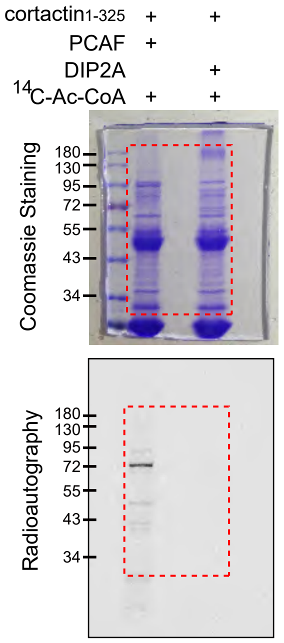

S6D Fig

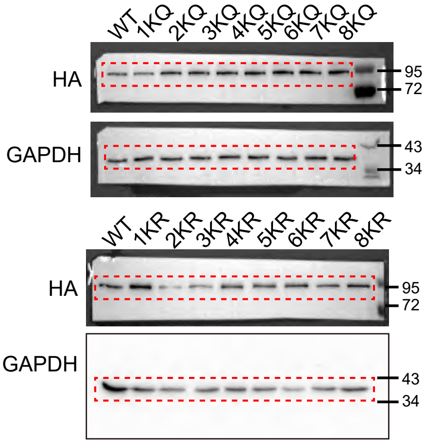

S6E Fig

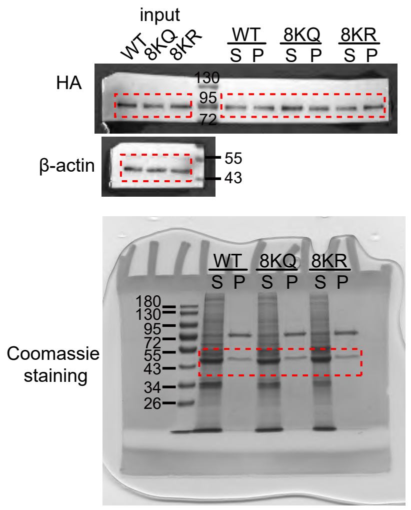

Supplement: S1 raw images — The loading order, experimental samples, and molecular weight markers were indicated. The lanes used in the final figure were marked with a red dotted box and the lanes not used marked with an “X” above. (PDF) [file pbio.3000461.s004.pdf]
