# Supplementary material for: Autism candidate gene DIP2A regulates spine morphogenesis via acetylation of cortactin
Source: PLoS Biol. 2019 Oct 10;17(10):e3000461. doi: 10.1371/journal.pbio.3000461 (PMC6786517; doi:10.1371/journal.pbio.3000461)
Supplement: S1 Table — (DOCX) [file pbio.3000461.s005.docx]

**S1 Table. Spine classification.**

|  | Length of spine (μm) | Width of head/width of neck |
| --- | --- | --- |
| Mushroom-like | <3 | >1.7 |
| Stubby | <1 | <1.7 |
| Thin | 1<L<3 | <1.7 |
| Branched | With two protrusions | |
