# Supplementary material for: Autism candidate gene DIP2A regulates spine morphogenesis via acetylation of cortactin
Source: PLoS Biol. 2019 Oct 10;17(10):e3000461. doi: 10.1371/journal.pbio.3000461 (PMC6786517; doi:10.1371/journal.pbio.3000461)
Supplement: S2 Table — LC-MS/MS, liquid chromatography–tandem mass spectrometry. (DOCX) [file pbio.3000461.s006.docx]

**S****2 Table. Proteins found from LC-MS/MS.**

| Gene ID^a^  (Mus) | Gene ID  (Homo) | Protein name | Gene name |
| --- | --- | --- | --- |
| 64451 | 23181 | Disco-interacting protein 2 homolog A | *Dip2a* |
| 15516 | 3326 | Heat shock protein HSP 90-beta | *Hsp90ab1* |
| 15519 | 3320 | Heat shock protein HSP 90-alpha | *Hsp90aa1* |
| 15525 | 3308 | Heat shock 70 kDa protein 4 | *Hspa4* |
| 15505 | 10808 | Heat shock protein 105 kDa | *Hsph1* |
| 14828 | 3309 | 78 kDa glucose-regulated protein | *Hspa5* |
| 22027 | 7184 | Endoplasmin | *Hsp90b1* |
| 269523 | 7415 | Transitional endoplasmic reticulum ATPase | *TERA/Vcp* |
| 13629 | 1938 | Elongation factor 2 | *Eef2* |
| 13627 | 1915 | Elongation factor 1-alpha 1 | *Eef1a1* |
| 17975 | 4691 | Nucleolin | *Ncl* |
| 16017 | 3500 | Ig gamma-1 chain C region secreted form (Fragment)/immunoglobulin heavy constant gamma 1 (G1m marker) | *Ighg1* |
| 109711 | 87 | Alpha-actinin-1 | *Actn1* |
| 11461 | 60 | Actin, cytoplasmic 1/beta actin | *Actb* |
| 56347 | 8663 | Eukaryotic translation initiation factor 3 subunit C | *Eif3c* |
| 27979 | 8662 | Eukaryotic translation initiation factor 3 subunit B | *Eif3b* |
| 13690 | 1982 | Eukaryotic translation initiation factor 4 gamma 2 | *Eif4g2* |
| 13669 | 8661 | Eukaryotic translation initiation factor 3 subunit A | *Eif3a* |
| 50995 | 10054 | Ubiquitin-like modifier-activating enzyme 1 | *Uba1* |
| 22202 | - | Ubiquitin-like modifier-activating enzyme 1 Y | *Uba1y* |
| 110750 | 1434 | Exportin-2 /chromosome segregation 1 like | *Cse1l* |
| 54624 | 54623 | RNA polymerase II-associated factor 1 homolog/RNA polymerase II complex component | *Paf1* |
| 56463 | 27044 | Staphylococcal nuclease domain-containing protein 1 | *Snd1* |
| 16211 | 3837 | Importin subunit beta-1/karyopherin subunit beta 1 | *Kpnb1* |
| 18571 | 10015 | Programmed cell death 6-interacting protein | *Pdcd6ip* |
| 104721 | 1653 | ATP-dependent RNA helicase DDX1, DEAD/H-box helicase 1, DEAD (Asp-Glu-Ala-Asp) box polypeptide 1 | *Ddx1* |
| 67040 | 10521 | Probable ATP-dependent RNA helicase DDX17, DEAD-box helicase 17, DEAD (Asp-Glu-Ala-Asp) box polypeptide 17 | *Ddx17* |
| 56200 | 9188 | Nucleolar RNA helicase 2 | *Ddx21* |
| 74351 | 9416 | MCG18410, isoform CRA_a | *Ddx23* |
| 228889 | 55661 | Probable ATP-dependent RNA helicase DDX27 | *Ddx27* |
| 212880 | 9879 | Probable ATP-dependent RNA helicase DDX46 | *Ddx46* |
| 71770 | 163 | AP-2(adaptor related protein complex 2) complex subunit beta | *Ap2b1* |
| 11765 | 164 | AP-1 complex subunit gamma-1 | *Ap1g1* |
| 101943 | 23450 | Splicing factor 3B subunit 3 | *Sf3b3* |
| 81898 | 23451 | Splicing factor 3B subunit 1 | *Sf3b1* |
| 75062 | 10946 | Splicing factor 3A subunit 1 | *Sf3a1* |
| 71514 | 6421 | Splicing factor, proline-and glutamine-rich，polypyrimidine tract binding protein associated | *Sfpq* |
| 20833 | 6749 | FACT complex subunit SSRP1，structure specific recognition protein 1 | *Ssrp1* |
| 21762 | 5708 | 26S proteasome non-ATPase regulatory subunit 2, proteasome (prosome, macropain) 26S subunit, non-ATPase, 2 | *Psmd2* |
| 70247 | 5707 | 26S proteasome non-ATPase regulatory subunit 1, proteasome (prosome, macropain) 26S subunit, non-ATPase, 1 | *Psmd1* |
| 13211 | 1660 | ATP-dependent RNA helicase A, DEAH-box helicase 9, DEAH (Asp-Glu-Ala-His) box polypeptide 9 | *Dhx9* |
| 72162 | 170506 | ATP-dependent RNA helicase DHX36 | *Dhx36* |
| 13204 | 1665 | Pre-mRNA-splicing factor ATP-dependent RNA helicase DHX15 | *Dhx15* |
| 106794 | 90957 | Putative ATP-dependent RNA helicase DHX57 | *Dhx57* |
| 53872 | 4076 | Caprin-1, cell cycle associated protein 1 | *Caprin1* |
| 11657 | 213 | Serum albumin | *Alb* |
| 28114 | 54888 | tRNA (cytosine(34)-C(5))-methyltransferase, NOP2/Sun RNA methyltransferase family member 2 | *Nsun2* |
| 14936 | 2997 | Glycogen [starch] synthase, muscle | *Gys1* |
| 11545 | 142 | Poly [ADP-ribose] polymerase 1 | *Parp1* |
| 17215 | 4172 | DNA replication licensing factor MCM3, minichromosome maintenance complex component 3 | *Mcm3* |
| 17217 | 4173 | DNA replication licensing factor MCM4 | *Mcm4* |
| 17218 | 4174 | DNA replication licensing factor MCM5 | *Mcm5* |
| 235497 | 123169 | RNA polymerase-associated protein LEO1, Paf1/RNA polymerase II complex component | *Leo1* |
| 67300 | 1213 | Clathrin heavy chain 1 | *Cltc* |
| 270685 | 25902 | Monofunctional C1-tetrahydrofolate synthase, mitochondrial methylenetetrahydrofolate dehydrogenase (NADP + dependent) 1-like | *Mthfd1l* |
| 108156 | 4522 | C-1-tetrahydrofolate synthase, cytoplasmic | *Mthfd1* |
| 51810 | 3192 | Heterogeneous nuclear ribonucleoprotein U (scaffold attachment factor A) | *Hnrnpu* |
| 16201 | 3609 | Interleukin enhancer-binding factor 3 | *Ilf3* |
| 243469 | 50802 | Ig kappa chain C region | *Igk* |
| 16572 | 3798 | Kinesin heavy chain isoform 5A | *Kif5a* |
| 70572 | 3843 | Importin-5 | *Ipo5* |
| 320727 | 10526 | Importin-8 | *Ipo8* |
| 78618 | 23527 | Arf-GAP with coiled-coil, ANK repeat and PH domain-containing protein 2 | *Acap2* |
| 19684 | 5962 | Radixin | *Rdx* |
| 114228 | 5644 | protease, serine 1 | *Prss1* |
| 19155 | 9520 | Puromycin-sensitive aminopeptidase | *Npepps* |
| 56041 | 8615 | General vesicular transport factor p115, USO1 vesicle transport factor，USO1 vesicle docking factor | *Uso1* |
| 26554 | 8452 | Cullin-3 | *Cul3* |
| 70767 | 9129 | U4/U6 small nuclear ribonucleoprotein Prp3, pre-mRNA processing factor 3 | *Prpf3* |
| 68879 | 24148 | Pre-mRNA-processing factor 6 | *Prpf6* |
| 383341 | - | MCG1031578, predicted pseudogene 5239 | *Gm5239* |
| 54194 | 26993 | A-kinase anchor protein 8-like | *Akap8l* |
| 223723 | 23170 | Tubulin--tyrosine ligase-like protein 12 | *Ttll12* |
| 71701 | 87178 | Polyribonucleotide nucleotidyltransferase 1 mitochondrial | *Pnpt1* |
| 103573 | 7514 | Exportin-1 | *Xpo1* |
| 110109 | 4839 | Probable 28S rRNA (cytosine-C(5))-methyltransferase, rRNA(C5-)-NOP2 nucleolar protein | *Nop2* |
| 14450 | 2618 | Trifunctional purine biosynthetic protein adenosine-3, phosphoribosylglycinamide formyltransferase, phosphoribosylglycinamide synthetase, phosphoribosylaminoimidazole synthetase | *Gart* |
| 50797 | 9276 | Coatomer subunit beta, coatomer protein complex subunit beta 2 | *Copb2* |
| 54161 | 22820 | Coatomer subunit gamma-1 | *Copg1* |
| 305851 | 11198 | FACT complex subunit SPT16,SPT16 homolog, facilitates chromatin remodeling subunit | *Supt16h* |
| 19024 | 8495 | Liprin-beta-2,PTPRF interacting protein, binding protein 2 | *Ppfibp2* |
| 109241 | 55777 | Methyl-CpG-binding domain protein 5 | *Mbd5* |
| 17685 | 4436 | DNA mismatch repair protein Msh2,mutS homolog 2 | *Msh2* |
| 14030 | 2130 | RNA-binding protein EWS,EWS RNA binding protein 1,Ewing sarcoma breakpoint region 1 | *Ewsr1* |
| 109620 | 1832 | Desmoplakin | *Dsp* |
| 12388 | 1500 | Catenin delta-1,catenin (cadherin associated protein), delta 1 | *Ctnnd1* |
| 20741 | 6710 | Spectrinxuey beta chain, erythrocytic | *Sptb* |
| 65114 | 55737 | Vacuolar protein sorting-associated protein 35，VPS35 retromer complex component | *Vps35* |
| 320528 | 54832 | Vacuolar protein sorting-associated protein 13C | *Vps13c* |
| 71902 | 55832 | Cullin-associated NEDD8-dissociated protein 1，cullin associated and neddylation disassociated 1 | *Cand1* |
| 67973 | 10199 | U3 small nucleolar ribonucleoprotein protein MPP10，M-phase phosphoprotein 10 | *Mphosph10* |
| 216443 | 4141 | Methionine--tRNA ligase, cytoplasmic tRNA | *Mars* |
| 14852 | 2935 | Eukaryotic peptide chain release factor GTP-binding subunit ERF3A, G1 to S phase transition 1 | *Gspt1* |
| 20133 | 6240 | Ribonucleoside-diphosphate reductase large subunit | *Rrm1* |
| 22350 | 7430 | Ezrin | *Ezr* |
| 224742 | 23 | ATP-binding cassette sub-family F member 1 | *Abcf1* |
| 21849 | 10155 | Transcription intermediary factor 1-beta，tripartite motif containing 28 | *Trim28* |
| 56320 | 1627 | Drebrin | *Dbn1* |
| 243880 |  | NACHT, LRR and PYD/pyrin domains-containing protein 4A | *Nlrp4a* |
| 71472 | 10869 | Ubiquitin carboxyl-terminal hydrolase 19, ubiquitin specific peptidase 19 | *Usp19* |
| 360198 | 8350 | Histone H3.1,histone cluster 1, H3a | *Hist1h3a* |
| 26408 | 4217 | Mitogen-activated protein kinase kinase kinase 5 | *Map3k5* |
| 234734 | 16 | Alanine-tRNA ligase, cytoplasmic,alanyl-tRNA synthetase | *Aars* |
| 216345 | 196441 | Putative uncharacterized protein | *Zfc3h1* |
| 12263 | 717 | Complement 2 | *C2* |
| 20815 | 6732 | SRSF protein kinase 1, serine/arginine-rich protein specific kinase 1 | *Srpk1* |
| 224727 | 7917 | Large proline-rich protein BAG6，BCL2 associated athanogene 6 | *Bag6* |
| 338362 | 10090 | Uronyl 2-sulfotransferase | *Ust* |
| 233033 | 55095 | Serine/threonine-protein kinase PAK 4，sterile alpha motif domain containing 4B | *Samd4b* |
| 381983 | 114783 | Serine/threonine-protein kinase LMTK3，lemur tyrosine kinase 3 | *Lmtk3* |
| 110960 | 6897 | Threonine-tRNA ligase, cytoplasmic | *Tars* |
| 11928 | 476 | Sodium/potassium-transporting ATPase subunit alpha-1, ATPase Na+/K+ transporting subunit alpha 1 | *Atp1a1* |
| 228482 | 9824 | Rho GTPase-activating protein 11A | *Arhgap11a* |
| 104112 | 47 | ATP-citrate synthase，ATP citrate lyase | *Acly* |
| 19704 | 5976 | Regulator of nonsense transcripts 1, RNA helicase and ATPase | *Upf1* |
| 13043 | 2017 | Src substrate cortactin | *Cttn* |
| 11428 | 48 | Cytoplasmic aconitate hydratase, aconitase 1 | *Aco1* |

a. Database of genes from NCBI RefSeq genomes
