# Supplementary material for: Autism candidate gene DIP2A regulates spine morphogenesis via acetylation of cortactin
Source: PLoS Biol. 2019 Oct 10;17(10):e3000461. doi: 10.1371/journal.pbio.3000461 (PMC6786517; doi:10.1371/journal.pbio.3000461)
Supplement: S3 Table — LC-MS/MS, liquid chromatography–tandem mass spectrometry. (DOCX) [file pbio.3000461.s007.docx]

**S3 Table. Peptides identified from cortactin by LC-MS/MS.**

| Cytoprotein lysate | Peptides count | Peptides identified |
| --- | --- | --- |
| HEK293 cells | 1 | R.YGLFPANYVELR.Q |
| Cerebral cortex | 16 | K.ERQEQEEAR.R  K.FGVEQDR.M  K.FGVQMDR.V  K.FGVQMDR.V  K.FGVQSER.Q  K.FGVQTDR.Q  K.HCSQVDSVR.G  K.LQLHESQK.D  K.SAVGFDYQGK.T  K.SAVGFEYQGK.T  K.TVQGSGHQEHINIHK.L  K.YGIDKDKVDK.S  K.YGVQADR.V  K.TVPIEAVTSK.T  R.ANFENLAK.E  R.SAVGHEYQSK.L |
