# Supplementary material for: Autism candidate gene DIP2A regulates spine morphogenesis via acetylation of cortactin
Source: PLoS Biol. 2019 Oct 10;17(10):e3000461. doi: 10.1371/journal.pbio.3000461 (PMC6786517; doi:10.1371/journal.pbio.3000461)
Supplement: S4 Table — Dip2a, disconnected-interacting protein homolog 2 A; KO, knockout. (DOCX) [file pbio.3000461.s008.docx]

**S4 Table****. Sequence of behavioral tests and observed phenotypes of *Dip2a* KO mice for each test.**

| Age of mice | Order of test | Name of test | | Observed phenotype |
| --- | --- | --- | --- | --- |
| 4 days | 1 | Isolation-induced USV assay | | Impaired communication |
| 6~8 weeks | 1 | Open field | Locomotor activity | Normal |
|  |  |  | Repetitive behaviors | Hyperactivity |
|  | 2 | Repetitive behaviors in home cage | | Hyperactivity |
|  | 3 | Marble burying test | | Hyperactivity |
|  | 4 | Buried food test for olfaction | | Normal |
|  | 5 | Novel object recognition | | Normal |
|  | 6 | Three-chambered social test | | Impaired social interaction |
