# Supplementary material for: Autism candidate gene DIP2A regulates spine morphogenesis via acetylation of cortactin
Source: PLoS Biol. 2019 Oct 10;17(10):e3000461. doi: 10.1371/journal.pbio.3000461 (PMC6786517; doi:10.1371/journal.pbio.3000461)
Supplement: S5 Table — (DOCX) [file pbio.3000461.s009.docx]

| **S5 Table. Primers used in this study.** | | |
| --- | --- | --- |
| Content | Forward (5′ - 3′) | Reverse (5′ - 3′) |
| pShuttle -hrGFP1-m*Dip2a*-His8 | Fragment 1:  gcggccgccaccatggccgaccgcggatgccct | Fragment 1:  gtcgactactgtgctgccttctttgctgg |
|  | Fragment 2:  gtgattgatgggaagcatctgacg | Fragment 2:  gtgaggggtacagctcacactga |
|  | Fragment 3:  cgacagggatcttagctggagtt | Fragment 3:  gtcgacttagtggtgatggtgatggtgatgatgggcgctgcccatattgtaggccacgt |
| p3*FLAG-CMV-DIP2A | ataagaatgcggccgcggccgaccgcggatgccctc | gctctagatcacatattgtaggccacgtag |
| p3*FLAG-CMV-DIP2A 1-320 | ataagaatgcggccgcggccgaccgcggatgccctc | gctctagaagtccccactgacagaggctctc |
| p3*FLAG-CMV-DIP2A 321-914 | ataagaatgcggccgctaatgggccactgtctctgttgg | gctctagagtggagcgtcccttccagaaagcgc |
| p3*FLAG-CMV-DIP2A 915-1562 | ataagaatgcggccgcgctgcagtggcgggctcacacc | gctctagatcacatattgtaggccacgtag |
| pKH3-HA-cortactin | cgggatcctggaaagcttcagcaggcc | cggaattcctactgccgcagctccacatag |
| pGEX-2T-cortactin 1-325 | cgggatcctggaaagcttcagcaggc | cggaattcctaatcctcaaaggttgacgcattc |
| pGEX-2T-cortactin 326-546 | cgggatcctgggaaagcttcagcaggc | cggaattcctaatcctcaaaggttgacgcattc |
| pGEX-2T-cortactin 492-546 | cgggatccatcacagccgtcgccctg | cggaattcctactgccgcagctccacat |
| pKH3-HA-cortactin-K107Q | gcagtgcttggaaagttgcgactgatattcgtggc | gccacgaatatcagtcgcaactttccaagcactgc |
| pKH3-HA-cortactin-K152Q | ccactggagtagtcttgctgggaggcatgcttc | gaagcatgcctcccagcaagactactccagtgg |
| pKH3-HA-cortactin-K171Q | cccaccgcgctctggtctactcggtcg | cgaccgagtagaccagagcgcggtggg |
| pKH3-HA-cortactin-K181Q | gtgcttctccgtctggccctggtagtcga | tcgactaccagggccagacggagaagcac |
| pKH3-HA-cortactin-K193Q | tttgccgccgaaaccttgggagtaatctctctgtg | cacagagagattactcccaaggtttcggcggcaaa |
| pKH3-HA-cortactin-K235Q | ctgtctgcacaccaaattgtcctccaaaccctttcac | gtgaaagggtttggaggacaatttggtgtgcagacag |
| pKH3-HA-cortactin-K309Q | ctgcaccccatactgcccgccgaatcctt | aaggattcggcgggcagtatggggtgcag |
| pKH3-HA-cortactin-K314Q | cttatccatccgatcctgctgcaccccatacttcc | ggaagtatggggtgcagcaggatcggatggataag |
| pKH3-HA-cortactin-K107R | cagtgcttggaaagtctcgactgatattcgtggcc | ggccacgaatatcagtcgagactttccaagcactg |
| pKH3-HA-cortactin-K152R | tggagtagtctctctgggaggcatgcttctcag | ctgagaagcatgcctcccagagagactactcca |
| pKH3-HA-cortactin-K171R | cccaccgcgctcctgtctactcggtc | gaccgagtagacaggagcgcggtggg |
| pKH3-HA-cortactin-K181R | cgtgcttctccgtcctgccctggtagtcg | cgactaccagggcaggacggagaagcacg |
| pKH3-HA-cortactin-K193R | ccgccgaaacctctggagtaatctctctgtgactc | gagtcacagagagattactccagaggtttcggcgg |
| pKH3-HA-cortactin-K235R | ctgtctgcacaccaaatcttcctccaaaccctttc | gaaagggtttggaggaagatttggtgtgcagacag |
| pKH3-HA-cortactin-K309R | ctgcaccccatacctcccgccgaatcc | ggattcggcgggaggtatggggtgcag |
| pKH3-HA-cortactin-K314R | ttatccatccgatccctctgcaccccatacttcc | ggaagtatggggtgcagagggatcggatggataa |
| pGEX-4T-1-DIP2A 1-260 | cgcggatccatggccgaccgcggatgc | tgcgcagctgttacactctgctgttcacaggc |
| pGEX-4T-1-DIP2A 1-320 | cgcggatccatggccgaccgcggatgc | tgcgcagctgagtccccactgacagaggctctc |
| pGEX-4T-1-DIP2A 261-320 | cgcggatcctcctccaaaatccagcagc | tgcgcagctgagtccccactgacagaggctctc |
| pGEX-4T-1-DIP2A 261-320 P274A/P277A | cttcagaggagcgcgctttgccctcttcaggg | ccctgaagagggcaaagcgcgctcctctgaag |
| pGEX-4T-1-DIP2A 261-320 P298A/P301A | ctcaggcttggcctggttcgcatctggttgctgaa | ttcagcaaccagatgcgaaccaggccaagcctgag |
| qRTPCR of *Dip2a* mRNA | TGGTTCCTACAGCCAGCTCTGTC | ACAGGAGCATTGCAGAGTGTGC |
| qRTPCR of *gapdh* mRNA | ATGACCCCTTCATTGACCTCA | GAGATGATGACCCTTTTGGCA |
| qRTPCR of *Atat1* mRNA | GTTACAGAAAGAGCGAGTGGA | GGTGTTGATGGGCAAAGAA |
| qRTPCR of *Crebbp* mRNA | CCCACACTGTCGAACCATG | ACAGTCATGTCGTGTGCAGT |
| qRTPCR of *Ep300* mRNA | CAGATTCCACCACAACCCCAGG | GAGGCCACACCAGCATTTTC |
| qRTPCR of *Pcaf* mRNA | AGTGCCATGGTTCCTTGTTC | CGCAGGTGAAGAGGTACTCC |
| qRTPCR of *Elp* mRNA | AAATACGTGGGACAAGGGCGCAAG | ACTTGGCAGCTGTCTTGGTTTTC |
| qRTPCR of *Hdac1* mRNA | CTATCAAAGGACACGCCAAGTG | ACCGGGCAACGTTACGAAT |
| qRTPCR of *Hdac2* mRNA | CATGGTGATGGTGTTGAAGAAG | TCATTGGAAAATTGACAGCATAGT |
| qRTPCR of *Hdac3* mRNA | TTGAGTTCTGCTCGCGTTACA | CCCAGTTAATGGCAATATCACAGAT |
| qRTPCR of *Hdac4* mRNA | AATCTGAACCACTGCATTTCCA | GGTGGTTATAGGAGGTCGACACT |
| qRTPCR of *Hdac5* mRNA | TTGGAGACGTGGAGTACCTTACAG | GACTAGGACCACATCAGGTGAGAAC |
| qRTPCR of *Hdac6* mRNA | TGGCTATTGCATGTTCAACCA | GTCGAAGGTGAACTGTGTTCCT |
