# Supplementary material for: Autism candidate gene DIP2A regulates spine morphogenesis via acetylation of cortactin
Source: PLoS Biol. 2019 Oct 10;17(10):e3000461. doi: 10.1371/journal.pbio.3000461 (PMC6786517; doi:10.1371/journal.pbio.3000461)
Supplement: S6 Table — (DOCX) [file pbio.3000461.s010.docx]

| **S6 Table. Antibodies used in this study.** | |  | |  |
| --- | --- | --- | --- | --- |
| Antibody | Corporation | | Catalogue No. | Dilution Ratio |
| Rabbit anti-DIP2A | Home-made | | - | 1:2000 for WB |
|  |  |  |  | 1:500 for ICC and IHC |
|  |  |  |  | 1:200 for IP |
| Rabbit anti-DIP2B | Sigma-Aldrich, St. Louis, USA | | SAB2700075 | 1:2000 for WB |
| Rabbit anti-DIP2C | Sigma-Aldrich, St. Louis, USA | | HPA030264 | 1:2000 for WB |
| Mouse anti-α-tub | Proteintech, Chicago, USA | | 66031 | 1:5000 for WB |
| Mouse anti-α-GAPDH | Proteintech, Chicago, USA | | HC301 | 1:5000 for WB |
| Rabbit anti-CUX1 | Santa Cruz Biotechnology, Texas, USA | | Sc-101003 | 1:2000 for ICH |
| Rat anti-CTIP2 | Abcam, Cambridge, UK | | ab18465 | 1:1000 for ICH |
| Rabbit anti-FoxP2 | Abcam, Cambridge, UK | | 16046 | 1:500 for ICH |
| Mouse anti-MAP2 | Sigma-Aldrich, St. Louis, USA | | M1406 | 1:1000 for ICC |
| Rabbit anti-GFP | Abcam, Cambridge, UK | | 6556 | 1:1000 for ICC and IHC |
| Rabbit anti-PSD95 | Abcam, Cambridge, UK | | 18258 | 1:2000 for ICC and WB |
| Rabbit anti-Synapsin I | Merck Millipore, Massachusetts, USA | | AB1542 | 1:2000 for ICC and WB |
| Mouse anti-NMDAR1 | Synaptic System, Goettingen, Germany | | 114011 | 1:500 for WB |
| Rabbit anti-NMDAR2A | R&D Systems, Minneapolis, USA | | PPS012 | 1:1000 for WB |
| Rabbit anti-NMDAR2B | Abcam, Cambridge, UK | | 65783 | 1:2000 for WB |
| Rabbit anti-GluR1 | Merck Millipore, Massachusetts, USA | | MAB2263 | 1:2000 for WB |
| Rabbit anti-GluR2 | Abcam, Cambridge, UK | | Ab206293 | 1:2000 for WB |
| Mouse anti-β-actin | Sigma-Aldrich, St. Louis, USA | | A1978 | 1:5000 for WB |
| Mouse anti-FLAG | Sigma-Aldrich, St. Louis, USA | | F7425 | 1:3000 for WB |
|  |  |  |  | 1:1000 for IP |
| Mouse anti-cortactin | Merck Millipore, Massachusetts, USA | | 05-180 | 1:2000 for WB |
|  |  |  |  | 1:500 for IP |
| Mouse anti-HA | Abcam, Cambridge, UK | | Ab16918 | 1:2000 for WB |
|  |  |  |  | 1:1000 for IP |
| Rabbit anti-acetylated cortactin | Merck Millipore, Massachusetts, USA | | 09-881 | 1:500 for WB |
| Rabbit anti-phosphorylated cortactin | Abcam, Cambridge, UK | | 47768 | 1:500 for WB |
| Rabbit anti-acetylated lysine | Cell Signaling Technology, Massachusetts, USA | | 9441 | 1:2000 for WB |
| Mouse anti-Histone 3 | Active Motif, California, USA | | 61475 | 1:2000 for WB |
| Rabbit anti-acetylated-Histone3K9 | Merck Millipore, Massachusetts, USA | | 07-352 | 1:5000 for WB |
| Rabbit anti-acetylated-Histone3K14 | Merck Millipore, Massachusetts, USA | | 07-353 | 1:2000 for WB |
| Rabbit anti-acetylated-Histone3K18 | Merck Millipore, Massachusetts, USA | | 07-354 | 1:10000 for WB |
| Rabbit anti-acetylated-Histone3K27 | Merck Millipore, Massachusetts, USA | | 07-360 | 1:20000 for WB |
| Rabbit anti-acetylated-Histone4K5 | Merck Millipore, Massachusetts, USA | | 07-327 | 1:2000 for WB |
| Rabbit anti-acetylated-Histone4K8 | Merck Millipore, Massachusetts, USA | | 07-328 | 1:1000 for WB |
| Rabbit anti-acetylated-Histone4K12 | Merck Millipore, Massachusetts, USA | | 07-595 | 1:5000 for WB |
| Rabbit anti-acetylated-Histone4K16 | Merck Millipore, Massachusetts, USA | | 07-329 | 1:10000 for WB |
| Rabbit anti-Histone4 | PTM-Bio, Hangzhou, China | | PTM-1003 | 1:1000 for WB |
