# Supplementary material for: Autism candidate gene DIP2A regulates spine morphogenesis via acetylation of cortactin
Source: PLoS Biol. 2019 Oct 10;17(10):e3000461. doi: 10.1371/journal.pbio.3000461 (PMC6786517; doi:10.1371/journal.pbio.3000461)
Supplement: S7 Table — (DOCX) [file pbio.3000461.s011.docx]

**S7 Table. Statistical analysis results.**

| Fig. | Parameter | n | Statistical Test | Significance |
| --- | --- | --- | --- | --- |
| 2B | spine density | EPN, WT = 59 neurons, KO = 75 neurons |  | *t* (132) = 3.831, *P* = 0.0002 |
|  |  | IPN, WT = 71 neurons, KO = 64 neurons |  | *t* (133) = 5.819, *P* < 0.0001 |
| 2C | spine catalogue | EPN, WT = 59 neurons, KO = 75 neurons |  | thin, *t* (132) = 0.6491, *P* = 0.5174; stubby, *t* (132) = 2.004, *P* = 0.0471; mushroom-like, *t* (132) = 2.134, *P* = 0.0347; branched, *t* (132) = 0.6106, *P* = 0.5425 |
|  |  | IPN, WT = 71 neurons, KO = 64 neurons | Two-tailed unpaired *t* test | thin, *t* (133) = 1.0708, *P* = 0.2862; stubby, *t* (133) = 3.1443, *P* = 0.0020; mushroom-like, *t* (133) = 2.8145, *P* = 0.0056; branched, *t* (133) = 1.1087, *P* = 0.2694 |
| 2E | spine width | WT = 203 spines, KO = 183 spines |  | *t* (384) = 5.991, *P* < 0.0001 |
| 2F | spine length | WT = 203 spines, KO = 180 spines |  | *t* (381) = 3.632, *P* = 0.0003 |
| 2G | spine width | WT = 158 spines, KO = 201 spines |  | *t* (357) = 3.864, *P* = 0.0001 |
| 2H | spine length | WT = 157 spines, KO = 198 spines |  | *t* (353) = 2.549, *P* = 0.0112 |
| 2J | sEPSC amplitude | WT = 7 mice, KO =7 mice |  | *t* (12) = 2.751, *P* = 0.0176 |
| 2K | sEPSC events internals | WT = 7 mice, KO =7 mice |  | *t* (12) = 0.2820, *P* = 0.7828 |
| 2M | protein level | 4 independent experiments |  | NR1, *t* (18) = 2.274, *P* = 0.0355; NR2A, *t* (10) = 4.156, *P* = 0.0020; NR2B, *t* (10) = 2.248, *P* = 0.0484; GluR1, *t* (18) = 1.082, *P* = 0.0447; GluR2, *t* (18) = 0.8829, *P* = 0.3889; PSD95, *t* (10) = 0.0759, *P* = 0.9410 |
| 3F | protein level in cortex lysate | 3 independent experiments | Two-tailed unpaired *t* test | ac-cortactin, *t* (10) = 2.320, *P* = 0.0428; p-cortactin, *t* (10) = 0.8637, *P* = 0.4080; cortactin, *t* (10) = 1.712, *P* = 0.1177; ac-tub, *t* (10) = 0.1943, *P* = 0.8498 |
| 3G | protein level in neurons lysate | 3 independent experiments; ~5*10^6 cells/experiments |  | ac-cortactin, *t* (10) = 3.258, *P* = 0.0086; p-cortactin, *t* (10) = 0.1551, *P* = 0.8798; cortactin, *t* (10) = 0.4124, *P* = 0.6888; ac-tub, *t* (10) = 0.1507, *P* = 0.8832 |
| 3I | ac-cortactin level | 3 independent experiments | One-way ANOVA,  Post Hoc-LSD | *F* (3, 20) = 14.62, *P* < 0.0001; control vs DIP2A, *P* = 0.0415; control vs HDAC6, *P* = 0.0059; control vs TSA, *P* = 0.0026 |
| 3J | ac-tub level | 3 independent experiments |  | *F* (3, 20) = 13.31, *P* = 0.0018; control vs DIP2A, *P* = 0.4099; control vs HDAC6, *P* = 0.0001; control vs TSA, *P* = 0.0379 |
| 4F | protein level | 3 independent experiments | Pearson correlation analysis | ac-cortactin, Pearson correlation = -0.656, *P* = 0.0205; ac-tub, Pearson correlation = 0.279, *P* = 0.3798 |
| 4H | protein level | 3 independent experiments |  | ac-cortactin, Pearson correlation = -0.814, *P* = 0.0013; ac-tub, Pearson correlation = 0.181, *P* = 0.5729 |
| 5A | Ac-CoA level | WT = 9 mice; KO = 9 mice | Two-tailed unpaired *t* test | *t* (16) = 2.291, *P* = 0.0359 |
| 5C | ac-cortactin level | 4 independent experiments | Pearson correlation analysis | Pearson correlation = 0.5599, *P* = 0.0021 |
| 5E | ac-cortactin level | 3 independent experiments |  | Pearson correlation = 0.5503, *P* = 0.0175 |
| 6D | PSD95 clustering | WT = 64 neurons, KO + control = 46, KO + 8KQ = 51 | One-way ANOVA, Post Hoc-LSD  One-way ANOVA, Post Hoc-LSD | *F* (2, 148) = 16.56, *P* = 0.0179; WT vs KO + control, *P* < 0.0001; WT vs KO + 8kq, *P* = 0.0513 |
| 6F | mEPSC amplitude | WT = 16 neurons, KO = 14, KO + control = 20, KO + 8KQ = 21 |  | F (3, 65) = 34.16, *P* < 0.0001; WT vs KO, *P* < 0.0001; WT vs KO + control, *P* < 0.0001; WT vs KO + 8KQ, *P* = 0.1521 |
| 6G | mEPSC events interval | WT = 16 neurons, KO = 14, KO + control = 20, KO + 8KQ = 21 |  | *F* (3, 61) = 1.01, *P =* 0.3946; WT vs KO, *P =* 0.5107; WT vs KO + control, *P =* 0.9482; WT vs KO + 8KQ, *P* = 0.0794 |
| 7B | call duration | WT = 25 mice; KO = 24 mice | Two-tailed unpaired *t* test | *t* (47) = 2.711, *P* = 0.0093 |
| 7C | time spent calling | WT = 25 mice; KO = 24 mice |  | *t* (47) = 2.716, *P* = 0.0092 |
| 7D | self-grooming in open field | WT = 14 mice; KO = 14 mice |  | *t* (26) = 2.651, *P* = 0.0135 |
| 7E | self-grooming in open field | WT = 14 mice; KO = 14 mice |  | 0-5 min, *t* (26) = 3.104, *P* = 0.0046; 5-10 min, *t* (26) = 2.425, *P* = 0.0226; 10-15 min, *t* (26) = 2.456, *P* = 0.0210 |
| 7F | self-grooming in home cage | WT = 18 mice; KO = 18 mice |  | *t* (34) = 2.508, *P* = 0.0171 |
| 7G | digging marbles time | WT = 14 mice; KO = 15 mice |  | *t* (27) = 2.386, *P* = 0.0243 |
| 7H | exploration time in sociability test | WT = 17 mice; KO = 16 mice |  | WT, *t* (32) = 2.847, *P* = 0.0076; KO, *t* (30) = 2.982, *P* = 0.0056 |
| 7I | exploration time in social novelty test | WT = 17 mice; KO = 16 mice |  | WT, *t* (32) = 4.234, *P* = 0.0002; KO, *t* (30) = 0.8245, *P* = 0.4161 |
| 7L | self-grooming time in home cage | WT + control = 11 mice, KO + control = 9 mice, KO + 8KQ =8 mice | One-way ANOVA, Post Hoc-LSD | *F* (2, 25) = 5.238, *P* = 0.013; WT + control vs KO + control, *P* = 0.0038*;* WT + control vs KO + 8KQ, *P* = 0.4001; KO + control vs KO+8KQ, *P* = 0.0436 |
| S3B | protein level | WT = 4 mice; KO = 4 mice | Two-tailed unpaired *t* test | DIP2A, *t* (6) = 45.14, *P* < 0.0001; DIP2B, *t* (6) = 0.1713, *P* = 0.8696; DIP2C, *t* (6) = 0.1769, *P* = 0.8654 |
| S3D | body weight | WT = 10 mice; KO = 7 mice |  | *t* (15) = 0.4353, *P* = 0.6695 |
|  | brain weight | WT = 6 mice; KO = 6 mice |  | *t* (10) = 0.0491, *P* = 0.9617 |
| S3H | cells density | WT = 3 mice; KO = 3 mice | Two-tailed unpaired *t* test | internal, *t* (11) = 0.4603, *P* = 0.6543; medium, *t* (11) = 0.7115, *P* = 0.4916; external, *t* (10) = 0.0491, *P* = 0.9620 |
| S3I | cells density | WT = 3 mice; KO = 3 mice |  | internal, *t* (10) = 0.5516, *P* = 0.5933; medium, *t* (11) = 0.4905, *P* = 0.6334; external, *t* (10) = 1.223, *P* = 0.2495 |
| S3K | positive cells percentage | WT = 3 mice; KO = 3 mice |  | II/III, *t* (4) = 1.6867, *P* = 0.1669; IV, *t* (4) = 1.7234, *P* = 0.1599; V, *t* (4) = 1.4007, *P* = 0.2339; VI, *t* (4) = 2.0915, *P* = 0.1047 |
| S4C | spine density | EPN, WT = 53 neurons, KO = 50 neurons |  | *t* (101) = 0.6437, *P* = 0.5212 |
|  |  | IPN, WT = 48 neurons, KO = 57 neurons |  | *t* (103) = 1.2237, *P* = 0.2188 |
| S4D | spine catalogue | EPN, WT = 86 neurons, KO = 73 neurons |  | thin, *t* (157) = 0.1089, *P* = 0.9134; stubby, *t* (157) = 0.7013, *P* = 0.4841; mushroom-like, *t* (157) = 0.1929, *P* = 0.8473; branched, *t* (157) = 0.3135, *P* = 0.7543 |
|  |  | IPN, WT = 69 neurons, KO = 64 neurons |  | thin, *t* (131) = 0.2490, *P* = 0.8038; stubby, *t* (131) = 0.3601, *P* = 0.7194; mushroom-like, *t* (131) = 0.1328, *P* = 0.8946; branched, *t* (131) = 1.1216, *P* = 0.2641 |
| S4E | protein level | WT = 4 mice; KO = 4 mice |  | NR1, *t* (10) = 0.1293, *P* = 0.8997; NR2A, *t* (10) = 0.5385 *P* = 0.6020; NR2B, *t* (10) = 1.321, *P* = 0.2159; GluR1, *t* (14) = 1.167, *P* = 0.2626; GluR2, *t* (10) = 0.2259, *P* = 0.8258; PSD95, *t* (14) = 0.7541, *P* = 0.4633 |
| S5B | protein level | WT = 4 mice; KO = 4 mice |  | *t* (10) = 0.5322, *P* = 0.6062 |
| S5D | protein level | WT = 4 mice; KO = 4 mice |  | H3K9ac, *t* (12) = 1.3013, *P* = 0.2176; H3K14ac, *t* (12) = 0.1595 *P* = 0.8760; H3K18ac, *t* (24) = 0.9512 *P* = 0.3510; H4K5ac, *t* (22) = 1.716, *P* = 0.1003; H4K27ac, *t* (18) = 1.374, *P* = 0.1863; H4K8, *t* (16) = 0.7130, *P* = 0.4861; H4K12ac, *t* (18) = 0.3287, *P* = 0.7462; H4K16, *t* (18) = 1.8250, *P* = 0.0847 |
| S6A | relative mRNA level | WT = 3 mice; KO = 3 mice | Two-tailed unpaired *t* test | MEC17, *t* (6) = 0.3362, *P* = 0.7482; CBP, *t* (4) = 0.1743, *P* = 0.8701; P300, *t* (6) = 0.2675, *P* = 0.7980; PCAF, *t* (6) = 0.1900, *P* = 0.8556; ELP3, *t* (6) = 0.3382, *P* = 0.7468 |
| S6G | mEPSC amplitude | WT + control = 12 neurons, WT + 8KQ = 20 neurons |  | *t* (25) = 0.9964, *P* = 0.3286 |
| S6H | mEPSC events interval | WT + control = 12 neurons, WT + 8KQ = 20 neurons |  | *t* (25) = 0.3016, *P* = 0.7655 |
| S6J | mEPSC amplitude | WT + control = 17 neurons, KO + control = 16, KO + cortactin = 18 | One-way ANOVA, Post Hoc-LSD | *F* = 62.85, *P* < 0.0001; WT + control vs KO + control, *P* < 0.0001; WT + control vs KO + cortactin, *P* < 0.0001; KO + control vs KO + cortactin, *P* = 0.039 |
| S6K | mEPSC events interval | WT + control = 16 neurons, KO + control = 17, KO + cortactin = 19 |  | *F* = 1.189, *P* = 0.313 |
| S7D | PSD95 clustering | WT = 19 neurons, KO + control = 22, KO + FL = 18 | One-way ANOVA, Post Hoc-LSD | *F* = 4.327, *P* = 0.0179; WT vs KO + control, *P* = 0.0104; WT vs KO + FL, *P* = 0.7967; KO + control vs KO + FL, *P* = 0.0263 |
| S7F | EPSC amplitude | WT= 32 neurons, KO = 22 neurons | Two-tailed unpaired *t* test | *t* (52) = 4.189, *P* = 0.0001 |
| S7H | mEPSC amplitude | KO + control = 15 neurons, KO + FL = 20 neurons |  | *t* (33) = 18.13, *P* < 0.0001 |
| S7I | mEPSC events interval | KO + control = 15 neurons, KO + FL = 20 neurons |  | *t* (33) = 0.0823, *P* = 0.9349 |
| S8A | rearing in open field | WT = 14 mice; KO = 14 mice | Two-tailed unpaired *t* test | *t* (26) = 0.1138, *P* = 0.9103 |
|  | self-grooming in open field | WT = 14 mice; KO = 14 mice |  | *t* (26) = 2.023, *P* = 0.0535 |
| S8D | latency for buried food | WT = 14 mice; KO = 14 mice |  | *t* (26) = 0.0318, *P* = 0.9748 |
| S8J | exploration time in sociability test | WT + control = 11 mice, KO + control = 9 mice, KO + 8KQ = 8 mice |  | WT + control, *t* (20) = 3.914, *P* = 0.0009; KO + control, *t* (16) = 2.603, *P* = 0.0192; KO+8KQ, *t* (14) = 2.186, *P* = 0.0463 |
| S8L | exploration time in social novelty test | WT + control = 11 mice, KO + control = 9 mice, KO + 8KQ = 8 mice |  | WT + control, *t* (20) = 4.758, *P* = 0.0001; KO + control, *t* (16) = 0.5021, *P* = 0.6224; KO + 8KQ, *t* (14) = 1.088, *P* = 0.2950 |
