# Supplementary figures and images for: Autism candidate gene DIP2A regulates spine morphogenesis via acetylation of cortactin
Source: PLoS Biol. 2019 Oct 10;17(10):e3000461. doi: 10.1371/journal.pbio.3000461 (PMC6786517; doi:10.1371/journal.pbio.3000461)

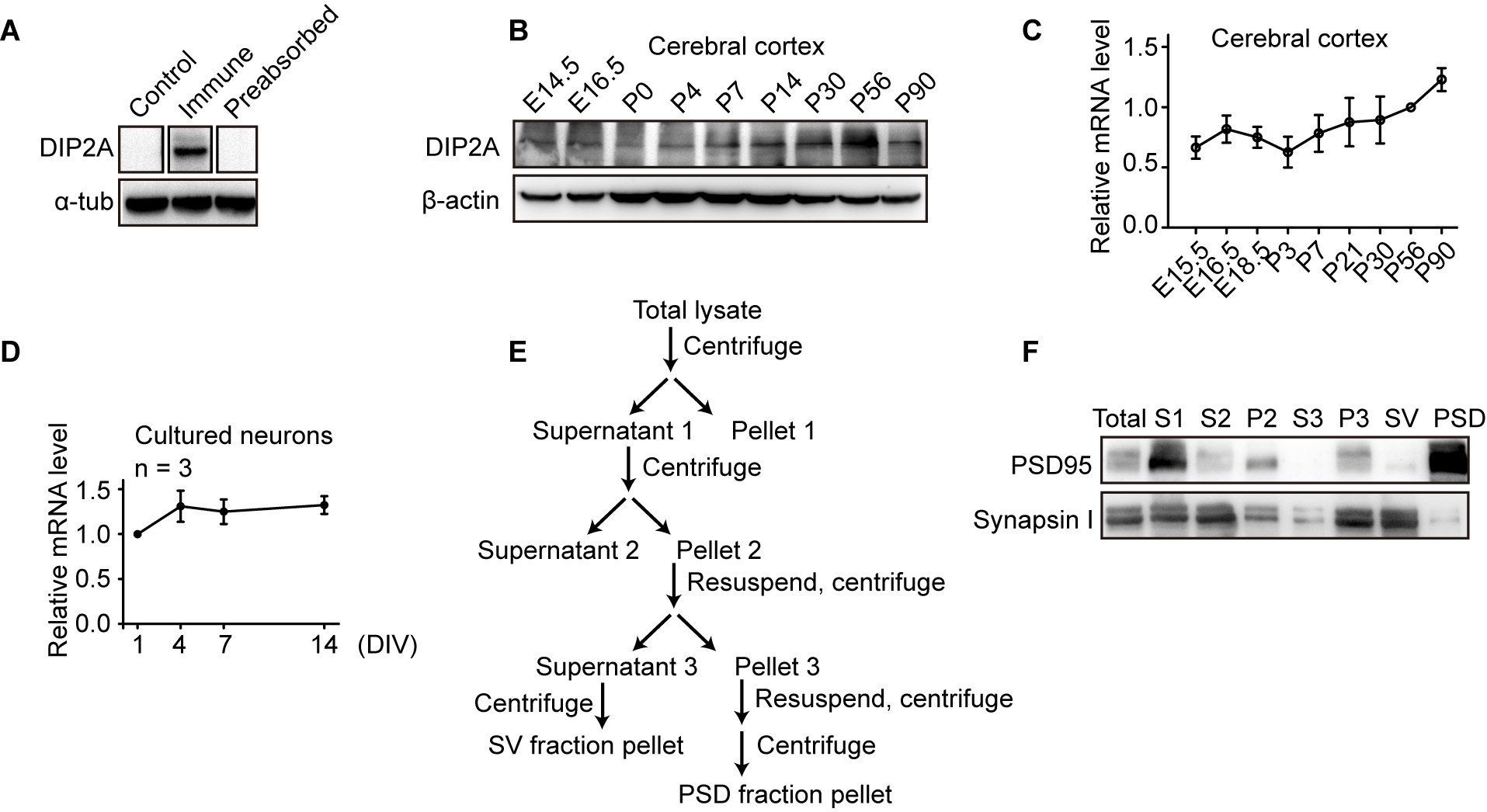

Supplement: S1 Fig — (A) Purified anti-DIP2A antibody strongly recognized a single protein band at the predicted size (approximately 180 kDa) in western blotting of C57/BL6 brain homogenates, while pre-immune serum or antibody after antigen binding could not. (B) Western blotting showing the level of DIP2A in cerebral cortex from the embryonic stage to adult, with constant increase during postnatal development. (C and D) Real-time PCR analysis showing the increasing relative Dip2a mRNA levels in cerebral cortex (C) and cultured neurons (D). Gapdh was used as normalized control. (E) Flowchart for PSD protein extraction (detailed information descripted in S1 Text). (F) Subcellular fractions from PSD protein extraction were detected by immunoblotting. Total, total homogenate; S, supernatant; P, pellet; SV, crude synaptic vesicle fraction. The underlying data for this figure can be found in S1 Data. DIP2A, disconnected-interacting protein homolog 2 A; DIV, day in vitro; E, embryo day; Gapdh, glyceraldehyde-3-phosphate dehydrogenase; P, postnatal day; PSD, postsynaptic density. (TIF) [file pbio.3000461.s014.tif]

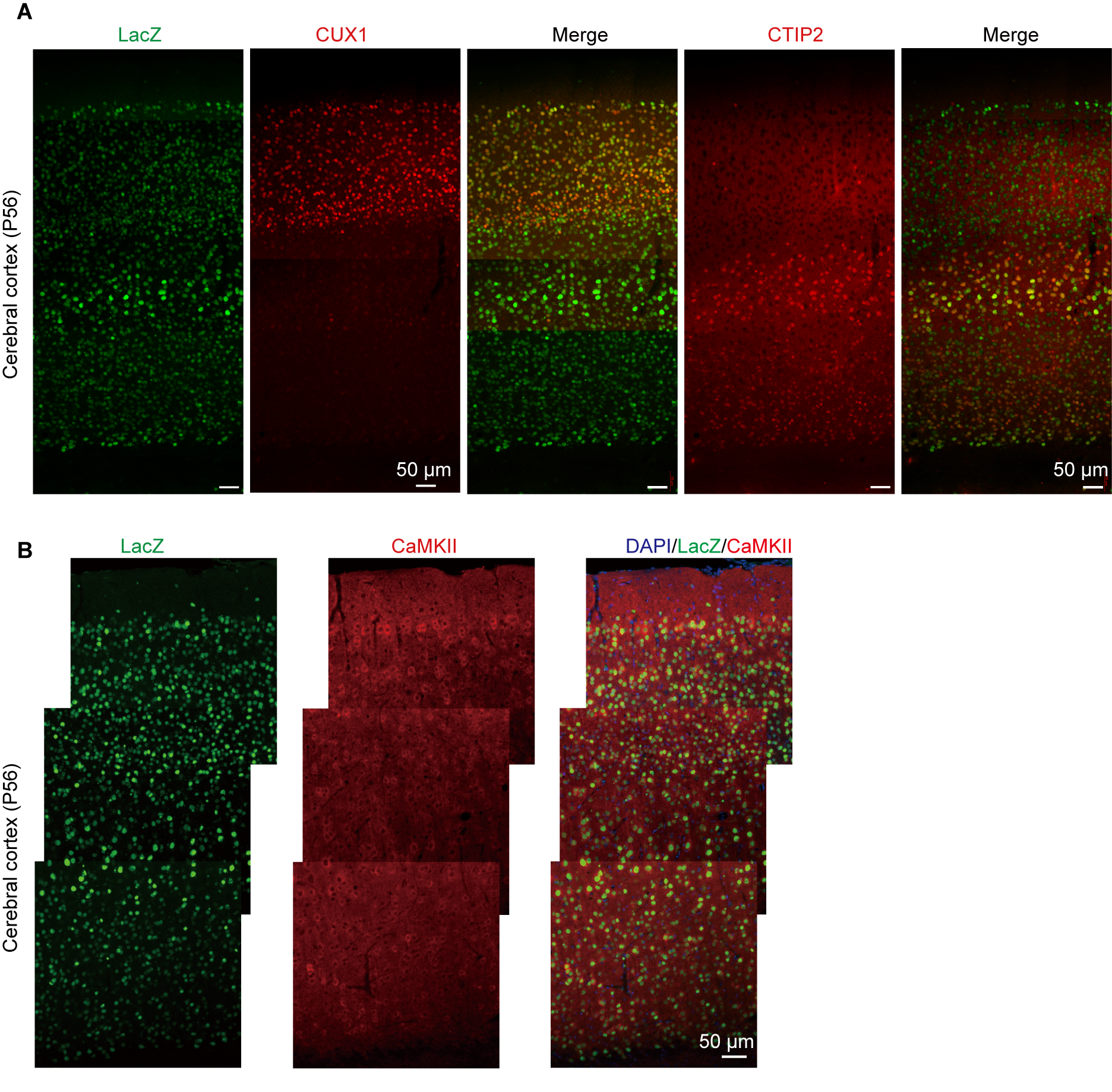

Supplement: S2 Fig — (A) LacZ reporter mice (Dip2alacZ/+) were used to display the endogenous DIP2A-expressed cell type. CUX1 and CTIP2 were stained in cortical sections to label pyramidal neurons in external and internal layers, respectively. Staining of CTIP2 has been applied with pseudocolor for better display of colocalization. (B) CaMKII was stained in cortical sections of Dip2alacZ/+ mice to label excitatory neurons. CaMKII, Ca2+/calmodulin-dependent protein kinase II; CTIP2, B cell leukemia/lymphoma 11B; CUX1, cut-like homeobox 1; DIP2A, disconnected-interacting protein homolog 2 A; LacZ, Dip2a β-galactosidase. (TIF) [file pbio.3000461.s015.tif]

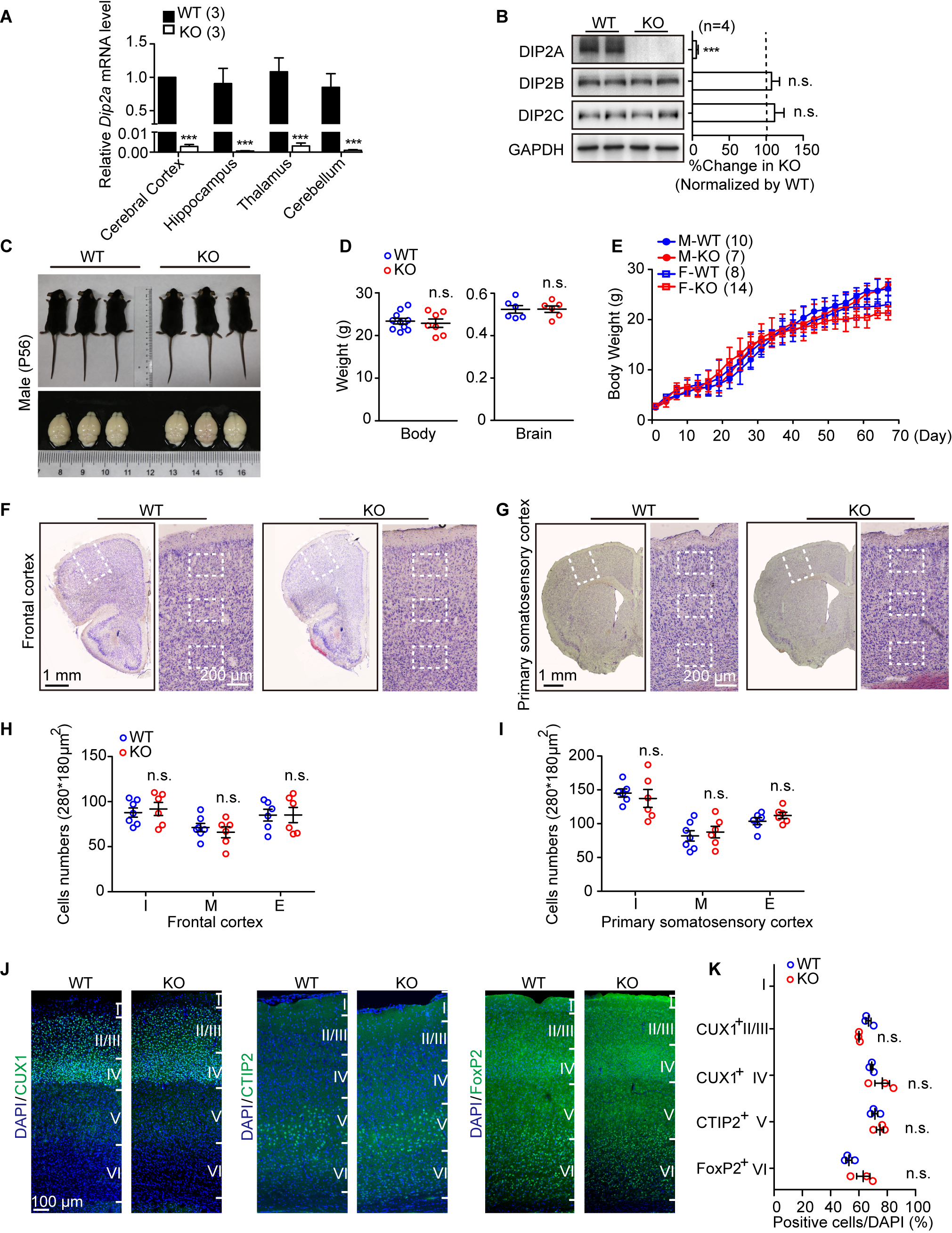

Supplement: S3 Fig — (A) Relative Dip2a mRNA levels in WT and KO brains (P56, males, 3 mice per genotype; t test, ***P < 0.0001). (B) Western blot demonstrating the specificity of the antibody against DIP2A in the Dip2a KO mice (4 mice per genotype for each experiment, data from 3 independent experiments). No significant changes were detected in DIP2B or DIP2C expression in Dip2a KO mice, meaning no distinct compensation effect from Dip2a KO. (C) The gross body and brain morphology of WT and Dip2a KO littermates (P56, males). (D) Quantification of body (P56, males, WT = 10, KO = 7; t15 = 0.4353, n.s. P = 0.6699) and brain weight (P56, males, n = 6 mice per genotype; t10 = 0.0491, n.s. P = 0.9617). (E) Line graph showing no difference between the body weight gain of WT and Dip2a KO littermates over a 70-day period (M, male; F, female). (F and G) Nissl staining of adult brain slices (P56, males, 3 mice per genotype). The three dashed white boxes represent different regions of cortex; from top to bottom are the external, the medium, and the internal parts. (H and I) Quantitative data of the cell density of Nissl-stained sections of S3F and G Fig. Cell density was measured using a fixed rectangular matrix of 180 × 280 μm2. E, external; M, medium; I, internal (n = 3 mice per genotype, 2 slices were captured each mouse; I, t11 = 0.4603, P = 0.6543; t11 = 0.7115, P = 0.4916; t10 = 0.0491, P = 0.9620; J, t10 = 0.5516, P = 0.5933; t11 = 0.4905, P = 0.6334; t10 = 1.223, P = 0.2495). (J) Immunostaining images from cortical sections of WT and Dip2a KO animals (P56, males; n = 3 mice per genotype). Different layer-specific markers were used to label the lamellar cortex: CUX1, layer II-IV; CTIP2, layer V; FoxP2, layer VI. (K) Quantitative data obtained from immunostaining of cortical layers. The percentage of positive markers in DAPI staining for (J) was assessed (II/III, t4 = 1.6867, P = 0.1669; IV, t4 = 1.7234, P = 0.1599; V, t4 = 1.4007, P = 0.2339; VI, t4 = 2.0915, P = 0.1047). The underlying dat [file pbio.3000461.s016.tif]

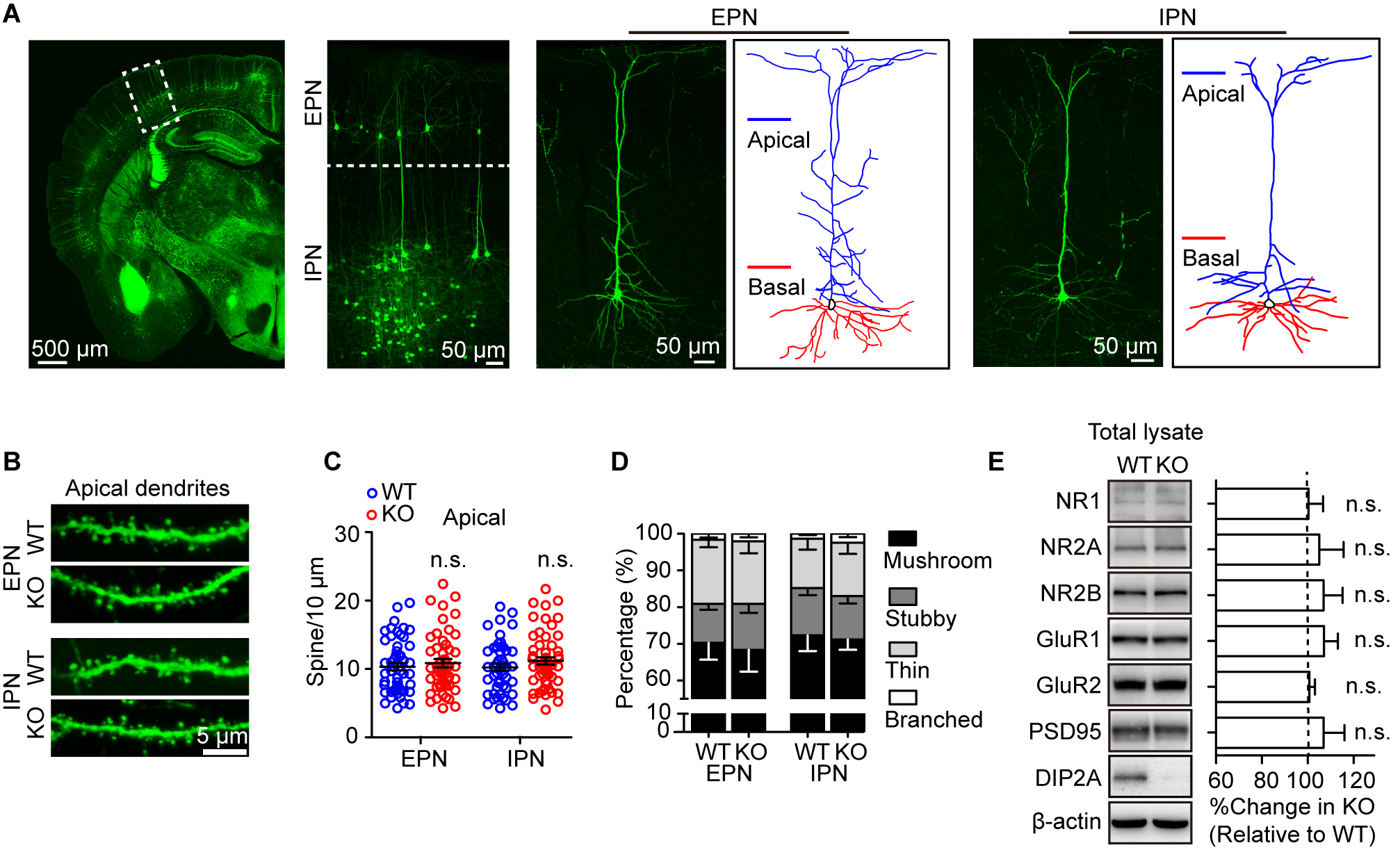

Supplement: S4 Fig — (A) Representative figure of GFP-labeled cerebral cortex and pyramidal neurons from Dip2a KO × Thy1-GFP offspring. EPN and IPN are distinguished by white dotted line. In the right schematic, apical (blue line) and basal dendrites (red line) of pyramidal neurons are represented by different colors. (B) Representative images of GFP-labeled apical dendrites spines. Only the secondary and third dendrites were captured. (C) Quantification of confocal pictures showing spine densities of apical dendrites (EPN, WT = 53 neurons, KO = 50 neurons; t101 = 0.6437, P = 0.5212; IPN, WT = 48 neurons, KO = 57 neurons; t103 = 1.2237, P = 0.2188). (D) Dendritic spine classification in apical dendrites of external and internal layer neurons. (E) Western blot showing protein levels of glutamate receptor subunits in total cortical lysates. The histograms showing quantified protein expression (normalized to β-actin) in Dip2a KO mice. The ratio in WT mice was set to 100% (P56, males; 4 mice per genotype in each experiment; data from 3 independent experiments; n.s. P > 0.05). The underlying data for this figure can be found in S1 Data. Dip2a, disconnected-interacting protein homolog 2 A; EPN, external layer of pyramidal neurons; GFP, green fluorescence protein; IPN, internal layer of pyramidal neurons; KO, knockout; n.s., no significance; Thy1, thymocyte antigen 1; WT, wild-type. (TIF) [file pbio.3000461.s017.tif]

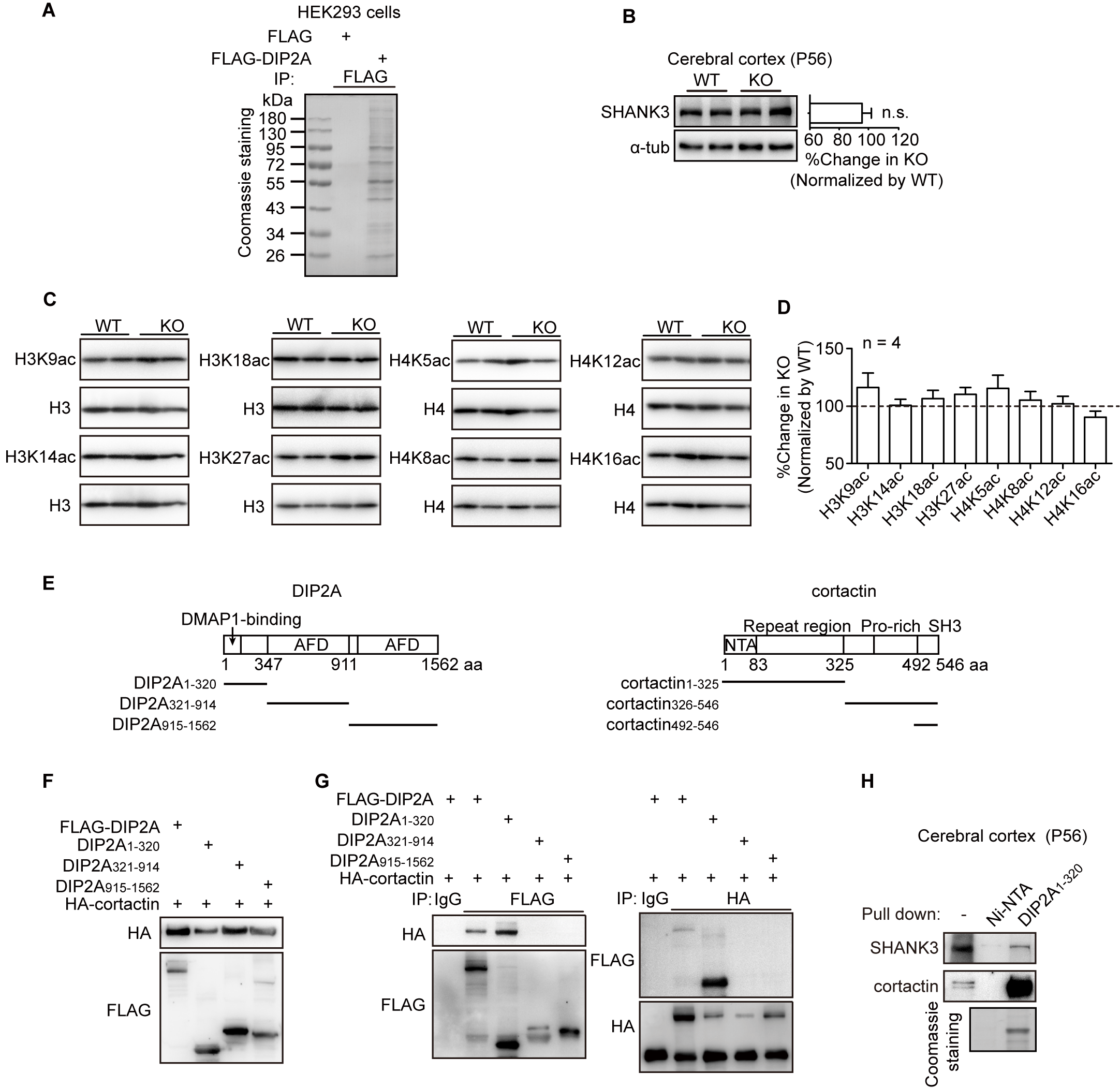

Supplement: S5 Fig — (A) FLAG-DIP2A was overexpressed into cultured HEK293 cells and purified with anti-FLAG M2 Affinity Gel. All the positive lanes that isolated with SDS-PAGE were subjected to LC-MS/MS. (B) Western blot and histogram showing level of SHANK3 protein. (C and D) Western blot and histogram showing no significant difference in acetylated histones levels between WT and Dip2a KO cerebral cortex (P56, males; 4 mice per genotype for each experiment; data from 3 independent experiments). Quantified acetylated protein level is normalized to total histone expression. The ratio in WT mice was set to 100%. The P values determined by two-tailed t test are 0.3747, 0.9998, 0.7325, 0.1254, 0.5839, 0.5959, 0.8905, and 0.1166, respectively. (E) Diagram of DIP2A and cortactin constructs. (F) Input of HEK293 cell lysates transfected with HA-tagged cortactin and FLAG-tagged full-length or truncated DIP2A. (G) IP and IB showing the N terminus of DIP2A (aa 1–320) interacted with cortactin. HA-cortactin was co-IPed with anti-FLAG antibody from cell lysates that were co-transfected with HA-cortactin and FLAG-tagged full-length DIP2A or N terminus–truncated DIP2A1–320. No HA-cortactin band was detected in the IP complex from cell lysates co-transfected with mock IgG, or the DIP2A321–914, DIP2A915–1,562 constructs. All input loading consisted of 1% of the lysates for IP. (H) Truncated His-DIP2A1–320 were expressed in E. coli and purified using Ni-NTA gel. Pull-down assay was performed in murine cerebral cortex lysate to detect the endogenous interaction of DIP2A, cortactin, and SHANK3. Coomassie staining showed the loading of purified DIP2A1–320. The underlying data for this figure can be found in S1 Data. aa, amino acid; co-IP, co-immunoprecipitation; DIP2A, disconnected-interacting protein homolog 2 A; FLAG, FLAG tag with the sequence DYKDDDDK; HA, human influenza hemagglutinin tag (YPYDVPDYA-tag); His, hexahistidine tag; IB, immunoblot; IgG, immunoglobulin G; IP, immunoprecipitation; KO, knock [file pbio.3000461.s018.tif]

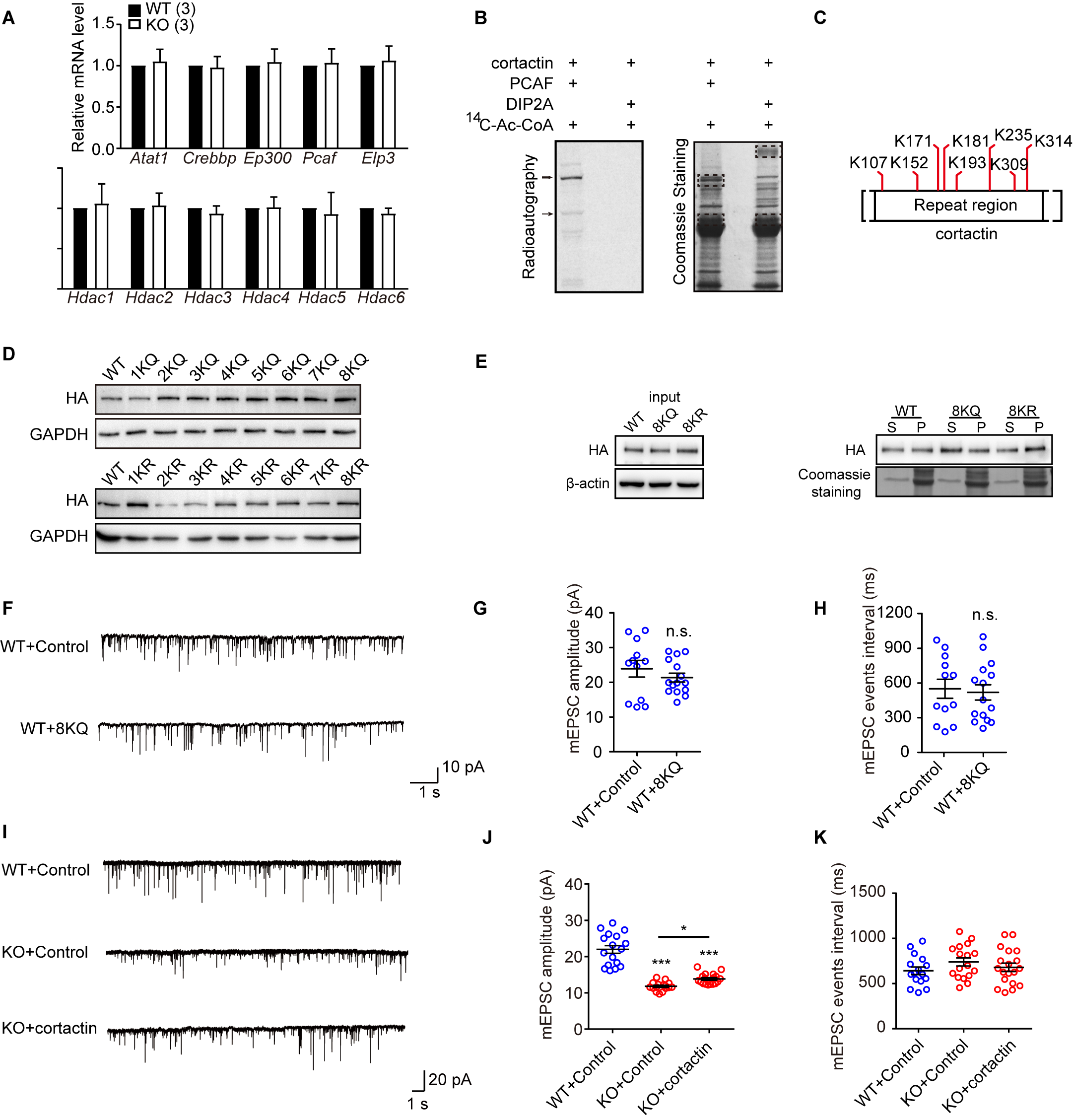

Supplement: S6 Fig — (A) Relative mRNA levels of the indicated lysine acetyltransferase and deacetylase in WT and KO brains (males, P56; 3 mice per genotype; two-tailed unpaired t test, P > 0.05). (B) Reaction products in acetylation assays in vitro were resolved by SDS-PAGE, and the acetylated cortactin (lower arrow) and acetylated PCAF (upper thicker arrow) were visualized by radioautography (lane 1). DIP2A did not directly acetylate cortactin (lane 3). Total amounts of protein were monitored by Coomassie blue staining. No sample was loaded in lane 2 to avoid mixing of samples in lanes 1 and 3. (C) Unambiguous acetylated lysine residues identified by LC-MS/MS. (D) Western blot showing successful expression each of HA-tagged WT and mutant cortactin constructs (8KQ and 8KR) in HEK293 cells by transient transfection. (E) F-actin binding ability of cortactin was modulated by its acetylation level. Ratios of gray value of cortactin strips in pellets and supernatants are 1.028, 0.781, and 1.594 for HA-cortactin (WT), acetylation-mimetic cortactin (8KQ), and non-acetylation-mimetic cortactin (8KR), respectively. (F) Representative whole-cell voltage clamp traces of mEPSC from cultured neurons held at −70 mV. WT neurons were infected with Lv-eGFP-control or Lv-eGFP cortactin 8KQ at DIV10 and traced at DIV15. Scatterplot graph showing the equivalent mEPSC amplitude (G) and frequency (H) (n = 12 and 15 neurons, respectively; data from 3 independent experiments; t test; n.s. P > 0.05). (I) Representative whole-cell voltage clamp traces of mEPSC from cultured neurons. Neurons were infected with Lv-eGFP-control or Lv-eGFP cortactin at DIV10 and traced at DIV15. Scatterplot graph showing the equivalent mEPSC amplitude (J) and frequency (K). (J, one-way ANOVA, ***P < 0.0001 compared with WT + control, *P = 0.039 compared with KO + control; K, one-way ANOVA, P = 0.313). The underlying data for this figure can be found in S1 Data. ac-cortactin, cortactin acetylation; Atat1, alpha tubulin acetyltransfe [file pbio.3000461.s019.tif]

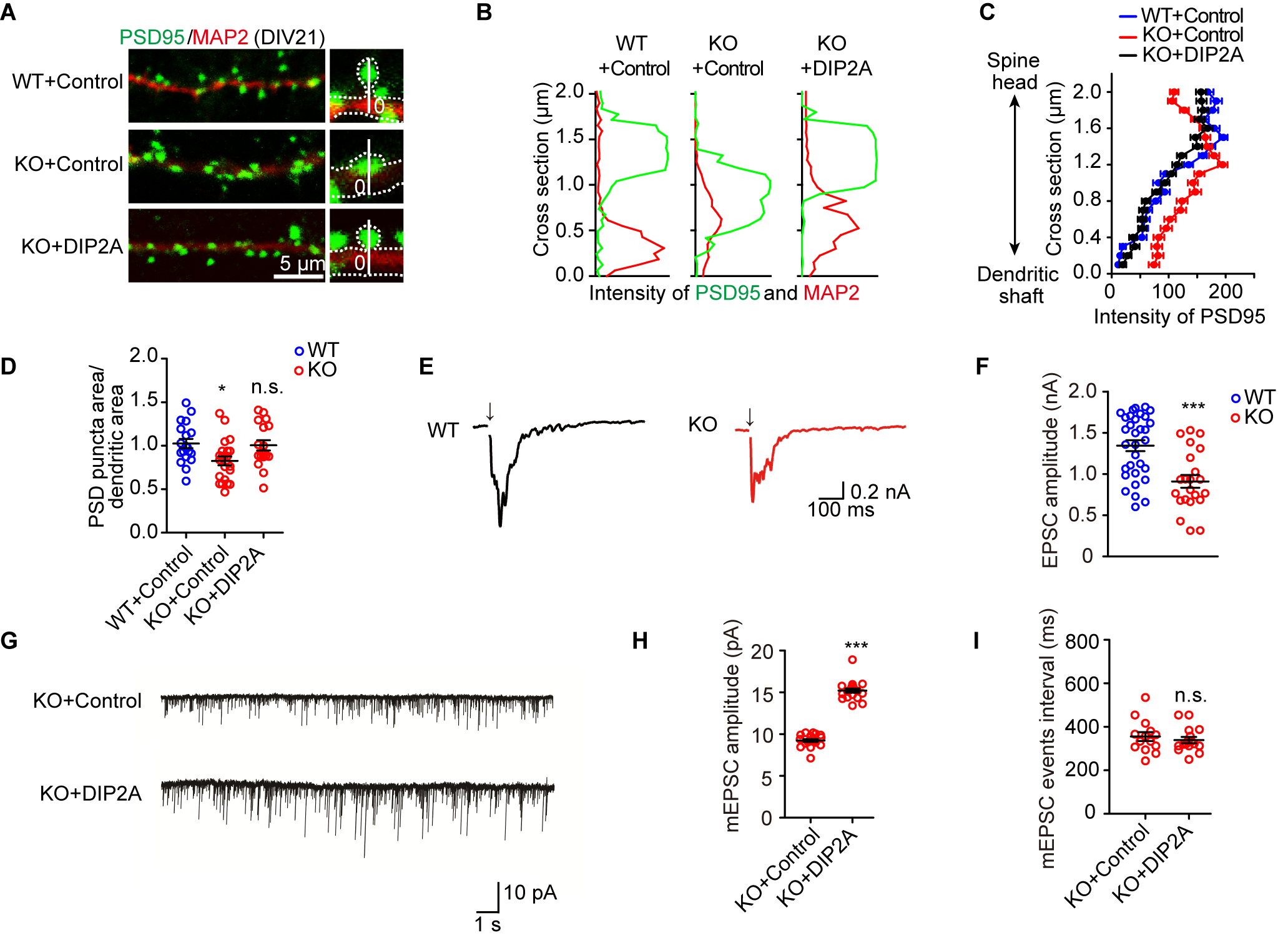

Supplement: S7 Fig — (A) Representative images of dendritic segments of cultured neurons infected with adenovirus vectors expressing eGFP-DIP2A or eGFP control. The insets to the right show an example of the quantitative analysis of PSD95 distribution along the 2-μm vertical white line, which proceeds from the base of the dendritic shaft (set as 0). The dotted line depicts the outline of PSD95. (B and C) Quantitative traces illustrating the calculated fluorescence intensity of PSD95 along the scanning path as shown in (A). (D) The ratio of PSD95-positive puncta to total dendritic (MAP2) area was lower in Dip2a KO neurons than in WT. DIP2A overexpression in KO neurons rescued the ratio to WT levels (n = 19, 22, 18 neurons, respectively; one-way ANOVA, F2, 56 = 4.327, P = 0.0179; post hoc LSD, *P = 0.0102, n.s. P = 0.7983, compared with WT + control). (E) Representative whole-cell voltage clamp traces of evoked EPSCs in response to stimulation (arrows) in cultured neurons (DIV15). (F) The amplitude of evoked EPSCs in Dip2a KO neurons (n = 32) was reduced as compared with WT neurons (n = 22) (t52 = 4.189, P = 0.0001). (G) Representative whole-cell voltage clamp traces of mEPSC from cultured neurons held at −70 mV (n = 20 neurons per genotype; data from 3 independent experiments). (H) Ad-eGFP-DIP2A infection of Dip2a KO neurons rescued the reduction in amplitude (t38 = 19.82, P < 0.0001). (I) The frequency of mEPSCs was not significantly different in Dip2a KO neurons expressing eGFP versus eGFP-DIP2A (t33 = 0.08226, n.s. P = 0.9349). The underlying data for this figure can be found in S1 Data. Ad, adenovirus; DIP2A, disconnected-interacting protein homolog 2 A; DIV, day in vitro; eGFP, enhanced green fluorescent protein tag; EPSC, excitatory postsynaptic current; KO, knockout; LSD, least significant difference; MAP2, microtubule-associated protein 2; mEPSC, miniature excitatory postsynaptic current; n.s., no significance; PSD95, postsynaptic density protein 95; WT, wild-type. (TIF) [file pbio.3000461.s020.tif]

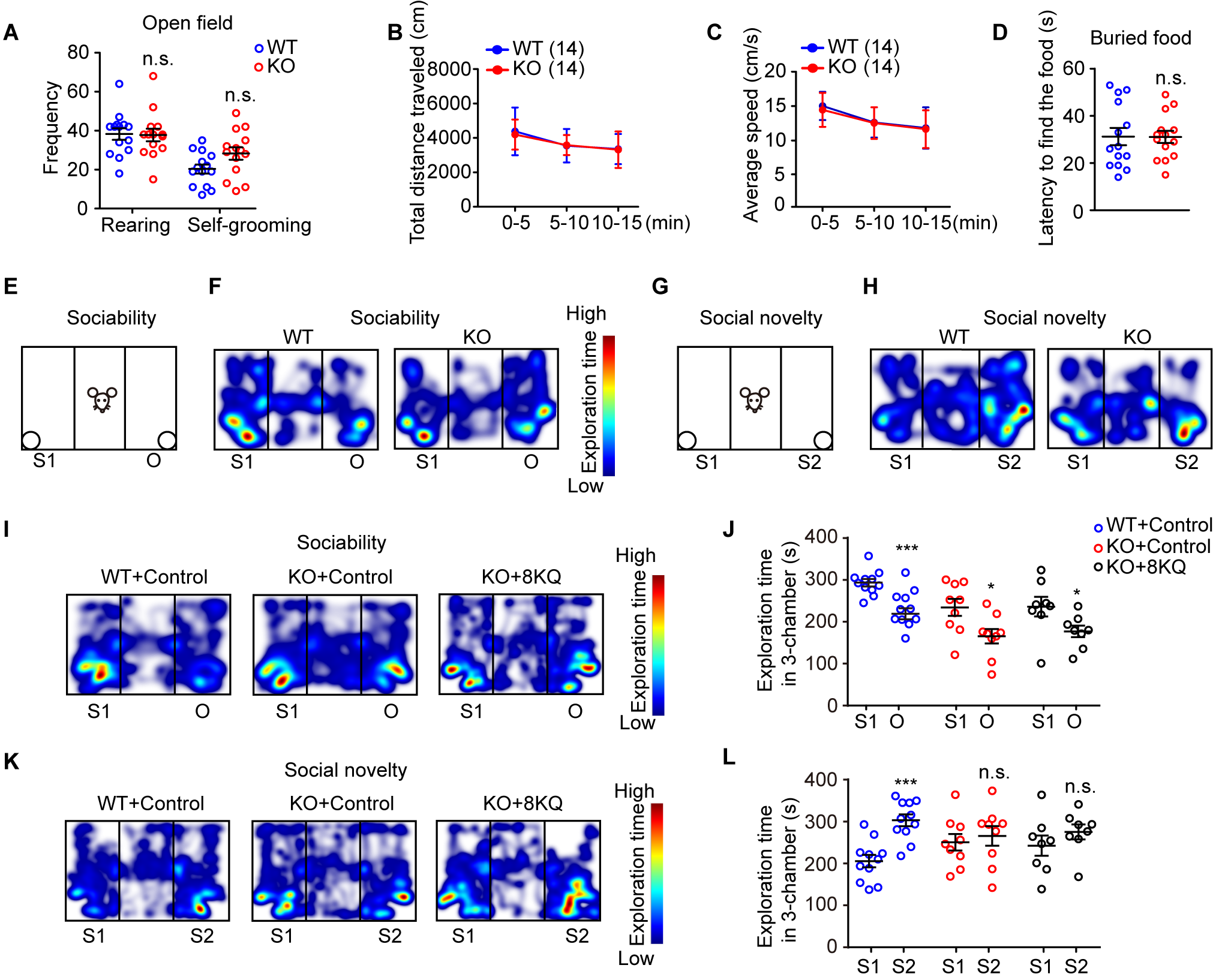

Supplement: S8 Fig — (A) Frequency of rearing and self-grooming behaviors in the open field test (14 mice per genotype; t26 = 0.1138, P = 0.9103; t26 = 2.023, P = 0.0535). Line graph showing similar locomotor distance (B) and average speed (C), indications of locomotor activity. (D) Quantification of the behavioral olfaction as assessed in the buried food test (14 mice per genotype; t26 = 0.0318, P = 0.9748). Illustration of the apparatus used for the sociability test (E) and social novelty test (G). Representative exploring heat map of WT and KO mice in sociability trial (F) and social novelty trial (H). (I) Heat maps of mice in three-chambered sociability. (J) Exploration time of mice in three-chambered sociability (WT + control = 11, KO + control = 9, KO + 8KQ = 8 mice; ***P = 0.0009, *P = 0.0192 and 0.0463, respectively). (K) Heat maps of mice in three-chambered social novelty test. (L) Exploration time of mice in three-chambered social novelty test (WT + control = 11 mice, KO + control = 9 mice, KO + 8KQ = 8 mice; ***P = 0.0001, n.s. P = 0.6224 and 0.2950, respectively). The underlying data for this figure can be found in S1 Data. KO, knockout; n.s., no significance; WT, wild-type; 8KQ, acetylation mimetic cortactin with eight lysine replaced with glutamine. (TIF) [file pbio.3000461.s021.tif]
